# Can quartet analyses combining maximum likelihood estimation and Hennigian logic overcome long branch attraction in phylogenomic sequence data?

**DOI:** 10.1371/journal.pone.0183393

**Published:** 2017-08-25

**Authors:** Patrick Kück, Mark Wilkinson, Christian Groß, Peter G. Foster, Johann W. Wägele

**Affiliations:** 1 Zoologisches Forschungsmuseum Alexander Koenig, Bonn, 53113, Germany; 2 The Natural History Museum, London, SW7 5BD, United Kingdom; 3 Delft University of Technology, Delft, 2628 CD, The Netherlands; Max F Perutz Laboratories GmbH, AUSTRIA

## Abstract

Systematic biases such as long branch attraction can mislead commonly relied upon model-based (i.e. maximum likelihood and Bayesian) phylogenetic methods when, as is usually the case with empirical data, there is model misspecification. We present *PhyQuart*, a new method for evaluating the three possible binary trees for any quartet of taxa. *PhyQuart* was developed through a process of reciprocal illumination between a priori considerations and the results of extensive simulations. It is based on identification of site-patterns that can be considered to support a particular quartet tree taking into account the Hennigian distinction between apomorphic and plesiomorphic similarity, and employing corrections to the raw observed frequencies of site-patterns that exploit expectations from maximum likelihood estimation. We demonstrate through extensive simulation experiments that, whereas maximum likeilihood estimation performs well in many cases, it can be outperformed by *PhyQuart* in cases where it fails due to extreme branch length asymmetries producing long-branch attraction artefacts where there is only very minor model misspecification.

## Introduction

Reconstructing what happened is a central task of any historical science [1]. In biology, phylogenetic relationships are an important component of the history of life, some knowledge of which is a precondition of comparative methods [2]. The centrality of phylogeny in biology justifies the substantial continuing interest in reconstructing the Tree of Life, e.g. [3–5].

Modern techniques of nucleotide sequencing and the exponential growth of molecular databases increasingly provide data sets featuring hundreds of species and thousands of nucleotides in phylogenetic studies. The availability of whole genomes in the order of billions of nucleotides makes all-encompassing phylogenetic analyses possible for the first time [6]. The new age of phylogenomics gives reason to hope that congruence in phylogenetic analysis can finally be achieved through the reduction of stochastic sampling errors [7]. However, there is considerable concern about increased accumulation of systematic errors due to reliance upon simple substitution models that may not adequately consider variation in substitution rate, compositional heterogeneity and the erosion of phylogenetic signal, e.g. [8–13] and which may be inconsistent.

Generally, systematic errors are increasingly important and apparent as more data are analysed because stochastic effects become less prominent, eventually yielding maximally supported, but incorrectly resolved phylogenetic relationships [14–16]. Numerous studies have shown that model misspecification can reduce the accuracy of phylogeny inference, e.g. [11–13, 17–31].

Systematic bias is particularly a molecular data problem due to the small number of possible character states [32] and the absence of complexity that might otherwise allow better distinction between homologies and homoplasies. Recent phylogenomic studies demonstrate how sensitive probabilistic tree reconstruction methods are to model assumptions and data composition. For example, the position of myriapods within the arthropod tree of life [33–37], the phylogeny within Chelicerata [38–40], the relationship within Lophotrochozoa [13, 41–43] or the relationship between Placozoa, Porifera, Cnidaria and Ctenopohora within the Metazoa [44–48] are remarkably sensitive to methods of analyses. Recent simulation studies show that even a slight model misspecification, such as that arising from approximating among site rate heterogeneity using discrete categories, can cause incorrect topologies in maximum likelihood (ML) analyses [12, 49].

A major source of systematic bias, and probably the most frequently cited reason for incorrect placements of taxa in phylogenetic reconstructions, is long branch attraction (LBA). First described by [50] as a problem of parsimony and compatibility methods, later studies revealed that even more robust, probabilistic tree reconstruction methods such as ML and Bayesian inference (BI) can fail to find the correct tree because of LBA, e.g. [8, 21, 23, 32, 41, 49, 51–63].

LBA is commonly understood as an incorrect phylogenetic reconstruction of two or more highly-divergent (long branch) lineages as sister (rooted) or adjacent (unrooted) groups due to the accumulation of convergent split signal (chance similarities) and the simultaneous loss of apomorphic characters shared with the actual close relatives, e.g. [32, 50, 61, 64]). [12] have shown that the probability of incorrect phylogenetic inferences increases with increasing heterogeneity of only inner edges and that unbalanced length differences between internal and terminal branches can have a negative effect on the tree reconstruction process when internal branch lengths are either too short or too long. Our usage of the term LBA is equivalent to the characterisations of [23] and [32]: “…conditions under which bias in finite data set analyses and/or statistical inconsistency arise due to the combination of short and long branches”.

Different strategies exist for ameliorating LBA in phylogenetic analyses. Possibilities include the analysis of only slowly evolving sequences to reduce branch lengths [65] or the addition of slowly-evolving taxa to divide long internal branches [66]. However, slowly evolving sequences are sometimes not available, not least because of extinction [32], and exclusion of rapidly-evolving taxa reduces taxon sampling, which is often considered undesirable [67–74]. The exclusion of complete long-branched groups might successfully reduce LBA, but is not helpful if the relationship of those taxa is of importance to the study in question. Another frequently used strategy is the removal of sequence positions inferred to be fast evolving, e.g. [75–78], or entire classes of putatively fast evolving sites such as third codon positions in protein coding nucleotide data sets, which are potentially saturated by multiple substitutions [53, 79]. Conversion of nucleotides to more slowly evolving character states such as amino acid residues or purines and pyrimidines [60] is another strategy. One likely reason for misspecifications in modern probabilistic substitution models is the usual assumption of time reversibility. The direction of character evolution along a tree is not considered by these models and therefore these analyses do not incorporate an important step of Hennigian phylogenetic inference, the distinction between new (apomorphic) and old (plesiomorphic) homologies [49].

The susceptibility of ML to systematic biases in cases where these is model misspecification motivates us to ask: is it possible to develop alternative techniques that are less effected than is ML by, for example, extreme branch length asymmetries? Here we introduce *PhyQuart*, a new, quartet-based algorithm which considers two alternative directions of character evolution along the internal branch of a quartet tree to discern between potentially apomorphic and plesiomorphic split-supporting site-patterns, and ML to estimate the expected number of convergent split-supporting site-patterns. This combination of Hennigian logic and ML estimation represents a completely new strategy for the evaluation of sequence data. A quartet tree comprising one internal and four external branches is the smallest phylogenetically informative unrooted tree. It is sometimes helpful to focus on quartets because of their computational simplicity: there are only three alternative topologies to be investigated and far fewer potential site-patterns (the basic empirical data from which inferences are to be made) than in alignments containing many taxa. Despite this helpful computational simplicity, it is widely believed that quartet analyses exacerbates LBA (because it is the opposite of adding taxa so as to break up long branches) and thus represents the most difficult taxon sampling context in which to overcome LBA [80]. Through extensive quartet simulations, including cases with strong branch length differences, we demonstrate the efficiency of our new approach in detecting phylogenetically informative and conflicting signals and compare its performance to ML alone when there is a (unrealistically) small degree of model misspecification. The *PhyQuart* algorithm is implemented in a command line driven software script.

## 1 The method: Concept and algorithm

### 1.1 Concept

The *PhyQuart* algorithm takes as input an alignment and outputs normalised split-support for alternative quartet trees based on a site-pattern classification and using observed and expected (based on ML inference) frequencies of split-supporting site-patterns, considering the Hennigian distinction between phylogenetically informative (apomorphic) and uninformative (plesiomorphic) character.

Here we define some basic concepts and provide a brief overview of our approach. A more complete and formal description of the *PhyQuart* algorithm is given in the next section.

We decided to use the established terms plesiomorphy and apomorphy to distinguish between old (pelsiomorphic) and new (apomorphic) shared homologous character states. The alternative would have been to invent some new term for the same meaning, which clearly is not a better option. Both terms describe a simple fact that is observed everywhere where evolution takes place: an old state is modified and transformed to a new state. Or, something that did not exist appears de novo. This is not different from saying that there is a sequence (a complex character) in which a nucleotide at a specific site position of an alignment is substituted by a new nucleotide, which would be the apomorphic detail. The discovery that it makes a difference whether in molecular evolutionary processes the polarity in time is considered or not has recently been published by Kück & Wägele [49]. Phylogenetically informative split-supporting site-patterns are only those site-patterns which contain apomorphies. Further, we define an informative split-supporting sitre-pattern as “putative synapomorphy” when a shared apomorphic character similarity between two taxa on one side of a split is assumed to be present in the most recent common ancestor (internal node of a tree).

The goal of the *PhyQuart* algorithm is to identify among all split-supporting site-patterns those that support polarized splits with characters that are probably putative synapomorphiies.

**Fig 1:**
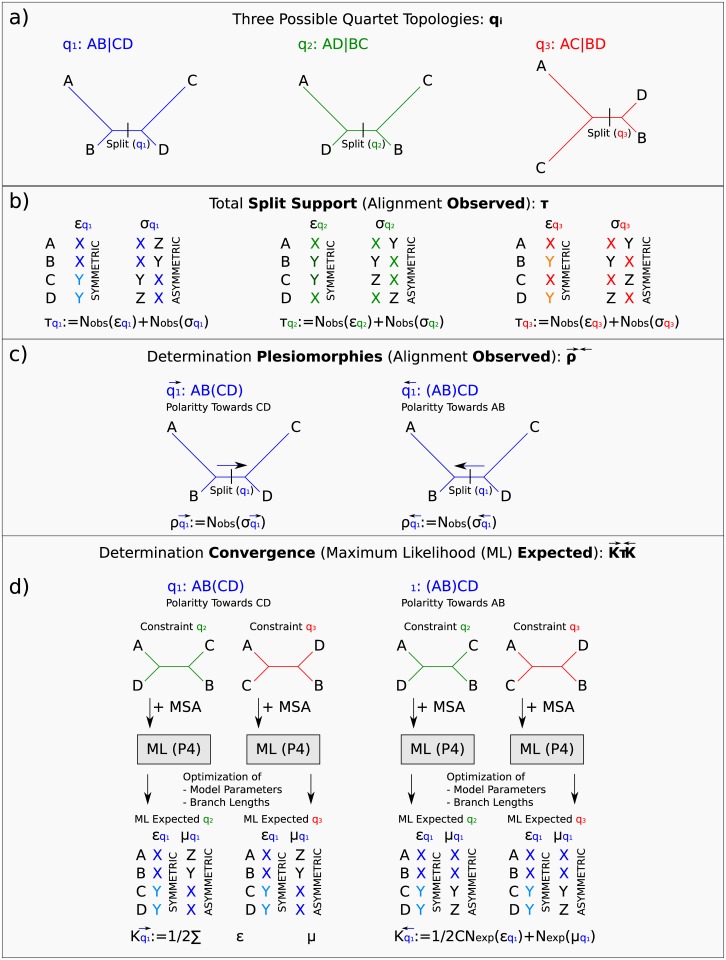
Flowchart of the *PhyQuart* algorithm. Simplified flowchart showing a) each of the three possible quartet relationships for a set of 4 sequences (*q*_1_, *q*_2_, *q*_3_), b) the site-pattern classification of observed (*N*_*obs*_) symmetric ($\xi_{q_{i}}$) and asymmetric ($\sigma_{q_{i}}$) support ($\tau_{q_{i}}$), c) the determination of plesiomorphic (old) split-supporting site-patterns given two different polarities of character transformation along the internal branch of each possible quartet tree, $\rho_{\vec{q_{1}}}$ and $\rho_{\overset{\leftarrow }{q_{1}}}$, and d) estimation of expected convergent split-supporting site-patterns ($\kappa_{\vec{q_{1}}},\kappa_{\overset{\leftarrow }{q_{1}}}$) supporting quartet *q*_1_ in ML split pattern estimations using branch length and model optimization on constraint topologies of the other two possible quartet relationships (*q*_2_, *q*_3_).

A split is a bipartition of a set of species or sequences [62, 64, 81]. A sequence position supports some split if no pair of taxa separated by split share the same character state [82]. For the quartet of taxa A-D there are three phylogenetically informative quartet splits AB|CD, AC|BD and AD|BC, corresponding to the single internal branches of the three possible unrooted binary quartet trees. Let W-Z correspond to different character states (e.g., nucleotides or amino acid residues). Sites with the character distribution {WXYZ} support all three quartet trees and thus do not differentially support any of them, whereas a sequence position differentially supports one quartet split/tree if two taxa have the same character state and the other two taxa have some other character state(s). Thus, sites with the character state distribution {XXYY} (symmetric) or {XXYZ} and {YZXX} (two possible asymmetric) split-supporting site-patterns are counted as differential split-support for the quartet tree AB|CD (Fig 1a and 1b).

We denote the polarity of (i.e., the direction of character transformation along) the internal branch of a given quartet tree AB|CD using parentheses, e.g. AB(CD) and (AB)CD which indicate the direction is towards CD and towards AB respectively. This distinction enables a classification of split-supporting site-patterns into potentially phylogenetic informative (apomorphic) and uninformative (plesiomorphic) site-patterns [83] contingent on the assumed polarity. Thus, the asymmetric split-supporting site-pattern {XXYZ} is interpreted as supporting the polarized quartet tree (AB)CD because the shared character state similarity of A and B appears apomorphic but as uninfomative for the polarized quartet tree AB(CD) because the similarity of A and B appears plesiomorphic (Fig 1c). Because the *PhyQuart* algorithm assumes no *a priori* knowledge of polarity (placement of the root) each possible polarity for each of the three quartet trees is evaluated separately for each quartet of taxa (for a total of six evaluations) to find the best supported quartet tree based exclusively on putative synapomorphic character states.

Putative synapomorphy can be phylogenetically misleading if the similarity is not homologous but rather evolved convergently. For example, given a polarized quartet tree (AB)CD, similarity would be present in split-supporting site-patterns with characters shared between taxon C and D: {XXYY} and {XZYY}. However, observed split-supporting site-patterns for (AB)CD can be either synapomorphic (inherited from a common ancestor of C and D) or convergently evolved along the terminal branches of C and D. Suppose that given polarized quartet trees (AB)CD and AB(CD) of quartet tree *q1* are incorrect and thus the correct quartet tree is one of the two alternative topologies (*q2* or *q3*). *PhyQuart* uses ML to estimate how much apomorphic support for each polarization (number of split-supporting site-patterns {XXYY} and {XZYY} for (AB)CD and {XXYY} and {XXYZ} for AB(CD)) would have evolved with the branch lengths of the underlying data if *q2* were the correct tree, or if *q3* were the correct tree. These values are equivalent to the number of parallel substitutions on unrelated branches that are expected if *q1* is the correct tree. We take the mean of these two values as the estimate of expected convergently-evolved, misleading support for each polarized quartet tree of *q1* (Fig 1d). The ML inference of expected convergence for each polarized quartet tree is implemented using the P4 package [84] with individually optimised branch length and model parameters for each of the alternative quartet trees. The estimated number of misleading sites is then subtracted from the observed number of supporting positions to get an estimate of the synapomorphic support for each polarized quartet tree.

Multiple substitutions among longer branches may lead to underestimation of the support for the correct quartet tree. To reduce the effects of such underestimation we use correction factors (*ω*) based on the frequencies of the four singleton patterns (i.e. {XXXY}, {XXYX}, {XYXX}, {YXXX}) that are intended to make the corrected support values closer to what would be expected if external branches were of equal length. Correction factors are applied to both observed (*ω*_*obs*_) and ML inferred expected (*ω*_*exp*_) frequencies of site-patterns. In each case, the correction factor reduces the split-supporting site-patterns in proportion to the complement of four times the frequency of the least frequent singleton pattern (observed or expected) divided by the sum of the frequencies of all four singleton patterns. The frequencies of observed singleton site-patterns and the corresponding correction factor (*ω*_*obs*_) are constant for all quartet trees and polarities, whereas *ω*_*exp*_ can differ for ML expected frequencies. Thus by itself *ω*_*obs*_ has no effect on relative support for different quartet trees, rather it is *ω*_*exp*_ that drives the effect of the correction. When there are strong branch length asymmetries *ω*_*exp*_ and thus the corrected estimate of convergent split-supporting site-patterns will be high. Thus, the correction is important in cases of unequal branch lengths (as evidenced by differences in singleton frequencies) such as can produce LBA.

Let *P* be a polar quartet tree. Let *S*_*obs*_ be the sum of the observed numbers of symmetric and asymmetric site-patterns supporting *P* and let *M*_*obs*_ be the smallest number (the minimum) taken over all four singletons, and *T*_*obs*_ be the total number of observed singleton site-patterns. Let *ω*_*obs*_ = 1 − (4*M*_*obs*_/*T*_*obs*_). Similarly, using ML estimation for the two contrary quartet trees (that conflict with *P*), let *S*_*exp*1_ and *S*_*exp*2_ each be the sum of the expected number of symmetric and asymmetric site-patterns supporting *P* and let *M*_*exp*1_ and *M*_*exp*2_ be the smallest numbers and let *T*_*exp*1_ and *T*_*exp*2_ be the total numbers respectively of expected singleton site-patterns. Let *S*_*exp*_ = (*S*_*exp*1_ + *S*_*exp*2_)/2 and let *ω*_*exp*_ = 1 − (4*M*_*exp*1_/*T*_*exp*1_ + 4*M*_*exp*2_/*T*_*exp*2_)/2. Then the *PhyQuart* score for any *P* is (*S*_*obs*_ − (*S*_*obs*_ * *ω*_*obs*_)) − (*S*_*exp*_ − (*S*_*exp*_ * *ω*_*exp*_)) and the score for each quartet is the highest of the scores for its polarized quartets normalised so that the scores for all three alternative quartets sum to one.

### 1.2 Algorithm

#### 1.2.1 Observed split support of each quartet tree

First, the algorithm counts the total number of observed split-supporting site-patterns (*τ*) in a given set of four aligned sequences of length *L* for each of the three possible quartet topologies *x* ∈ *Q* ≔ {*q*_1_, *q*_2_, *q*_3_}. All site-patterns (*s*) with symmetric (*ξ*) and asymmetric (*σ*) split-support for a given quartet relationship are taken into account (Fig 1a and 1b).

$$Q≔\{q_{1},q_{2},q_{3}\}$$ (1)

$$\begin{array}{c} \tau_{x}≔\sum_{i=1}^{L}1_{\{s_{i}\in \left(\xi_{x}\vee \sigma_{x}\right)\},x\in Q} \end{array}$$ (2)

#### 1.2.2 Determination of plesiomorphic split signal

To identify potentially plesiomorphic split-supporting site-patterns of a given quartet tree (*x* ∈ *Q*), two different polarities are specified: $Q_{polar}≔\left\{\vec{x},\overset{\leftarrow }{x}\right\}$. Each polarity defines one of the two possible directions of character transformation along the internal branch of a given quartet tree. Quartet-supporting positions based on symplesimorphic split-supporting site-patterns are counted separately for each polarized quartet tree *z* ∈ *Q*_*polar*_.

The right pointing direction of a quartet tree ($z=\vec{x}$) defines asymmetric *z* split-supporting site-patterns (*σ*_*z*_) as apomorphic ($\varrho_{z}$) whenever identical character states are only shared between taxa A and B in quartet tree *z*≔ AB(CD).

For example, site-pattern *s* ≔ {*XXYZ*} contains asymmetric split-supporting site-patterns (*s* = *σ*_*z*_) based on a plesiomorphic character state (*σ*_*z*_ = *ϱ*_*z*_) if polarity *z*≔ AB(CD). Otherwise, with the left pointing direction of a quartet tree ($z=\overset{\leftarrow }{x}$, *z*≔ (AB)CD, site-pattern *s* = *σ*_*z*_, but *σ*_*z*_ ≠ *ϱ*_*z*_, and the site-pattern is interpreted as apomorphic.

The total number of observed *ϱ*_*z*_ sites of a split-supporting site-pattern for a given polarized quartet relationship *z* (given a sequence length *L*) is defined as *ρ*_*z*_ (Fig 1c).

$$Q_{polar}≔\left\{\vec{q_{1}},\overset{\leftarrow }{q_{1}},\vec{q_{2}},\overset{\leftarrow }{q_{2}},\vec{q_{3}},\overset{\leftarrow }{q_{3}}\right\}$$ (3)

$$\begin{array}{c} \rho_{z}≔\sum_{i=1}^{L}1_{\left\{s_{i}\in \varrho_{z}\right\},z\in Q_{polar}} \end{array}$$ (4)

#### 1.2.3 Determination of convergent split signal

Contrary to the identification of plesiomorphic split-supporting site-patterns observed in a given alignment of sequence length L, the total amount of potentially convergent split-supporting site-patterns (*κ*_*z*_) for a given polarity *z* ($z\in Q_{polar}≔\left\{\vec{q_{1}},\overset{\leftarrow }{q_{1}},\vec{q_{2}},\overset{\leftarrow }{q_{2}},\vec{q_{3}},\overset{\leftarrow }{q_{3}}\right\}$) of a quartet tree *x* (*x* ∈ *Q* ≔ {*q*_1_, *q*_2_, *q*_3_}) is determined by ML estimation of symmetric ($\xi_{exp_{\pi (z)}}$) and asymmetric ($\sigma_{exp_{z}}$) split-supporting site-pattern frequencies, which support tree *x* based on constraint topologies of the other two possible quartet relationships *y* (*y* ∈ *Q* \ {*π*(*z*)}). Thereby, *π* is defined as the projection of *Q*_*polar*_ onto *Q* (*π*: *Q*_*polar*_ → *Q*), saying that $\pi \left(\vec{q_{i}}\right)=q_{i}=\pi \left(\overset{\leftarrow }{q_{i}}\right)$ for *i* = 1, 2, 3. Note that polarity is not relevant for ML inferences, but ML estimated site frequencies depend on branch lengths.

Site-pattern frequencies of each constrained topology *y* are calculated by ML using branch length and model parameter optimization on the basis of the original quartet alignment and a defined substitution model. Estimated frequencies of each possible site-pattern are multiplied by the original alignment length *L* to get the expected number of sites for a pattern in a given alignment.

For each polarity of a given quartet *z* (*z* ∈ *Q*_*polar*_), the ML expected number of the potentially convergent split-supporting site-patterns (chance similarities) (*κ*_*z*_) is defined by the mean number of ML estimated split symmetric ($\xi_{exp_{\pi (z)}}$) and asymmetric site-patterns ($\sigma_{exp_{z}}$), supporting polarized tree *z*.

Given tree z, the expected number of chance similarities (e.g. for (CD)) is estimated with the number of characters (*μ*_*z*_) shared by C and D in the two other quartet-topologies *y* (*y* ∈ *Q* \ {*π*(*z*)}), where they are not adjacent and thus cannot be sister-taxa. We use for each split-group the average (*κ*_*z*_) of these two values (Fig 1d).

For example, the asymmetric split-supporting site-pattern *σ*_*z*_ ≔ {*YZXX*)} shares identical character states only between taxa C and D in *z* ≔ {*AB*(*CD*)}, therefore: *σ*_*z*_ = *μ*_*z*_. Otherwise, if *σ*_*z*_ ≔ {*XXYZ*)}, then: *σ*_*z*_ ≠ *μ*_*z*_.

$$\begin{array}{c} \kappa_{z}≔\frac{1}{2}\sum_{y\in Q\setminus \{\pi (z)\}}\left(\xi_{exp_{\pi (z)}}^{y}+\mu_{z}^{y}\right),z\in Q_{polar} \end{array}$$ (5)

#### 1.2.4 Further noise reduction using correction factor *ω*

Singleton site-pattern frequencies can be used as an approximation for terminal branch lengths. Fast evolving sequences will have more of these than slower ones. Four different singleton site-patterns are possible, {YXXX}, {XYXX}, {XXXY}, and {XXYX}, each of them contributing to one of the four terminal branch lengths.

To further reduce the impact of noise upon the identified number of split-supporting site-patterns for a given polarity *z* ($z\in Q_{polar}≔\left\{\vec{q_{1}},\overset{\leftarrow }{q_{1}},\vec{q_{2}},\overset{\leftarrow }{q_{2}},\vec{q_{3}},\overset{\leftarrow }{q_{3}}\right\}$) of a quartet tree *x* (*x* ∈ *Q* ≔ {*q*_1_, *q*_2_, *q*_3_}), the algorithm reduces for each polarity (*z*) of a given tree (*x*) the total number (*τ*_*x*_) of counted symmetric (*ξ*_*x*_) and asymmetric (*σ*_*x*_) split-supporting site-patterns as well as the number of plesiomorphic (*ρ*_*z*_) and convergent split-supporting site-patterns (*κ*_*z*_).

The correction factor (*ω*) is defined as one minus the ratio of four times the smallest number of the singleton site-patterns (*ϕ*) to the total number of singleton site-patterns (*N*). The total number of tree *x* supporting split signal (*τ*_*x*_) as well as the the number of plesiomorphic split-supporting site-patterns (*ρ*_*z*_) for a given polarity *z* of tree *x* (*z* ∈ *Q*_*polar*_) are reduced in relation to single substituted site-pattern frequencies of the original quartet alignment (*ω*_*obs*_).

$$\begin{array}{c} \omega_{obs}≔1-\left(\frac{4\phi }{N}\right) \end{array}$$ (6)

The correction factor ($\omega_{exp_{z}}$) for convergent split-supporting site-patterns (*κ*_*z*_) of a given polar quartet tree (*z*) is specified by the mean of the two single correction factors (*ω*_*z*_), which are derived (in the same manner as described in Eq 7) from the ML-estimated singleton site-pattern frequencies of the other two quartet topologies *y* (*y* ∈ *Q* \ {*π*(*z*)}).

$$\begin{array}{c} \omega_{exp_{z}}≔\frac{1}{2}\sum_{y\in Q\setminus \{\pi (z)\}}\omega_{z}^{y},z\in Q_{polar} \end{array}$$ (7)

#### 1.2.5 Determination of potential apomorphic split signal (*θ*)

Only the actual number of potentially synapomorphic, split-supporting site-patterns is counted as phylogenetic signal. To identify the number of potentially synapomorphic split-supporting site-patterns for each possible polarized quartet tree (*z*), the total number of observed split-supporting site-patterns (*τ*_*z*_) as well as the number of potentially plesiomorphic (*ρ*_*z*_) and convergent (*κ*_*z*_) split-supporting site-patterns are adjusted by the correction factor *ω*_*obs*_ and $\omega_{exp_{z}}$. Afterwards, the remaining (synapomorphic) split signal is calculated for each polar quartet tree by subtracting the corrected phylogenetic uninformative plesiomorphic and convergent split-supporting site-patterns from the corrected number of observed split-supporting site-patterns.

$$\theta_{z}≔\left(\tau_{\Pi (z)}*\omega_{obs}\right)-\left(\rho_{z}*\omega_{obs}\right)-\left(\kappa_{z}*\omega_{exp_{\pi (z)}}\right),z\in Q_{polar}$$ (8)

#### 1.2.6 Final quartet weighting (λ) for polarized topologies based on best polar quartet tree support values

After the assignment of the actual, potentially synapomorphic split supporting site-patterns all three quartet topologies *x* (*x* ∈ *Q* ≔ {*q*_1_, *q*_2_, *q*_3_}) are scored (*δ*_*x*_) related to their higher number of split-supporting site-patterns given both possible polarities ($\theta_{a},\theta_{b}\in Q_{polar}≔\left\{\vec{x},\overset{\leftarrow }{x}\right\},x\in Q$). For example, if the obtained score of polarity *θ*_(*AB*)*CD*_ > *θ*_*AB*(*CD*)_, then *δ*_*AB*|*CD*_ ≔ *θ*_(*A*, *B*)*CD*_.

$$\begin{array}{c} \delta_{x}≔\{\begin{array}{cc} \theta_{a} & \text{if}\;\theta_{a}\geq \theta_{b}\\ \theta_{b} & \text{if}\;\theta_{a}<\theta_{b} \end{array},\left(\theta_{a},\theta_{b}\in Q_{polar}≔\left\{\vec{x},\overset{\leftarrow }{x}\right\},x\in Q\right) \end{array}$$ (9)

Finally, each quartet tree *x* (*x* ∈ *Q* ≔ {*q*_1_, *q*_2_, *q*_3_}) is weighted (λ_*x*_) equal the difference between the actual number of split-supporting site-patterns (*δ*_*x*_) and the lowest number of split-supporting site-patterns given all three quartets ($\delta_{lowest_{x}}$), normalised by the sum of single quartet weights. For example, if $\delta_{lowest_{x}}=\delta_{AC|BD}$, then λ_*AB*|*CD*_ ≔ *δ*_*AB*|*CD*_ − *δ*_*AC*|*BD*_.

$$\begin{array}{c} \lambda_{x}≔\frac{\delta_{x}\setminus \delta_{lowest_{x}}}{\sum_{i\in Q}\lambda_{i}},x\in Q \end{array}$$ (10)

### 1.3 Software implementation

The algorithm introduced in this study is implemented in a new software tool called PENGUIN, a command line driven PERL script that runs on Windows PCs, Mac OS and Linux operating systems and can be easily implemented into automatic process pipelines. A PERL interpreter must be present in order to execute the software. PENGUIN is freely available (i.e., open-source) and released under the terms of the GNU General Public License (GPL) 3.0. The software script as well as the corresponding manual and example files can be downloaded from https://github.com/PatrickKueck/Penguin.

PENGUIN reads files of multiple sequence alignments in FASTA and PHYLIP format. If the alignment consists of more than four sequences, a clan input file comprising four predefined clans (sensu [85]) of one or more taxa must be provided in plain TEXT format. If specified, PENGUIN analyses all possible quartet combinations of one taxon from each predefined clan. PENGUIN does not allow multiple records of the same taxon name within given input file(s) and mismatches between taxa included in a predefined clan file and a multiple sequence alignment are just left unanalysed. The script can handle both nucleotides and amino-acid sequences. Sequence sites with indels (gap or ‘-’), ambiguity or missing characters are always excluded from the analysis. Under default, PENGUIN excludes all forbidden site positions separately for each quartet of sequences drawn from a given multiple sequence alignment. This has the advantage that sequence positions do not have to be deleted from the full alignment and can be used in cases of other quartets that do not have such ambiguities in these positions. Alternatively, site exclusion can be performed on the complete sequence alignment in advance of the quartet establishment. However, the performance of our algorithm has only been tested on nucleotide data without simulated indels, ambiguities, or missing data.

PENGUIN writes information on split support for each possible quartet relationship between four taxa or clans in plain TEXT files. Obtained discrepancies in topological split support of the three possible quartet topologies of a set of four clans are also presented as split network and triangle graphs. A further vector network shows the distribution of best, second best, and third best resolved quartet trees.

Detailed information about single analysis output is provided by the PENGUIN script manual.

## 2 Performance

**Fig 2:**
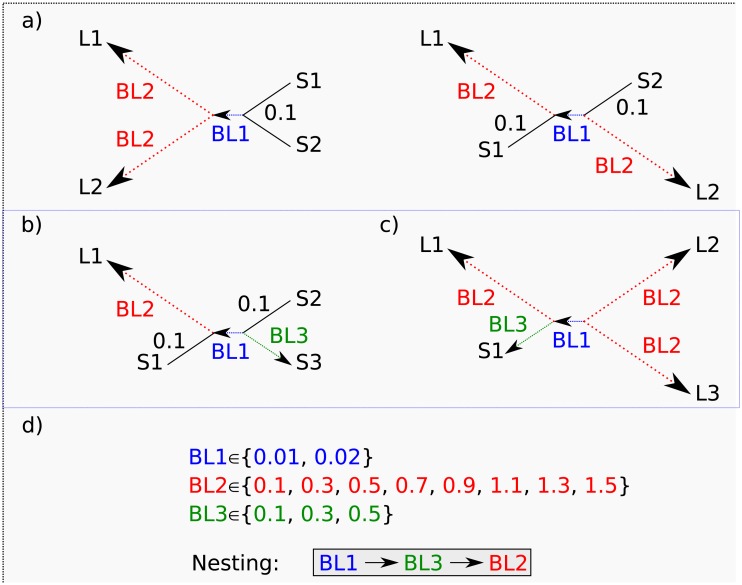
Quartet simulation setups. Simulation setups for quartet analyses testing effects of given a very short internal branch (BL1) with a) stepwise elongation of two adjacent (Farris-topology, left) and non-adjacent (Felsenstein-topology, right) terminal long branches (BL2), b) elongation of one terminal branch (BL2) using different lengths for one of the three short branches (BL3), and c) stepwise elongation of three terminal branches, with different lengths of the remaining short terminal branch (BL3).

The *PhyQuart* algorithm was tested and its performance compared with ML using 172,800 simulations. Varying amounts of nucleotide sequence data was simulated using INDELible v.1.03 [86] on quartet trees with different combinations of fixed (at 0.1), alternative internal (BL1 = {0.01, 0.02}), and more (BL2) or less highly varied (BL3) terminal branch lengths (Fig 2) under the GTR model of sequence evolution. Among site rate variation (ASRV) was modelled using a continuous Γ-rate distribution with different shape parameters and a fixed proportion of invariant sites (0.3). Simulations did not include indels. Table 1 summarises the parameters employed in the analyses. ML trees were inferred from simulated data with PhyML_3.0_linux64 [87, 88], using a mixed-distribution model (GTR+Γ+I) with the model parameters (*α*, I) used in the simulation but with the simulated continuous gamma distribution approximated by a discrete gamma with four relative substitution rate categories and the relative rates and base compositions estimated from the data. This difference in gamma (continuous or discrete) and any small differences in the simulated and estimated relative rates and base compositions are the only model misspecifications involved in the ML inference. Thus we expect ML to perform well in most cases. The ML estimation of split site-pattern frequencies in the *PhyQuart* algorithm used the same model but with all parameters estimated from the underlying data. All analyses were performed and evaluated with a Perl pipeline. We generated 100 multiple sequence alignments for each combination of internal and terminal branch lengths and recorded the frequencies of correct and incorrect tree reconstructions from these replicates.

**Table 1:** Defined model parameters for data simulation (INDELible) and ML analyses used in *PhyQuart* and PhyML.

Simulation Setup	Seq. Length	I	Γ (*α* Shape Parameter)					
1-elongated branch	(BL2) = 250 kbp	0.3	0.1	0.3	0.5	0.7	1.0	2.0
2-elongated branches	(BL2) = 250 kbp	0.3	0.1	0.3	0.5	0.7	1.0	2.0
2-elongated branches	(BL2) < 250 kbp	0.3			0.5		1.0	2.0
3-elongated branches	(BL2) = 250 kbp	0.3	0.1	0.3	0.5	0.7	1.0	2.0
**GTR Substitution Rates**								
INDELible:	C↔T: 0.3; T↔A: 0.8; T↔G: 0.6; C↔A: 0.5; G↔C: 0.4; G↔A: 1.0							
*PhyQuart*:	Estimated							
PhyML:	Estimated							
**Nucleotide Frequencies**								
INDELible:	T: 0.35; C: 0.15; A: 0.35; G: 0.15							
*PhyQuart*:	Estimated							
PhyML:	Estimated							

### 2.1 Elongation of two terminal branches

Our first quartet simulations test the classical quartet LBA problem [50] with two alternative topologies in which two terminal long branches were either adjacent (termed “Farris” topologies), or non-adjacent (termed “Felsenstein” topologies) (Fig 2a). We examined the stepwise elongation of the long terminal branches (BL2 = {0.1 → 1.5} in steps of 0.2) with the other two terminal short branches kept constant (length = 0.1) and two alternative internal branch lengths (BL1 = {0.01, 0.02}) analysing a wide range of sequence lengths (0.5, 1, 2, 5, 10, 20, 50, 100, 250 thousands of base pairs (kbp)). For data sets of 250 kbp, we simulated sequences with six different rate heterogeneity parameters (*α* = {0.1, 0.3, 0.5, 0.7, 1.0, 2.0}) whereas for shorter sequences we analysed three different heterogeneties (*α* = {0.5, 1.0, 2.0}).

**Fig 3:**
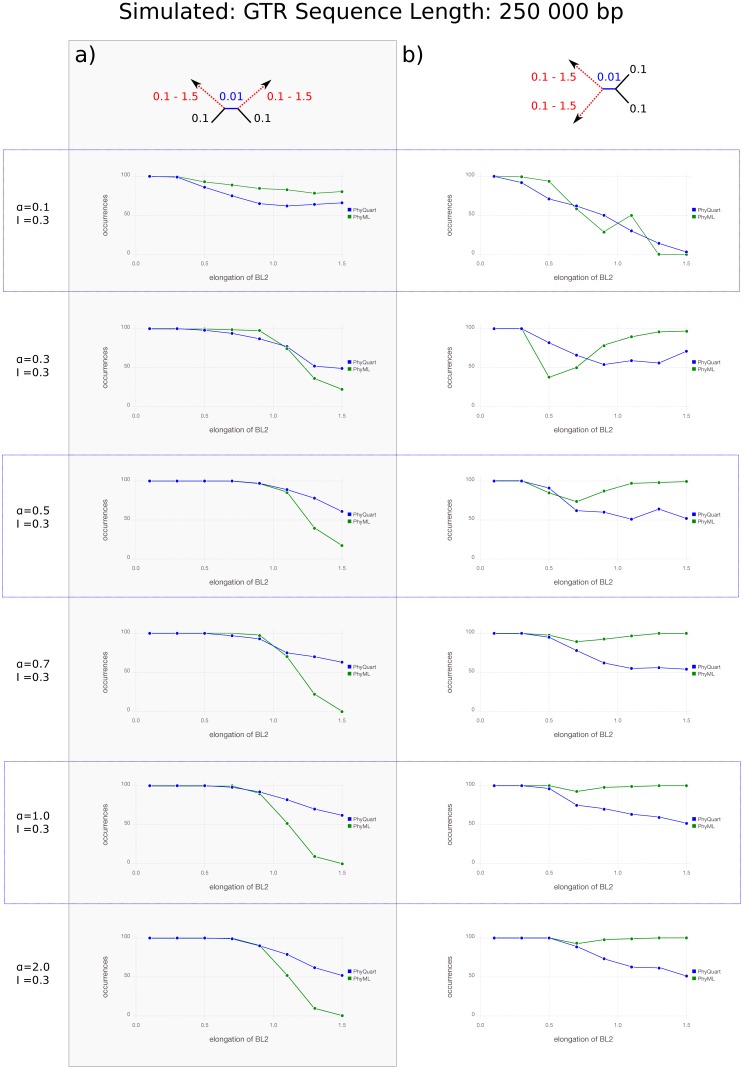
Quartet reconstruction success given stepwise elongation of two terminal branches if sequence lengths equal 250 kbp. Plots visualizing the reconstruction success of *PhyQuart* (blue) and ML (green) given stepwise elongation of two terminal branches (BL2, x-axis) and a fixed, very short internal branch length (BL1 = {0.01}) for 100 (250 kbp long) data replicates (y-axis). The plots present the summarized reconstruction success for (a) Felsenstein-, and (b) Farris-topologies of given *α* = {0.1, 0.3, 0.5, 0.7, 1.0, 2.0} and an invariable site proportion (I) of 0.3. A detailed overview of all simulation results of this setup is given as supplementary information S1 Fig.

With the longest simulated sequences (250 kbp), ML mostly performs very well in reconstructing Farris topologies, but as the ratio of long to short branches increases reconstruction success for Felsenstein topologies decreases precipitously (Fig 3). In contrast, *PhyQuart* successfully reconstructs Felsenstein topologies in the majority of replicates, independent of simulated model parameter and branch length conditions and, except for strongly heterogeneous data sets (*α* = 0.1), *PhyQuart* outperforms ML especially with the shortest internal branches (BL1 = {0.01}) (Fig 3a). While not as successful as ML in reconstructing simulated Farris topologies, *PhyQuart* successfully reconstructs these in a majority of cases when *α* > 0.1, while both reconstruction methods often failed in cases of high branch length heterogeneity for data sets simulated with *α* = 0.1 (Fig 3b). Except for very strong heterogeneous data simulations (*α* = 0.1), ML outperformed *PhyQuart* in identifying correct Farris topologies if terminal branches exceeded a length 70 times higher as the internal branch (BL2 ≥ {0.7}). Contrary to ML, the *PhyQuart* algorithm consistently recovered correct Farris and Felsenstein topologies in the majority of the (250 kbp long) replicates, even in simulations with very low internal branch signal of the correct tree (BL1 = {0.01}) if *α* > 0.1 (Fig 3). Reconstruction successes for all Felsenstein and Farris topology simulations based on sequence lengths of 250 kbp are given in the supplementary file S1 Fig. Comparison of the *PhyQuart* reconstruction results of this setup with and without implementation of the correction factor *ω* are given in the supplementary information S2 Fig.

**Fig 4:**
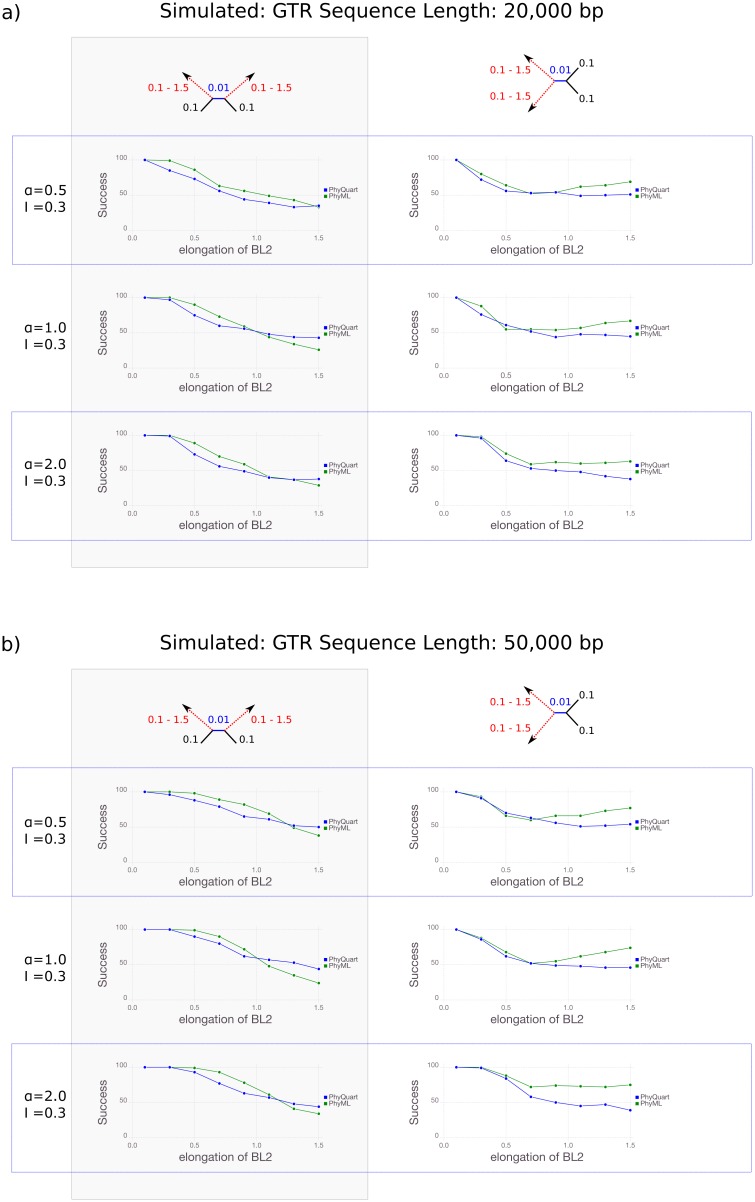
Quartet reconstruction success given stepwise elongation of two terminal branches if sequence lengths < 250 kbp. Reconstruction success of *PhyQuart* (blue) and ML (green) for different rate heterogeneities and for Farris as well as Felsenstein topologies under different lengths of two elongated terminal branches (BL2, x-axis), given a fixed internal branch length (BL1 = {0.01}), and 100 data replicates (y-axis). Reconstruction success for data sets of sequences <250 kbp are summarized for *α* = 0.5, 1.0, and 2.0: a) 20 kbp, b) 50 kbp. A detailed overview of all simulation results of this setup is given as supplementary information S3 Fig.

Reconstruction success decreases with sequence length when branch lengths are heterogeneous. ML and *PhyQuart* correctly recovered Felsenstein and Farris topologies in the majority of data replicates given a wide range of internal and terminal branch conditions if sequence length exceeds 50 kbp (Fig 4). Considering the reconstruction success for different rate heterogeneities and for Farris as well as Felsenstein topologies, ML slightly outperforms *PhyQuart* in cases of strong branch length differences if sequence are shorter than 50 kbp for longer internal branch lengths (BL1 = {0.02}) whereas *PhyQuart* outperfoms ML with the shorter internal branch (BL1 = {0.01}). *PhyQuart* often outperforms ML if sequences are longer than 50 kbp (Fig 4). Detailed summaries of our analyses with sequence lengths shorter than 250 kbp are given in the supplementary information S3 Fig.

### 2.2 Elongation of one terminal branch

**Fig 5:**
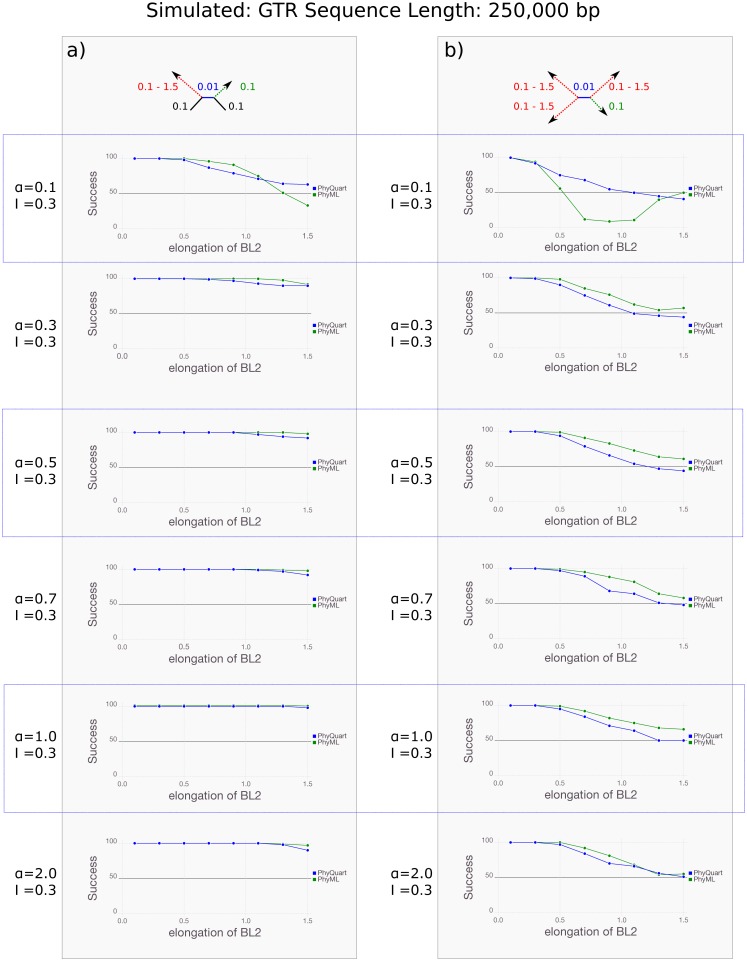
Quartet reconstruction success given stepwise elongation of one or three terminal branches if sequence lengths equal 250 kbp. Reconstruction success of *PhyQuart* (blue) and ML (green) for different rate heterogeneities under different lengths of a) a single long terminal branch (BL2, x-axis) and b) three long terminal branches (BL2, x-axis), given 100 data replicates (y-axis) of 250 kbp length and a fixed alternative internal branch length of BL1 = {0.01}, summarized for *α* = {0.1, 0.3, 0.5, 0.7, 1.0, 2.0}. A detailed overview of all simulation results of both setups is given as supplementary information S4 and S5 Figs.

Our second quartet simulation experiments (Fig 2b) investigate reconstruction success when there is one long and three short terminal branches. These experiments also used two alternative internal branch lengths of BL1 = {0.01, 0.02}, stepwise elongation of the single long terminal branch (BL2 = {0.1 → 1.5} in steps of 0.2) with two of the remaining terminal branches kept constantly short (= 0.1) and the third branch also stepwise elongated (BL3 = {0.1, 0.3, 0.5}). Sequence lengths were 250 kbp with six alternative rate heterogeneities (*α* = {0.1, 0.3, 0.5, 0.7, 1.0, 2.0}). Both *PhyQuart* and ML performed well in all analyses independent of simulation parameters with sometimes slightly better performance of ML and vice versa. Fig 5a shows the similar reconstruction success of both methods given three equal short terminal branches. Detailed result plots of all “single long branch” simulation analyses are given in the supplementary information S4 Fig.

### 2.3 Elongation of three terminal branches

Our third quartet simulation experiments (Fig 2c) involve stepwise elongation of three terminal branches (BL2 = {0.1 → 1.5} in steps of 0.2) with a stepwise increase of the fourth terminal branch (BL3 = {0.1, 0.3, 0.5}), two alternative internal branch lengths (BL1 = {0.01, 0.02}), six rate heterogeneities (*α* = {0.1, 0.3, 0.5, 0.7, 1.0, 2.0}) and sequences of 250 kbp. As with other experiments, success decreases with increasing length differences between internal and terminal branches for both methods (Fig 5b). With the exception of data simulated with high among site rate variation (*α* = {0.1}), ML typically slightly outperformed *PhyQuart*. Detailed result plots of all performed analyses for cases with three long branches are given in the supplementary information. Reconstruction success of both methods was not strongly uninfluenced by the length of the shorter fourth terminal branch (BL3), but the shorter this branch (BL3) and the longer the internal branch length (BL1), the better the performance of both methods given three strongly elongated terminal branches. Detailed result plots of all “three long branch” simulation analyses are given in the supplementary information S5 Fig.

## 3 Discussion

Not without good reasons, modern molecular phylogenetics is dominated by the probabilistic, model-based ML and Bayesian methods. However, although these approaches have much to recommend them, they can fail to recover the correct tree and may instead recover the wrong tree with misleading high support when available models do not adequately represent underlying evolutionary dynamics. The robustness of ML to variation in evolutionary processes and the extent to which model misspecification results in systematic biases and statistical inconsistency are far from fully understood. However, we know that when evolutionary signal is eroded to the extent that is not, or is barely, distinguishable from confounding noise in the data, then phylogenetic methods are more susceptible to yielding biased estimates [79]. Therefore, we should be alert to potential errors when internal branches are short (and thus may have limited signal) and deep (and thus may have much signal erosion). Phylogenomic scale studies often address such cases, and through the application of large amounts of sequence data also run a greater risk of being substantially mislead by any systematic bias in the inadequately modelled data. Therefore, a major problem of phylogenomics is to determine if seemingly well-supported relationships are the result of systematic bias [16]. *PhyQuart* is motivated by this problem. Our results demonstrate that conventional ML inference can fail when there is strong branch length heterogeneity even when there is only seemingly very minor ML model misspecification. They also provide proof of concept for the idea that (at least for our simulated data and for long alignments) it is possible to design methods that can outperform conventional ML inference in those cases where ML does not perform well. These are the cases where accurate phylogenetic inference is most difficult and additional tools are most needed. *PhyQuart* is based on consideration of the evidential significance of observed site-patterns and combines ML estimation (to help correct for convergence) with *Hennigian* logics which are disregarded in conventional ML analyses, together with a simple approach to reducing apparent support in proportion to branch length asymmetries.

Our quartet simulations, show that *PhyQuart* and ML are either very successful or, if branch length heterogeneity is very high, are moderately successful (i.e., in 50% of simulations) in identifying correct topologies if either one or three terminal branches are long. In the classic LBA problem, with two long and two short terminal branches in a quartet, *PhyQuart* is quite successful in inferring correct topologies from very heterogenous sequence data if the alignment is large (more than 50 kbp) and can outperform ML, overcoming both long branch attraction and repulsion, independent of the chosen simulation assumptions. In the simulations, rate heterogeneity is rather less important for reconstruction success using *PhyQuart* than using ML. Except with very heterogenous sequence data (*α* = 0.1), *PhyQuart* was successful in the majority of simulated cases even when internal branches were kept very short. The simulations show that the reconstruction success of ML decreases with increasing branch length differences even when there is only very minor model misspecification, whereas the performance of *PhyQuart* is only slightly affected by more extreme branch length conditions. It might be expected then that estimated ML models will often be much more inadequate with real, strongly heterogeneous data whereas the *PhyQuart* site-pattern analysis would be less affected by strongly heterogeneous rates of substitution and branch length inequalities. Certainly that possibility is worth investigating. The overall reconstruction success of *PhyQuart* is worst when if the substitution rate heterogeneity of underlying data is extremely high (*α* = 0.1) and two adjacent-group sequences have very long branches compared to the internal branch. However—as shown by our simulation studies—the observed phylogenetic reconstruction success of ML is even worse for such data. Of course, despite conducting almost 173,000 simulations we have only considered a limited range of possible simulations on just four taxa and we have not taken into account the possibility of other sources of error that may result in or exacerbate model misspecification in real data, such as substantial alignment errors (e.g. [10, 89–94]), non-randomly distributed missing data (e.g. [95–98]), and compositional heterogeneity (e.g. [48, 84, 99–104]).

It must be stressed that the restriction of our comparison of *PhyQuart* with ML to quartet analysis is a substantial one given that quartet analysis is considered to exacerbate LBA. Thus we cannot generalise from our results to say that *PhyQuart* will ever outperform conventional ML applied, as it usually is, to larger phylogenetic trees, but this merits investigation if *PhyQuart* is to be of any practical use and further simulation studies investigating this are under way. Despite its potential drawbacks, the benefit of the the computational simplicity of quartet analyses is two-fold, allowing consideration of the evidential significance and calculation of expected frequencies of a small number of site patterns in the development of the *PhyQuart* score, and the ability to obtain and compare these scores for all three quartet tees and thereby get an indication of the strength of the signal detected by *PhyQuart*. Thus, *PhyQuart* support for possible quartet trees can be used directly as a quality measure for how good a data set fits to alternative quartet relationships before ML tree inference or for existing/published tree topologies. Based on our simulations, we suggest that in cases where, for any quartet of taxa, there are two long and two short terminal branches (and thus the potential for classical LBA) and ML and *PhyQuart* both provide good support the same relationship we can be more confident that the ML inference is not the result of LBA. Conversely, where *PhyQuart* and ML provide good support for conflicting relationships or in cases in which *PhyQuart* shows strong contradictory split support for at least one of the other two alternative quartet trees, then we should be more concerned that ML might be being misled by LBA. This does not directly imply that *PhyQuart* supports the correct quartet topology, but it should be seen as an indication that the initially ML-reconstructed topology should be handled with caution. Furthermore, it can be stated that the higher the conflict of *PhyQuart* support for a given quartet tree, the more suspicious is the phylogenetic value of the data.

However, *PhyQuart* is likely to be useful only with large alignments such as in phylogenomic supermatrices and some next generation data types such as RADseq, and is not recommended for shorter sequences such as single gene analyses where stochastic errors in the split split-supporting site-pattern estimation are expected to dominate when trying to infer short internal branches. However, there is probably substantial room for improving the *PhyQuart* approach. For example, to estimate the amount of potentially convergent split signal for a given quartet tree *PhyQuart* uses a simple mean score derived from the two alternative quartet trees. Given that at most only one of these alternative quartet trees could be correct, this scoring function can be expected to differ from the actual number of convergences. The correction factor *ω*, which is used to reduce the counted observed and ML estimated number of positions with relevant site-patterns to approach a more balanced branch-length ratio, depends on the smallest number of observed singleton site-patterns and the total number of these. In our simulation, this reduces the impact of systematic bias in *PhyQuart*, especially in reconstructing quartet topologies with moderate and strong branch length differences (a detailed comparison of reconstruction success with and without correction factor *ω* for simulations with both Felsenstein and Farris topologies using alignments of 250 kbp sequence length are given in the supplementary information S1 Fig). However, due to varying substitution rates along branches and differences in multiple substitutions, the number of observed singleton site-patterns is unlikely to be linearly correlated with the number of split-supporting site-patterns and this might be expected to leads to underestimation of *ω*. Additionally, *PhyQuart* currently ignores potentially useful information in ambiguity states (e.g. [105–107]), or indel events (e.g. [108–110]). Another desiderable extension is for *PhyQuart* to be able to handle data partitions. Clearly, *PhyQuart* is not perfect, but it points the way to new split-supporting site-pattern based methods that allow users to investigate conflicting signals in macromolecular sequence data.

Whereas our simulations have focussed upon proof of concept using only quartets, the PENGUIN software allows the analysis of all quartets of taxa in larger trees, or form predefined quartets of multitaxon clans, and provides a new tool for evaluating contradicting signals that can be used to assess the robustness of relationships within a more complex tree. Generally, it can be stated that the higher the observed contradictory split signal, the more questionable is the reliability of the corresponding branch in a tree and the more suspicious are any high support values for that branch. The PENGUIN software allows users to produce a graphical output summarising signal strengths found for each sequence quartets. This may also be useful for identifying individual rogue taxa that are difficult to place due to ambiguous or weak phylogenetic signal [111]. This characteristic of rogue taxa should become visible when multiple quartets selected from predefined multi-taxon clans are analysed. We also see potential for *PhyQuart* to be used in combination with quartet-based supertree methods (e.g. [112, 113]), of which there are many, and for development of networks summarising conflicting signal. Because, unlike ML, the method makes use of the distinction between plesiomorphy and apomorphy it may provide information on the probable location of the root in trees or networks independent of any consideration of outgroups. The availability of split support information for all three possible quartet relationships and two alternative directions of character evaluation along the innermost branch can be seen as an advantage of the *PhyQuart* approach over conventional ML quartet analyses. The information can be further used in supertree techniques to improve the selection of highly informative and thus appropriate quartets (e.g. quartet topologies without much signal conflict). New ideas on how to use the *PhyQuart* information to build supertrees (e.g. through translation into pairwise support distance matrices based on quartet analyses of multiple-taxon clans) have already been successfully tested in recent test studies and will be published soon.

## Supporting information

S1 Fig Complete results of 4-taxon simulations of 250 kbp long sequences given two elongated branches. Complete results of 4-taxon simulations based on stepwise BL2 elongations of two adjacent or non-adjacent terminal branches given 250 kbp long nucleotide alignment data. The pdf document can be opened with pdf readers like AdobeAcrobatReader, Xpdf, or DocumentViewer.
(PDF)

S2 Fig Complete results of 4-taxon simulations of 250 kbp long sequences given two elongated branches with and without using correction factor. Complete *PhyQuart* results of 4-taxon simulations with and without using correction factor *ω* based on stepwise BL2 elongations of two adjacent or non-adjacent terminal branches given 250 kbp long nucleotide alignment data. The pdf document can be opened with pdf readers like AdobeAcrobatReader, Xpdf, or DocumentViewer.
(PDF)

S3 Fig Complete results of 4-taxon simulations of sequences shorter < 250 kbp given two elongated branches. Complete results of 4-taxon simulations based on stepwise BL2 elongations of two adjacent or non-adjacent terminal branches given nucleotide alignment data < 250 kbp. The pdf document can be opened with pdf readers like AdobeAcrobatReader, Xpdf, or DocumentViewer.
(PDF)

S4 Fig Complete results of 4-taxon simulations of 250 kbp long sequences given a single elongated branch. Complete results of 4-taxon simulations based on stepwise BL2 elongations of one terminal branch given 250 kbp long nucleotide alignment data. The pdf document can be opened with pdf readers like AdobeAcrobatReader, Xpdf, or DocumentViewer.
(PDF)

S5 Fig Complete results of 4-taxon simulations of 250 kbp long sequences given three elongated branches. Complete results of 4-taxon simulations based on stepwise BL2 elongations of three terminal branches given 250 kbp long nucleotide alignment data. The pdf document can be opened with pdf readers like AdobeAcrobatReader, Xpdf, or DocumentViewer.
(PDF)
