# Correction: Can quartet analyses combining maximum likelihood estimation and Hennigian logic overcome long branch attraction in phylogenomic sequence data?

**DOI:** 10.1371/journal.pone.0186617

**Published:** 2017-10-12

**Authors:** The PLOS ONE Staff

There are multiple errors in Fig 1. The authors have provided a corrected version here. The publisher apologizes for the error.

**Fig 1:**
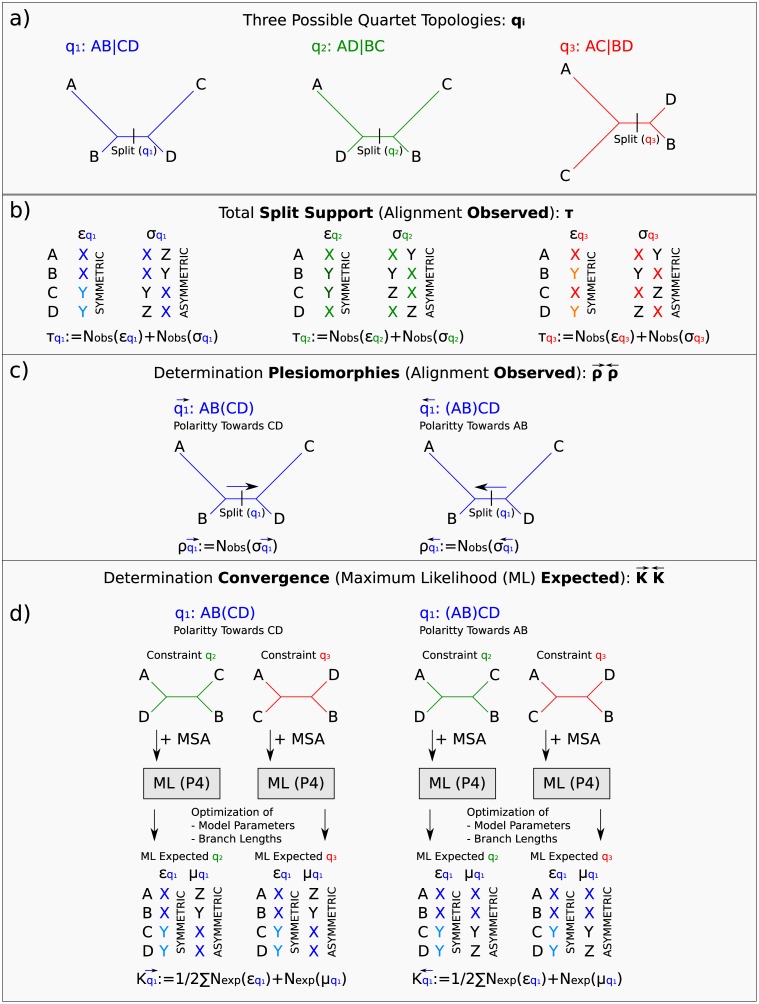
Flowchart of the *PhyQuart* algorithm. Simplified flowchart showing a) each of the three possible quartet relationships for a set of 4 sequences (*q*_1_, *q*_2_, *q*_3_), b) the site-pattern classification of observed (*N*_*obs*_) symmetric ($\xi_{q_{i}}$) and asymmetric ($\sigma_{q_{i}}$) support ($\tau_{q_{i}}$), c) the determination of plesiomorphic (old) split-supporting site-patterns given two different polarities of character transformation along the internal branch of each possible quartet tree, $\rho_{\vec{q_{{}_{1}}}}$ and $\rho_{\overset{\leftarrow }{q_{{}_{1}}}}$, and d) estimation of expected convergent split-supporting site-patterns ($\kappa_{\vec{q_{{}_{1}}}}$,$\kappa_{\overset{\leftarrow }{q_{{}_{1}}}}$) supporting quartet *q*_1_ in ML split pattern estimations using branch length and model optimization on constraint topologies of the other two possible quartet relationships (*q*_2_, *q*_3_).
