# Supplementary figures and images for: Can quartet analyses combining maximum likelihood estimation and Hennigian logic overcome long branch attraction in phylogenomic sequence data?
Source: PLoS One. 2017 Aug 25;12(8):e0183393. doi: 10.1371/journal.pone.0183393 (PMC5571918; doi:10.1371/journal.pone.0183393)

# Simulated: GTR Sequence Length: 250 000 bp

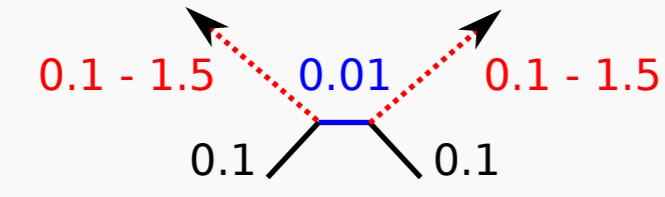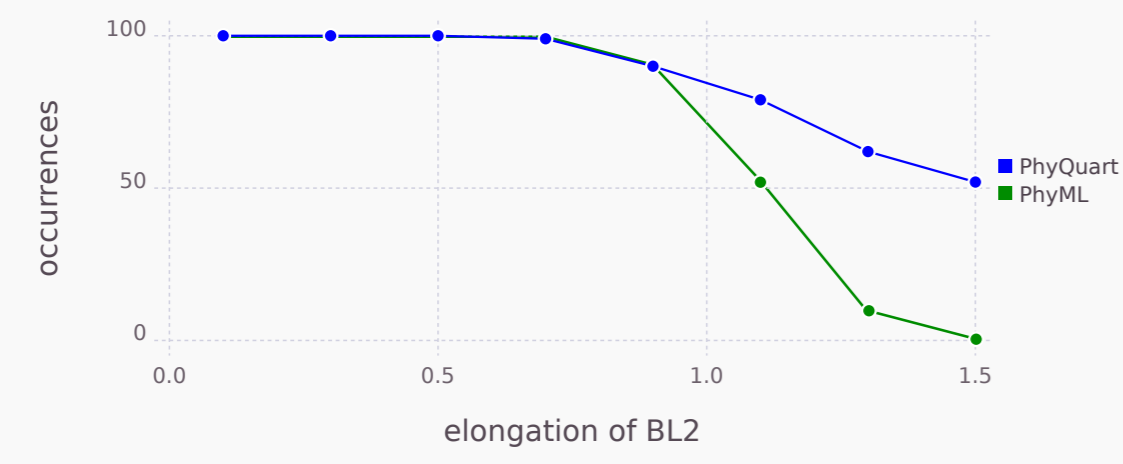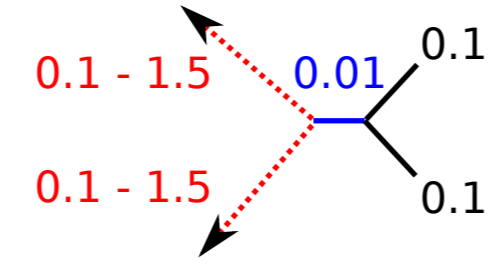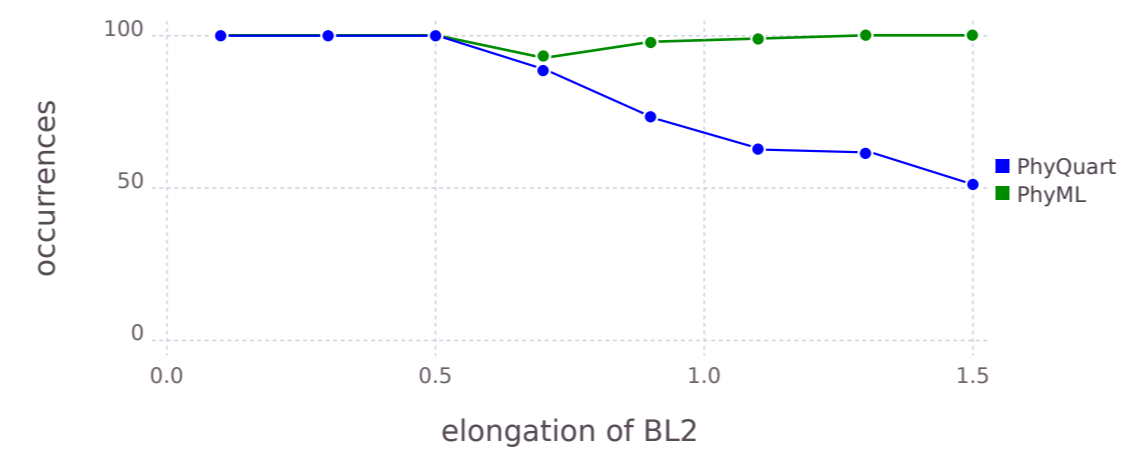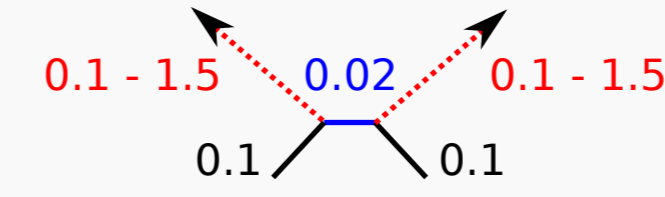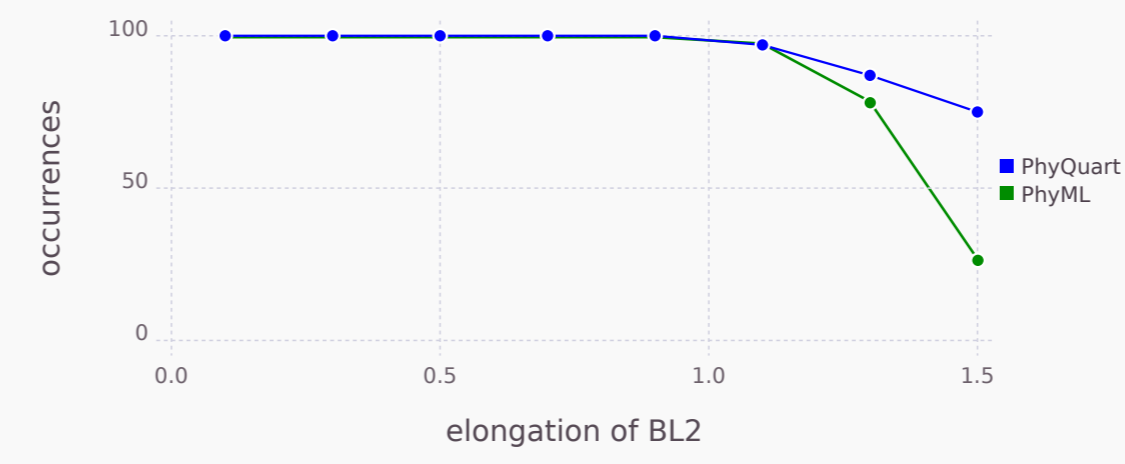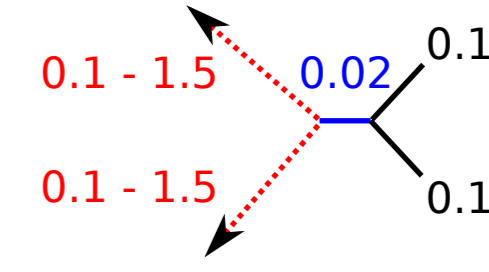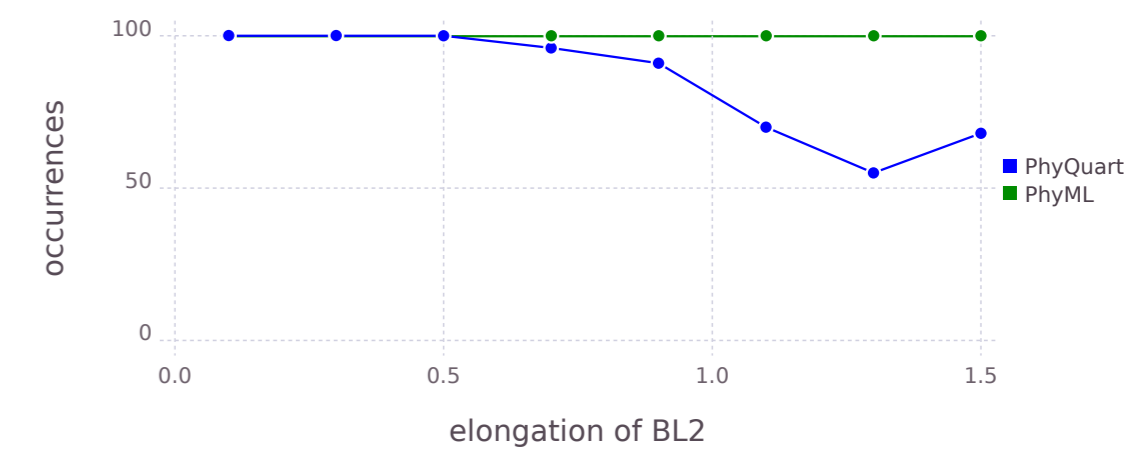

$\alpha=2.0$   
 $p=0.3$

$\alpha=1.0$   
 $p=0.3$

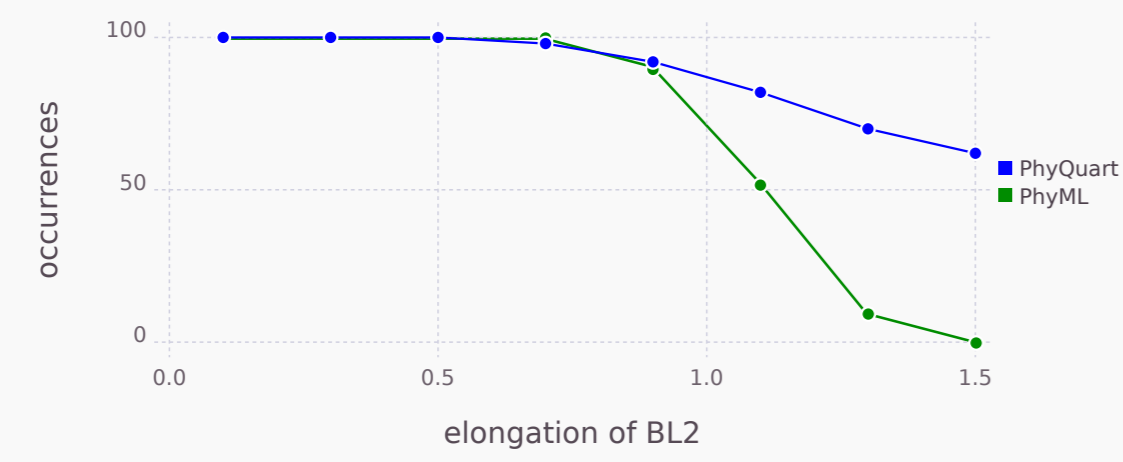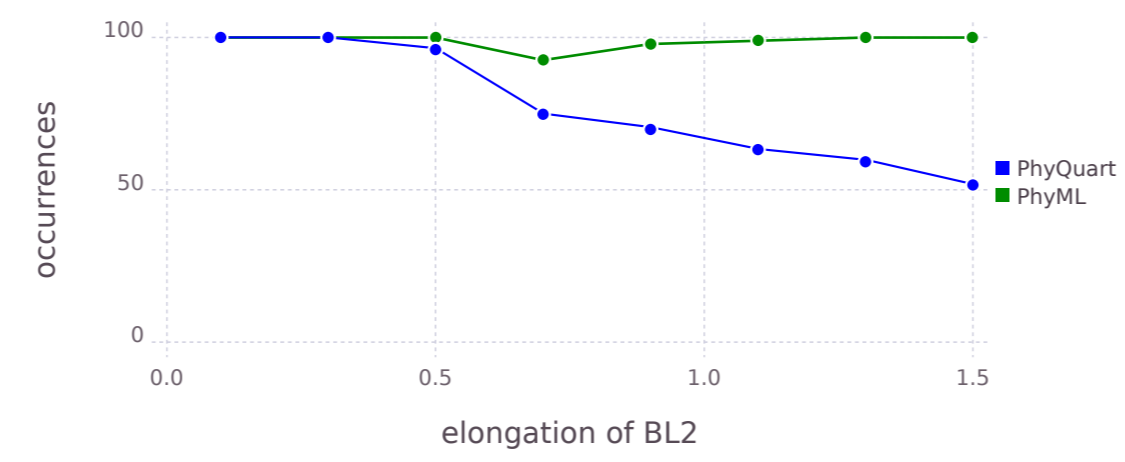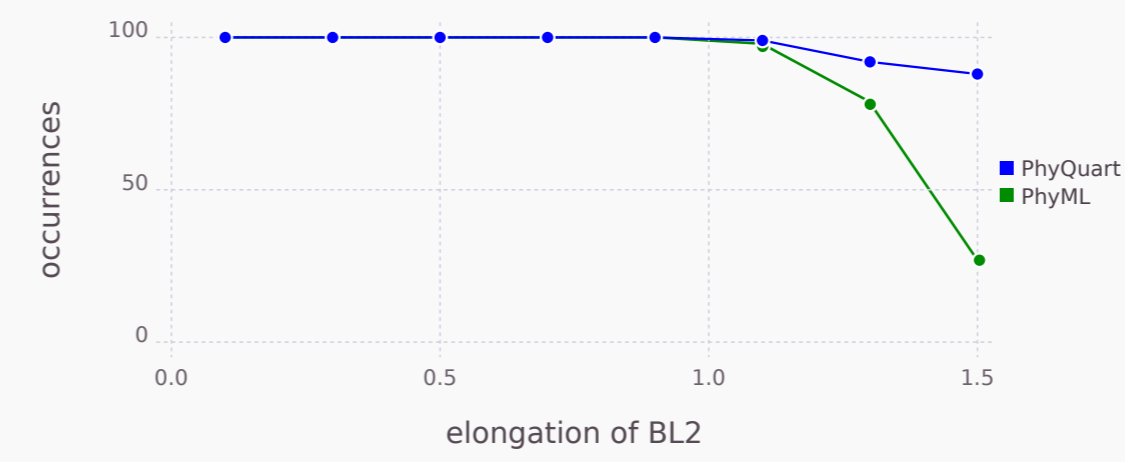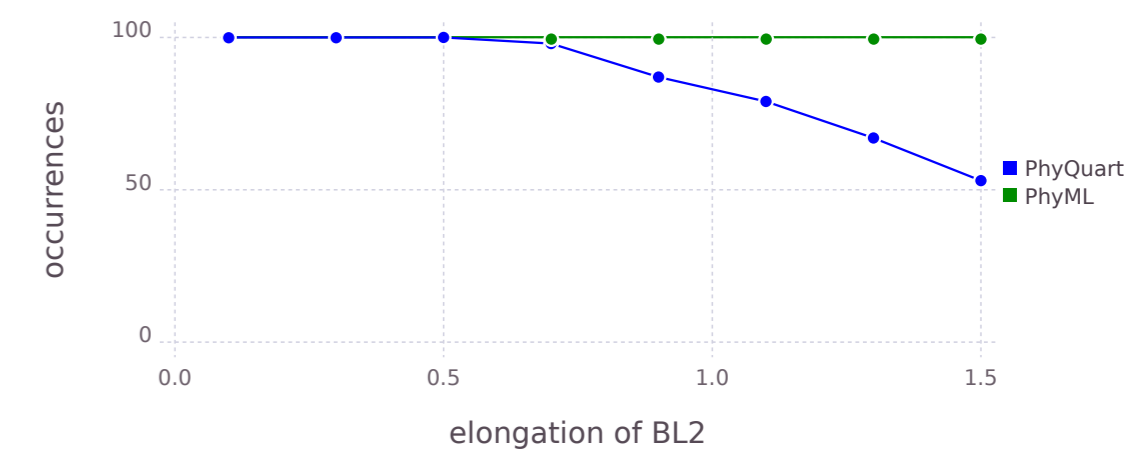

$\alpha=0.7$   
 $p=0.3$

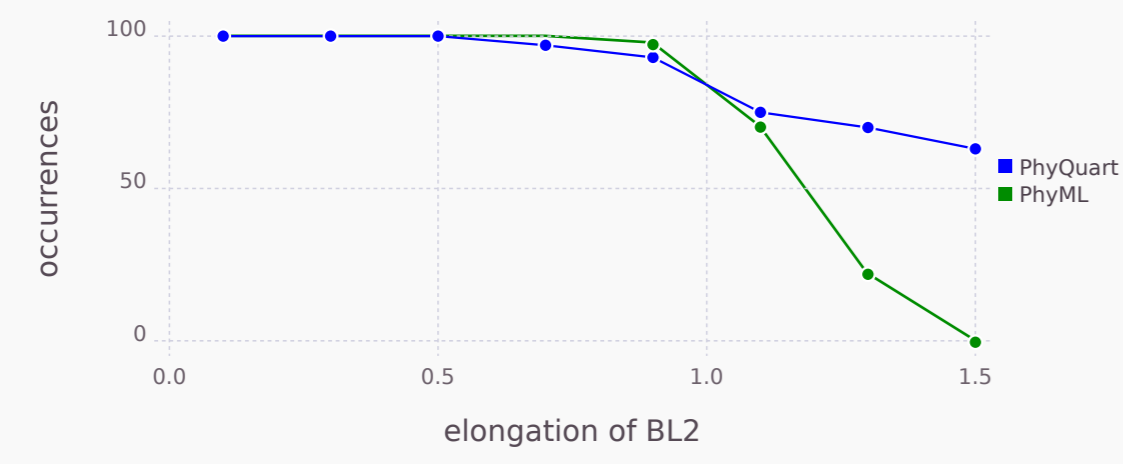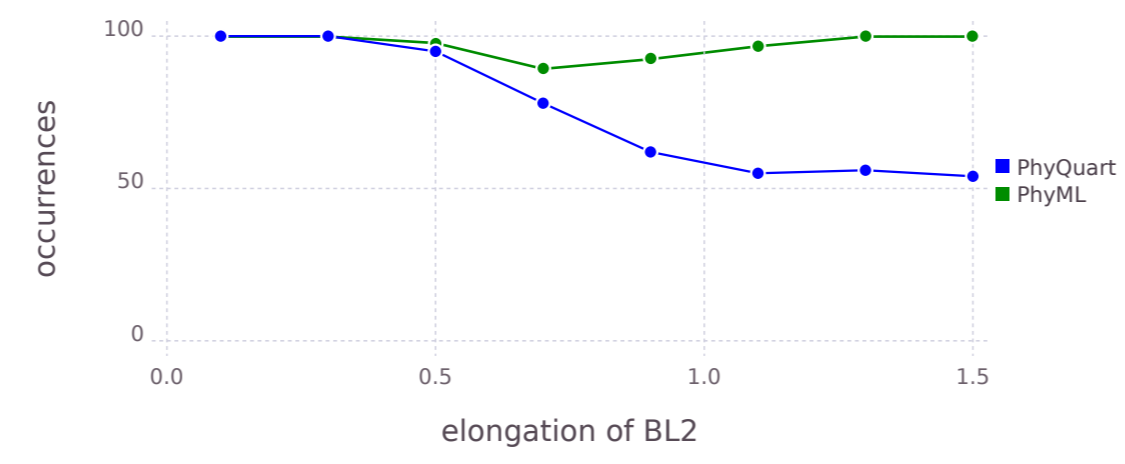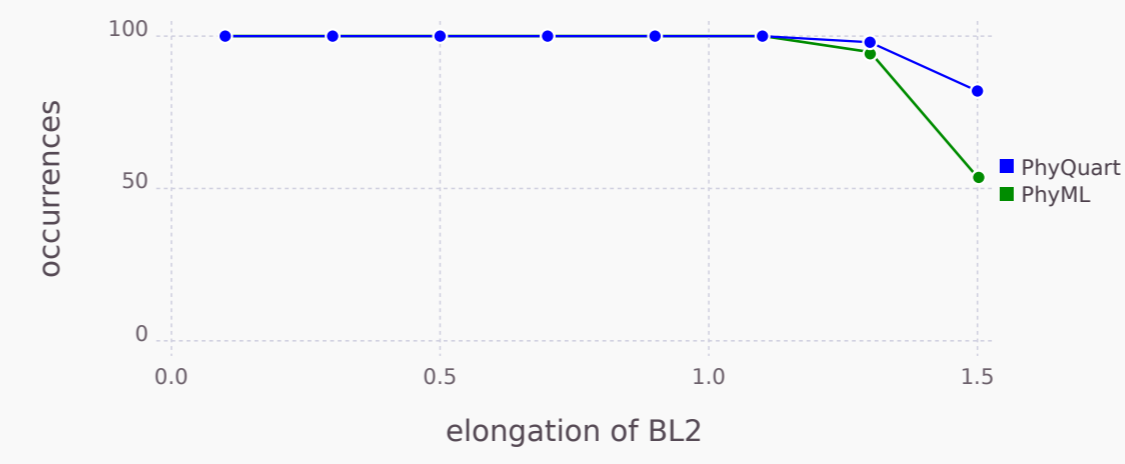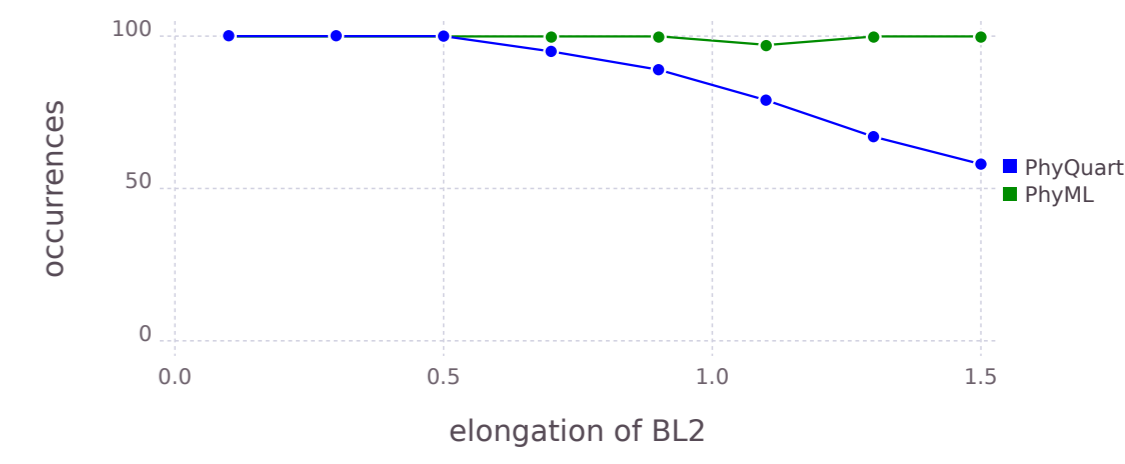

$\alpha=0.5$   
 $p=0.3$

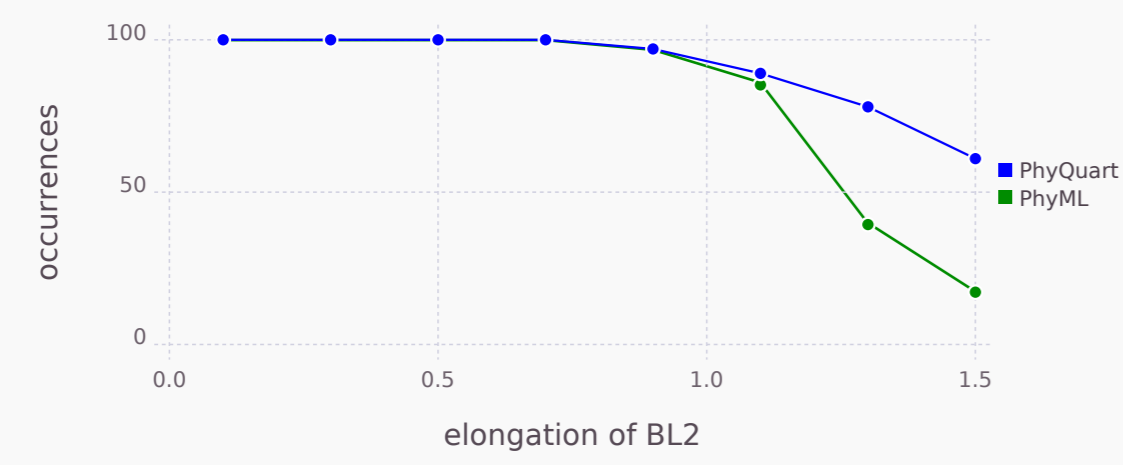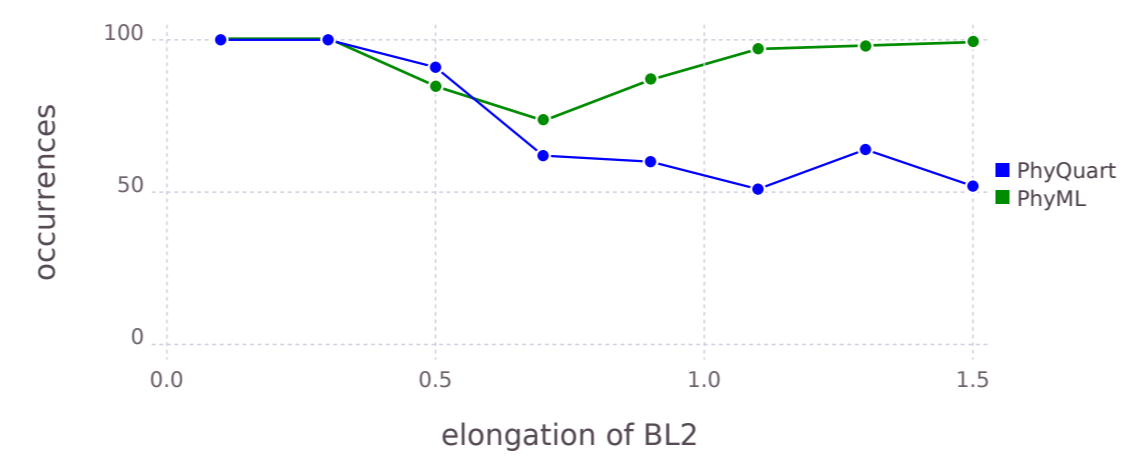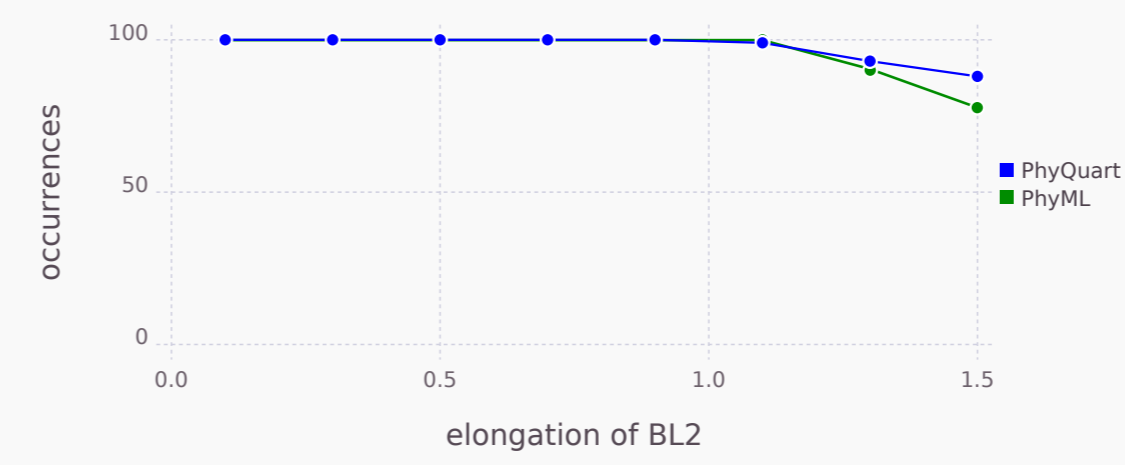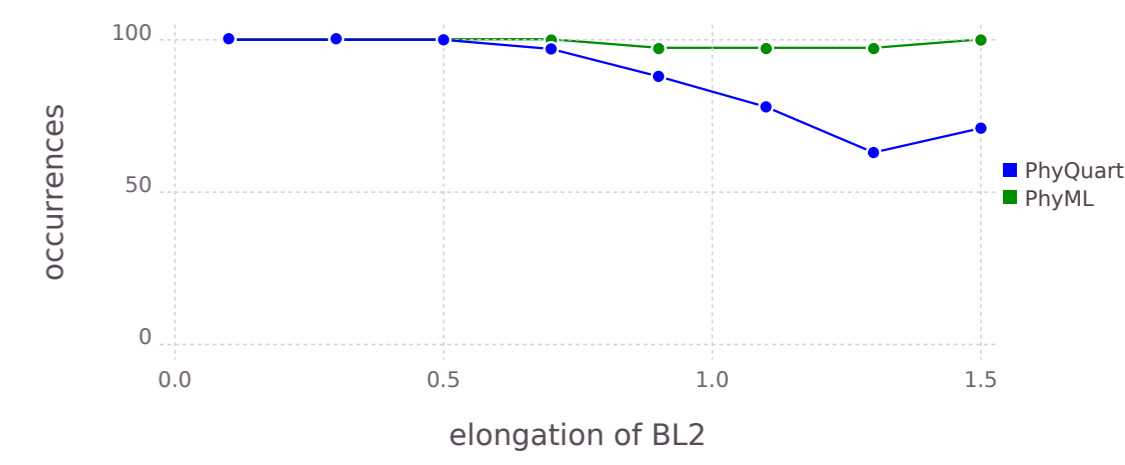

$\alpha=0.3$   
 $p=0.3$

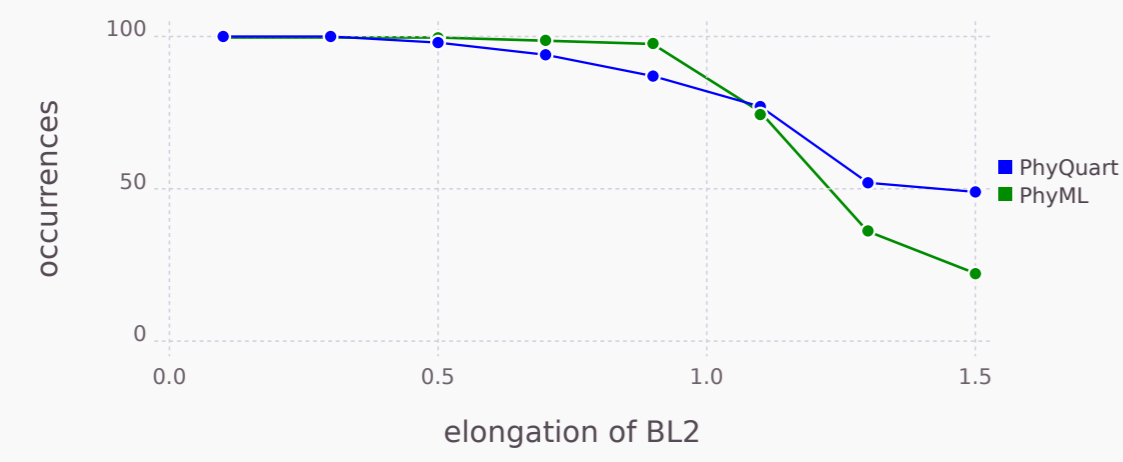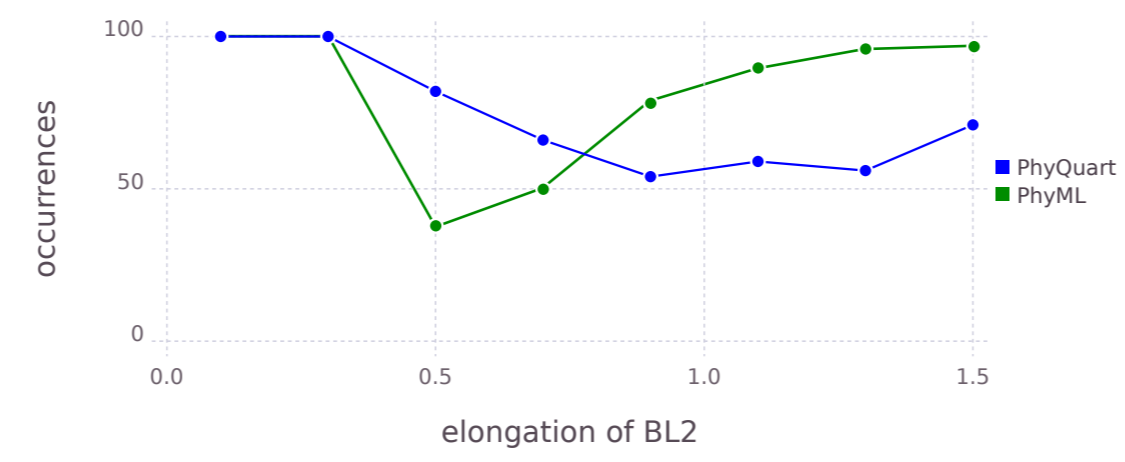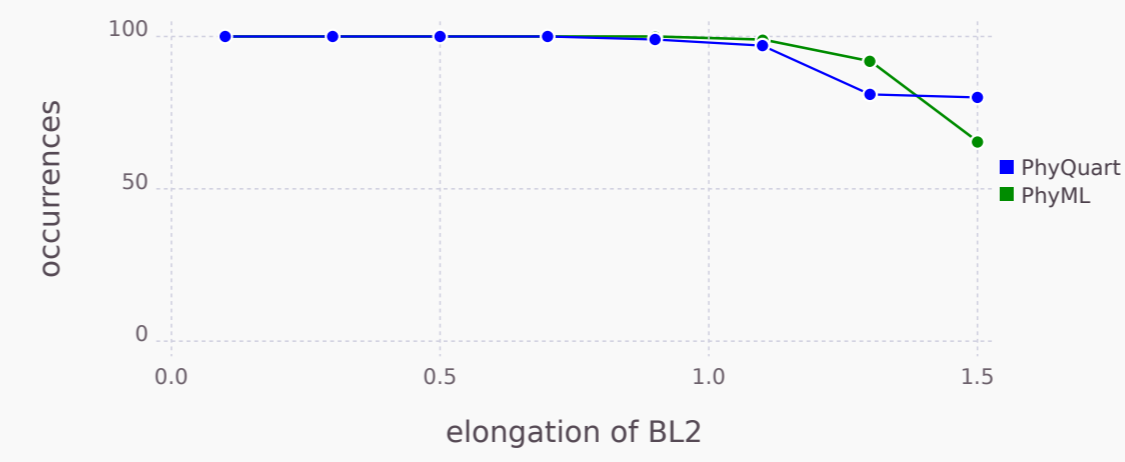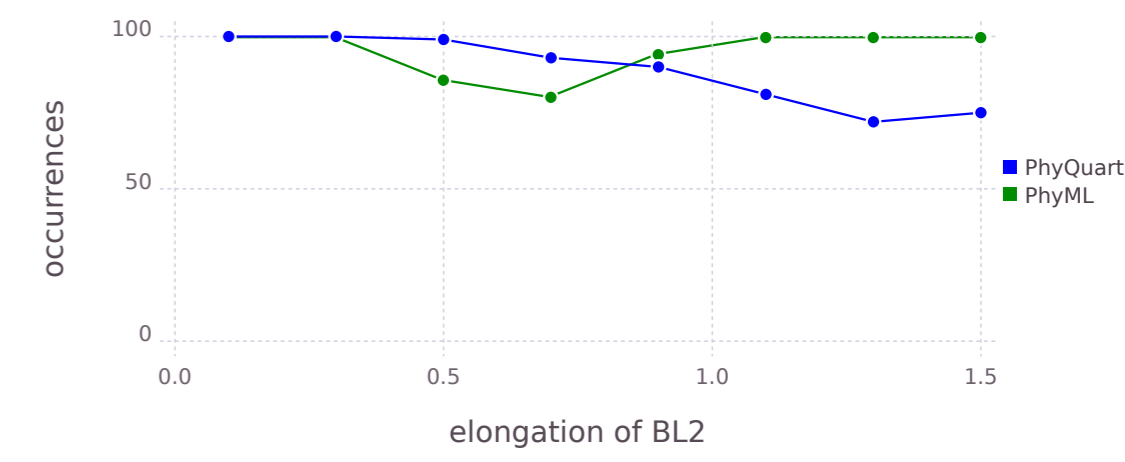

$\alpha=0.1$   
 $p=0.3$

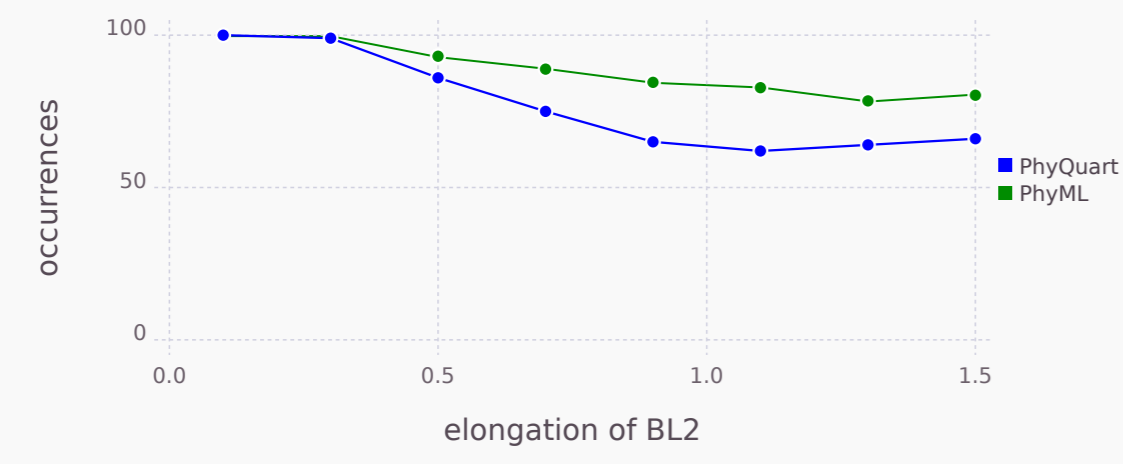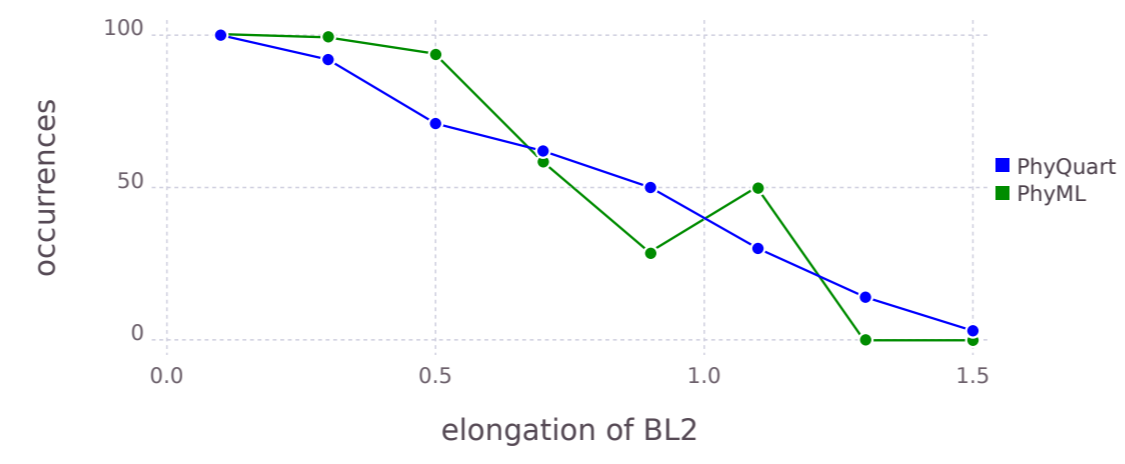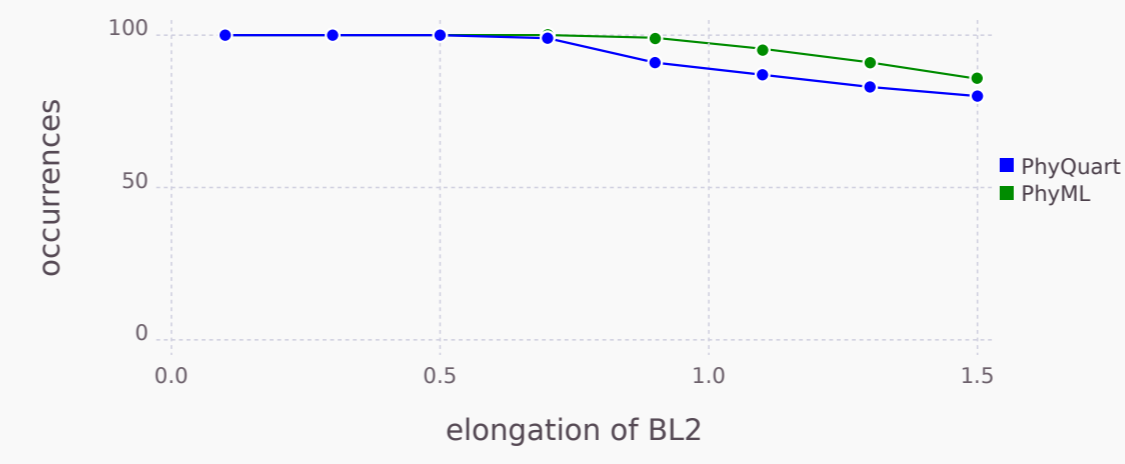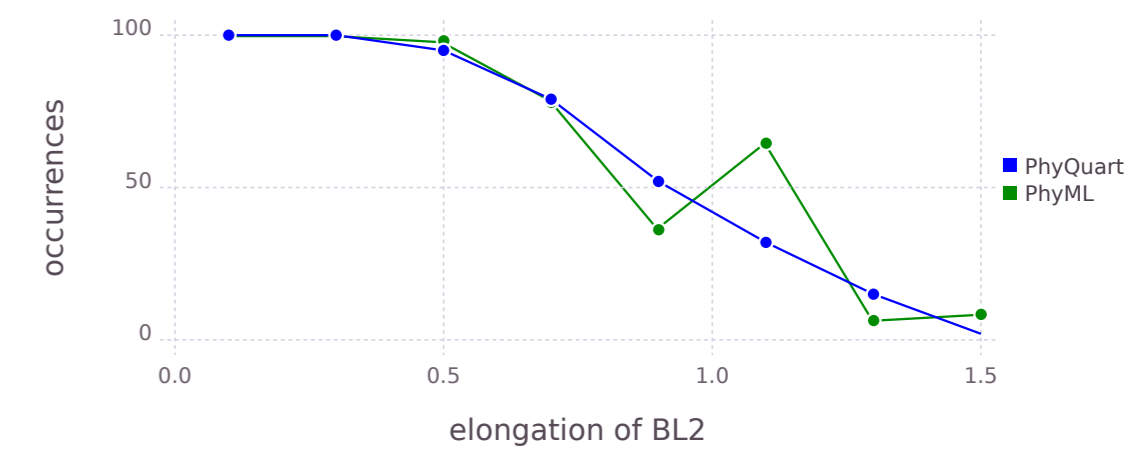

Supplement: S1 Fig — Complete results of 4-taxon simulations based on stepwise BL2 elongations of two adjacent or non-adjacent terminal branches given 250 kbp long nucleotide alignment data. The pdf document can be opened with pdf readers like AdobeAcrobatReader, Xpdf, or DocumentViewer. (PDF) [file pone.0183393.s001.pdf]

# Simulated: GTR Sequence Length: 250 000 bp

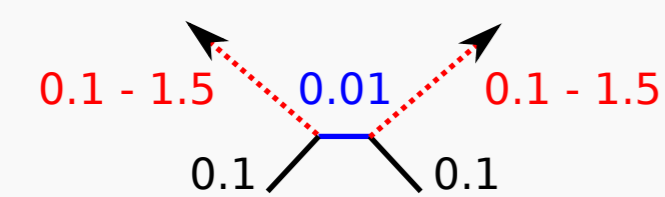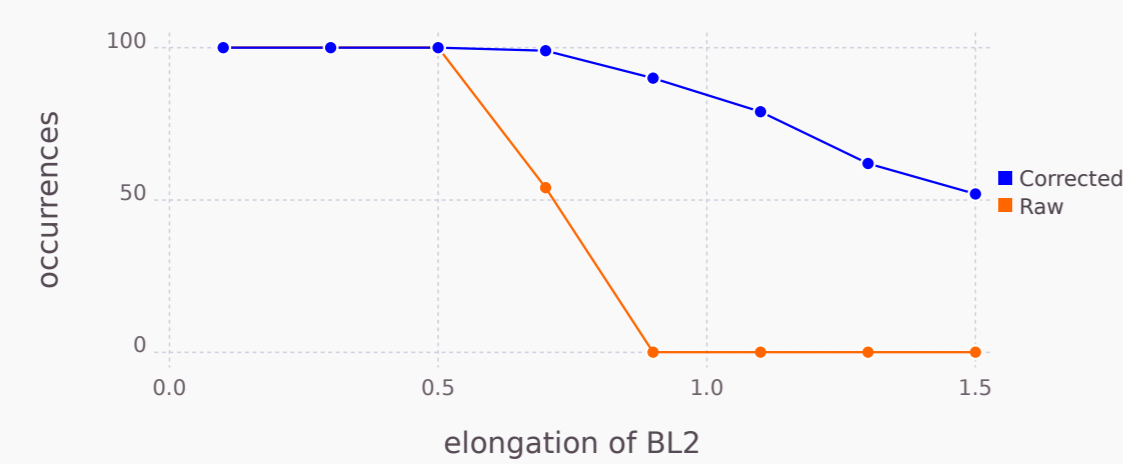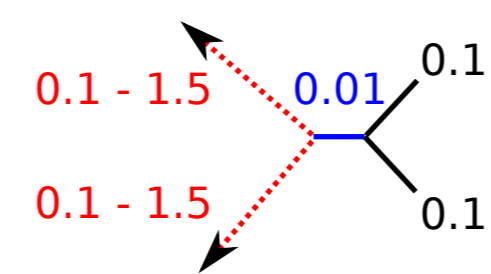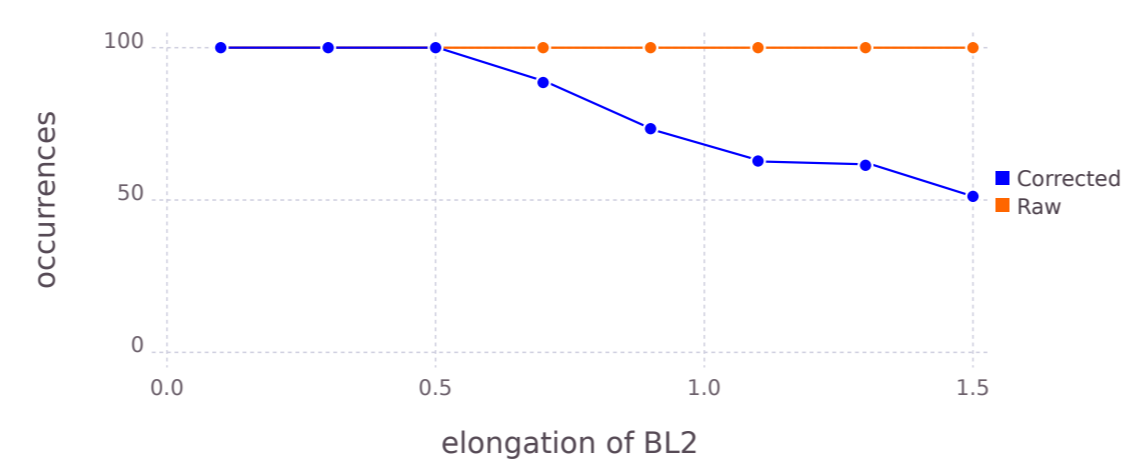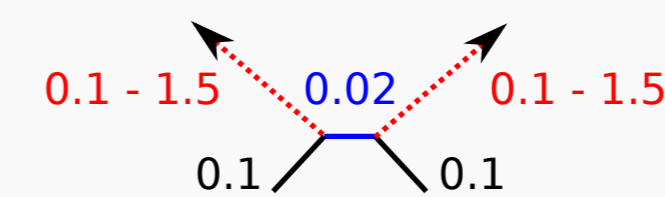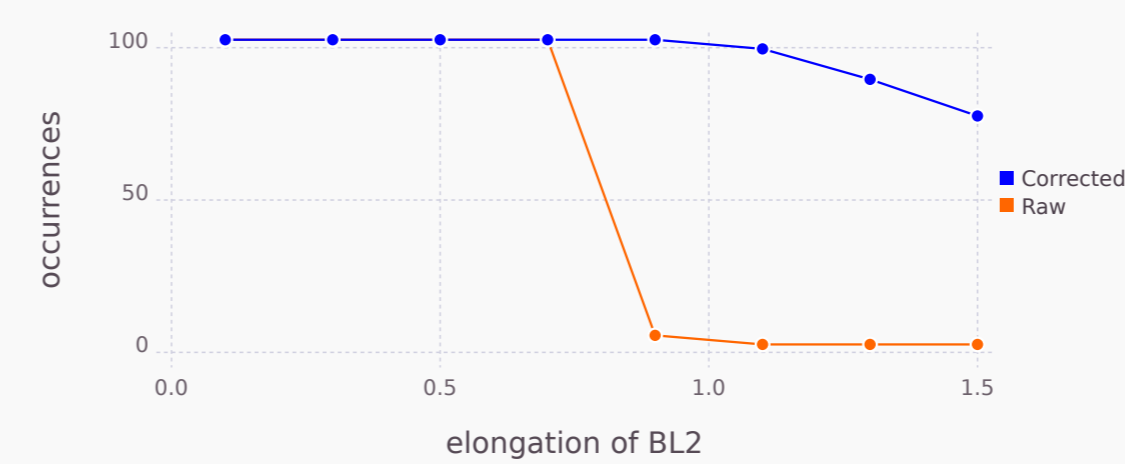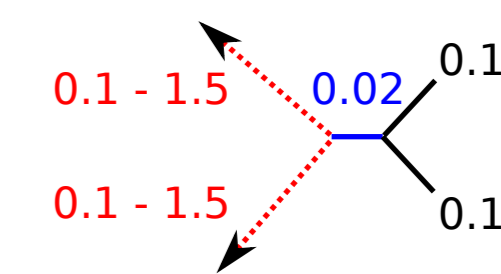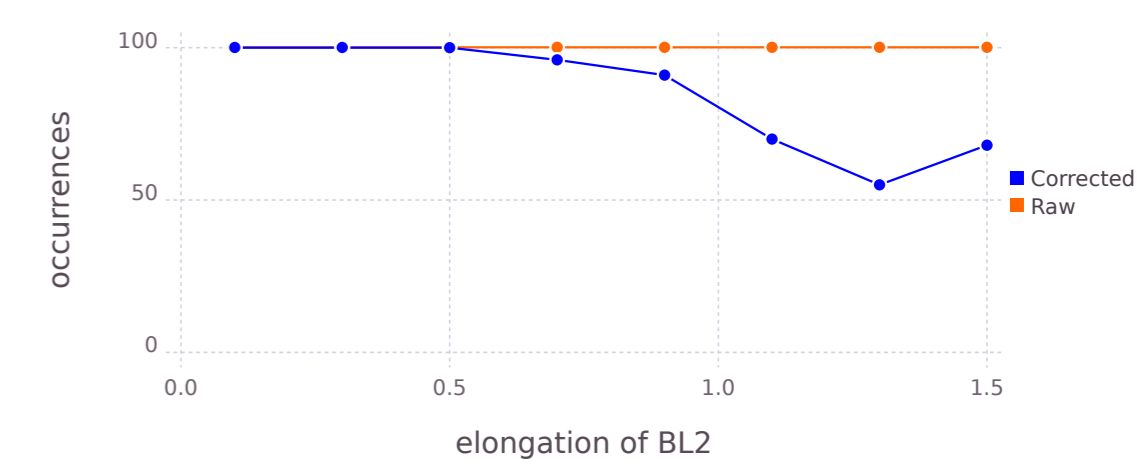

$\alpha=2.0$   
 $p=0.3$

$\alpha=1.0$   
 $p=0.3$

$\alpha=0.7$   
 $p=0.3$

$\alpha=0.5$   
 $p=0.3$

$\alpha=0.3$   
 $p=0.3$

$\alpha=0.1$   
 $p=0.3$

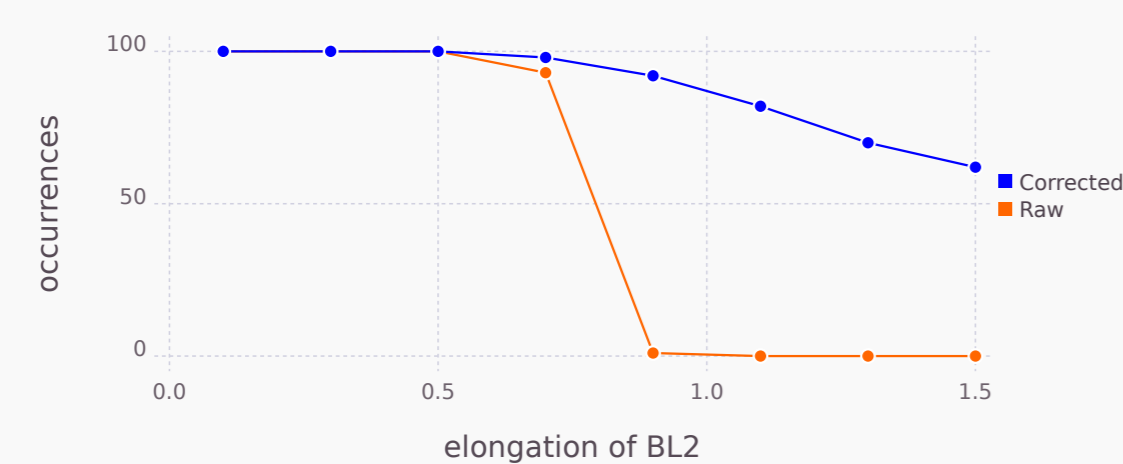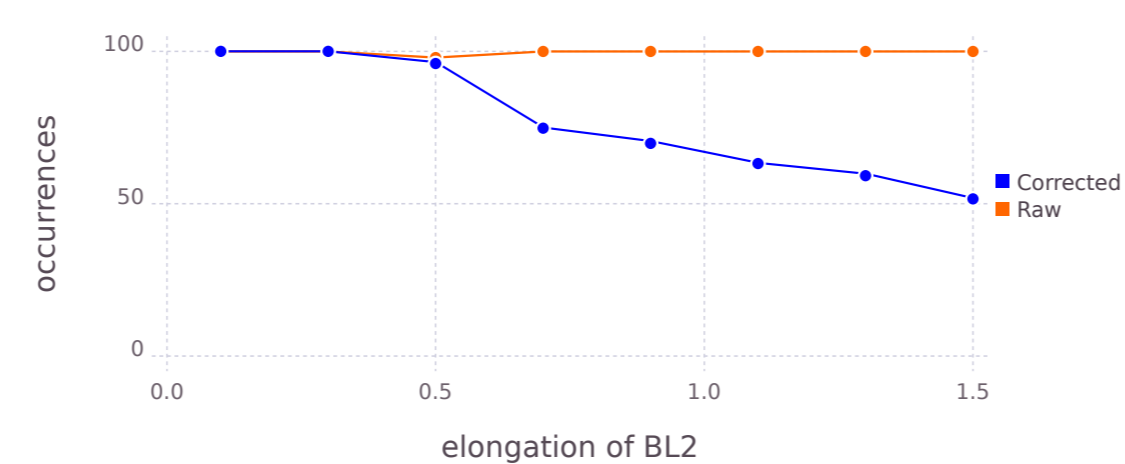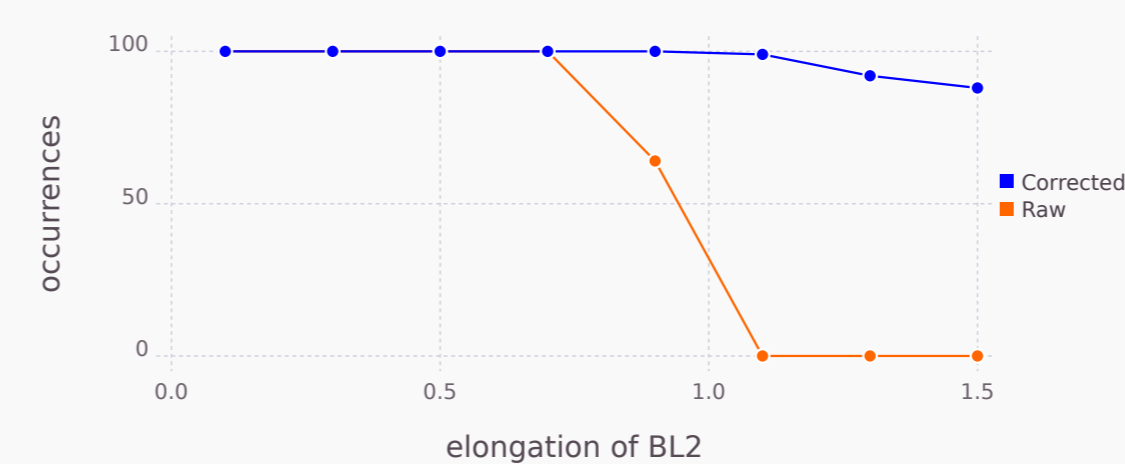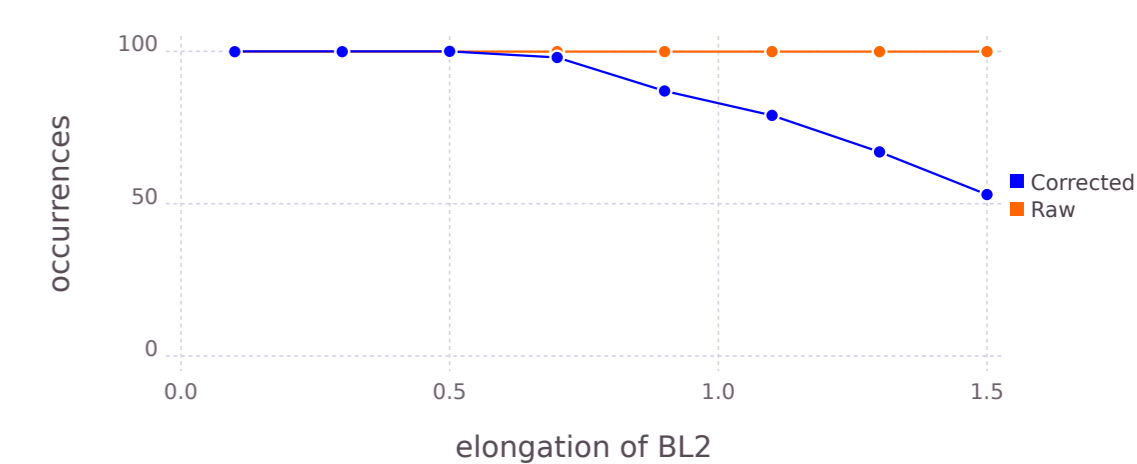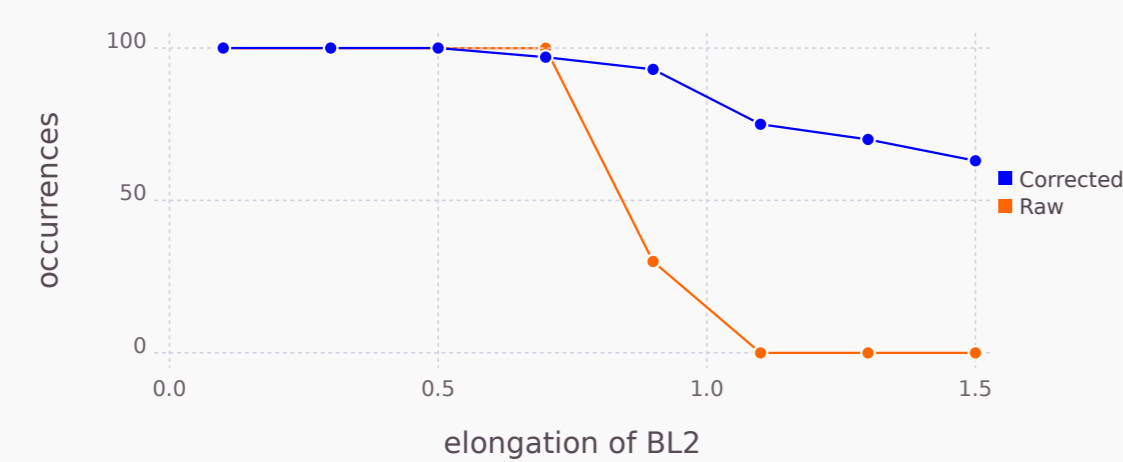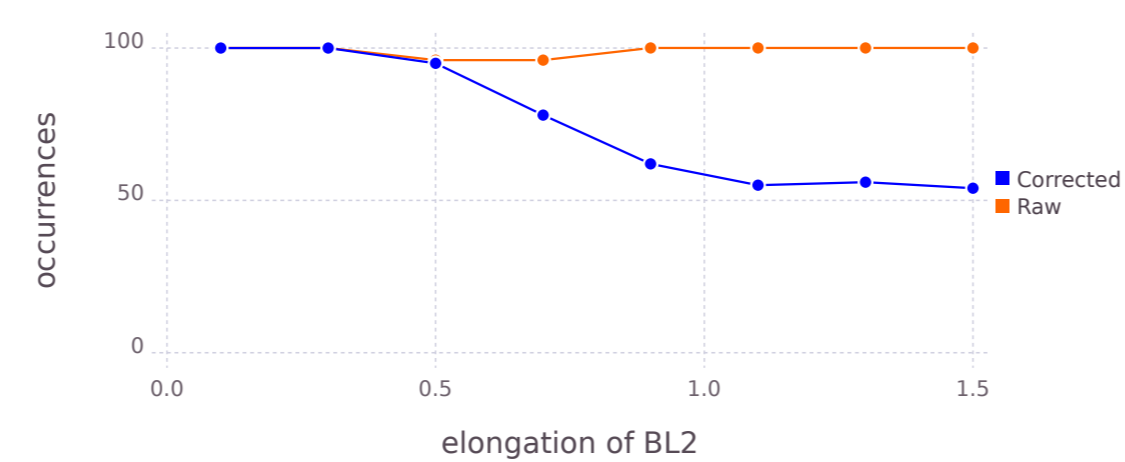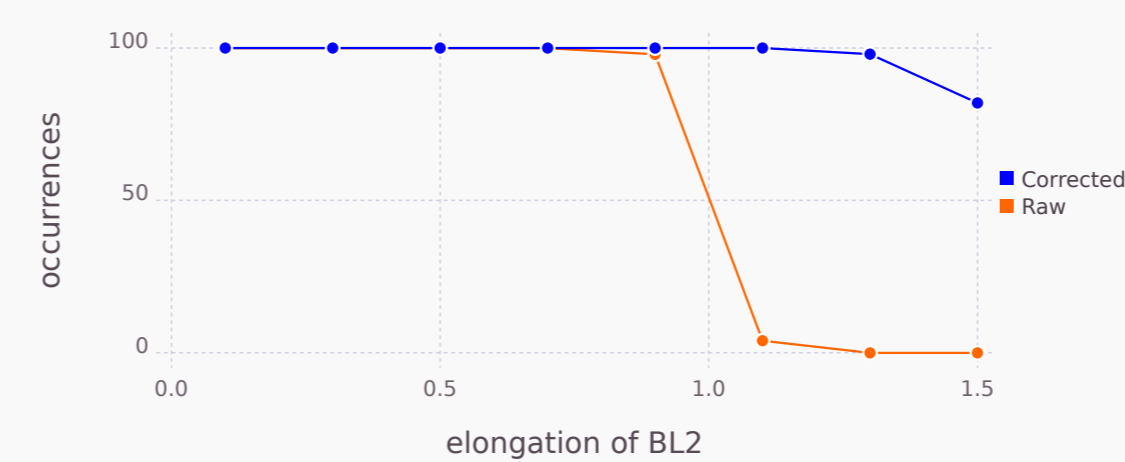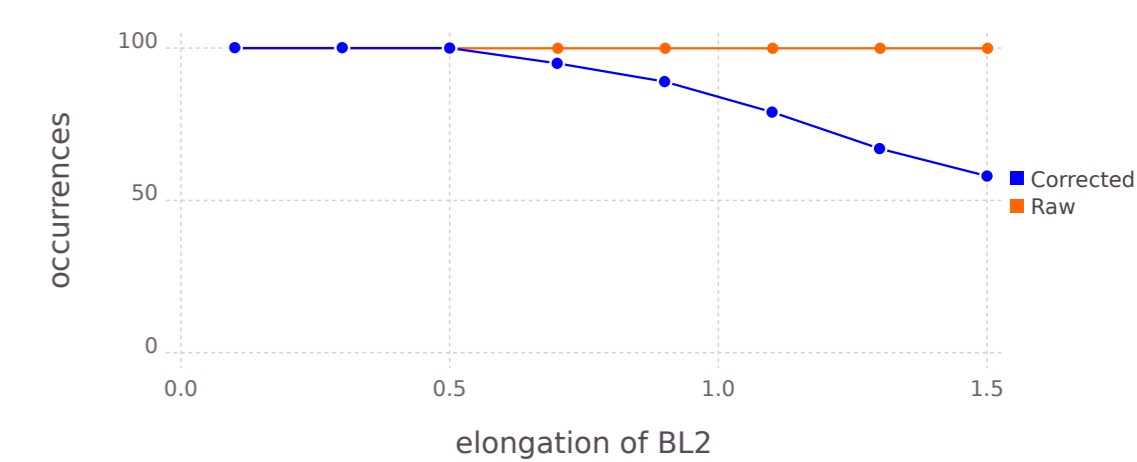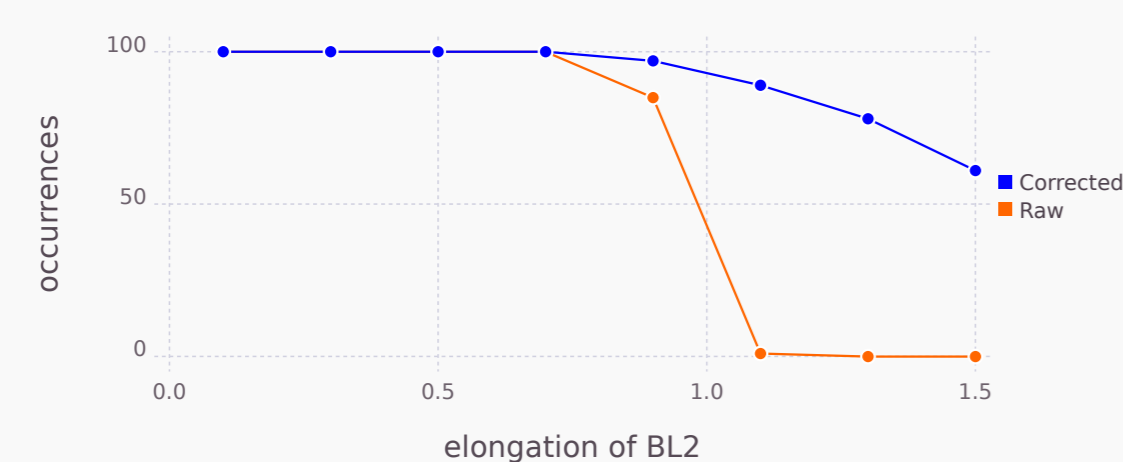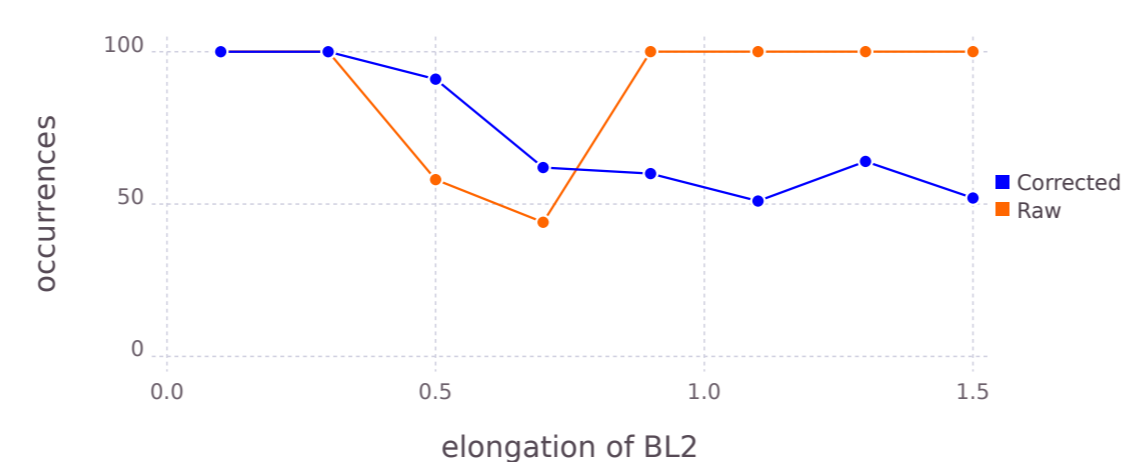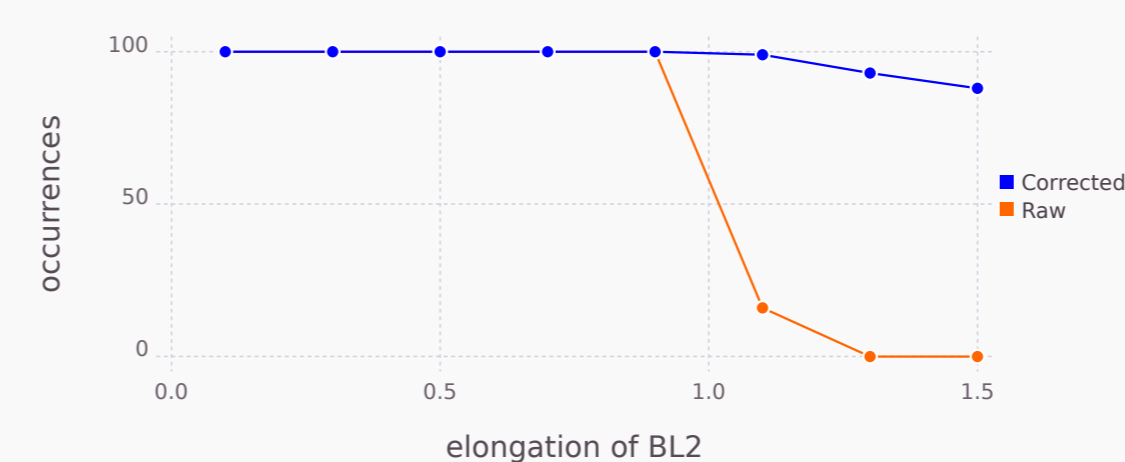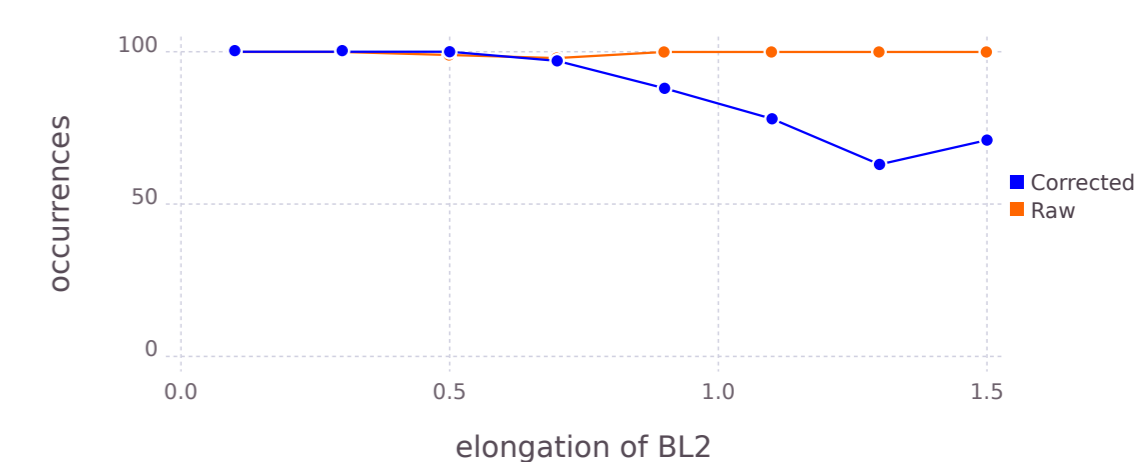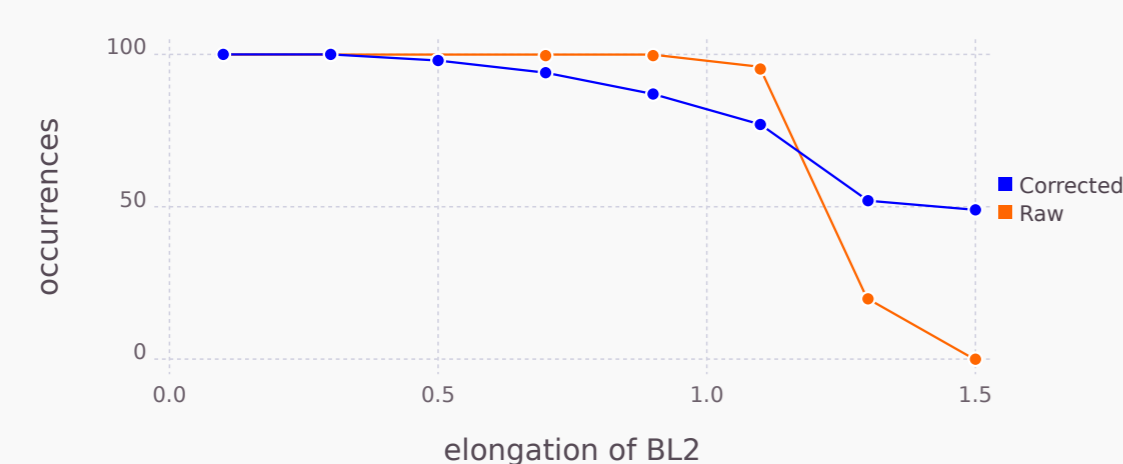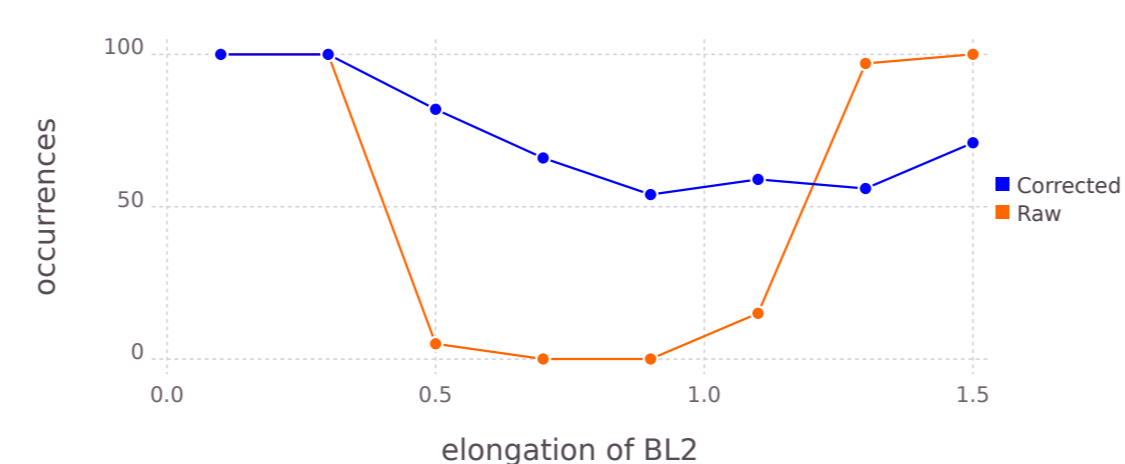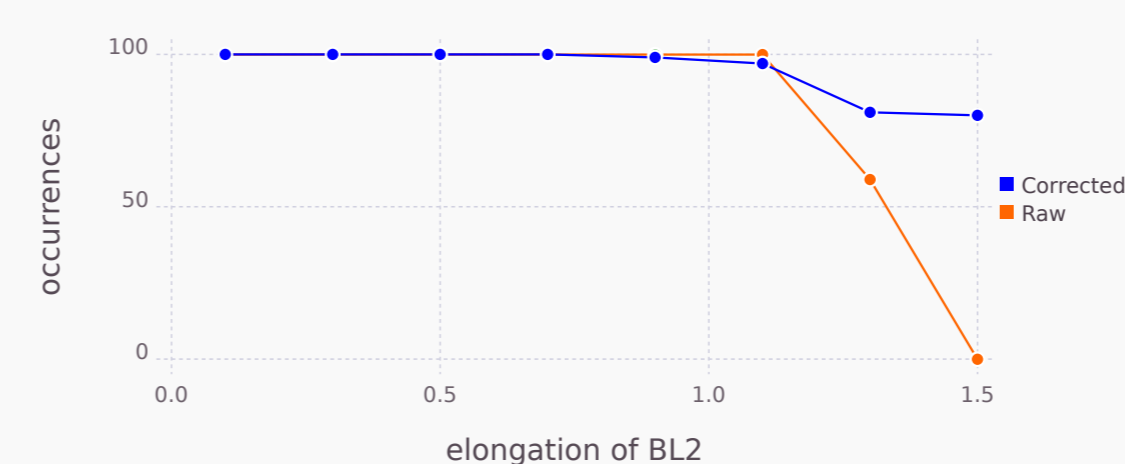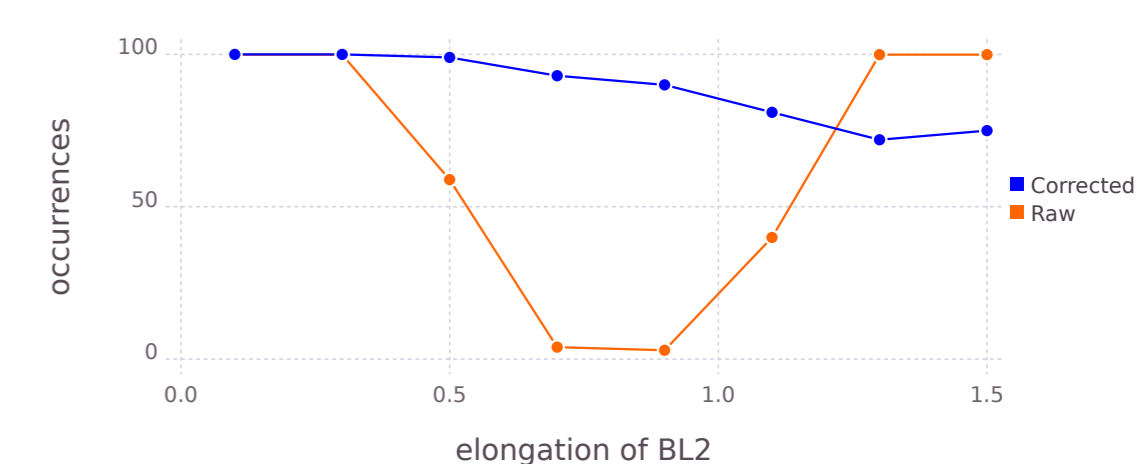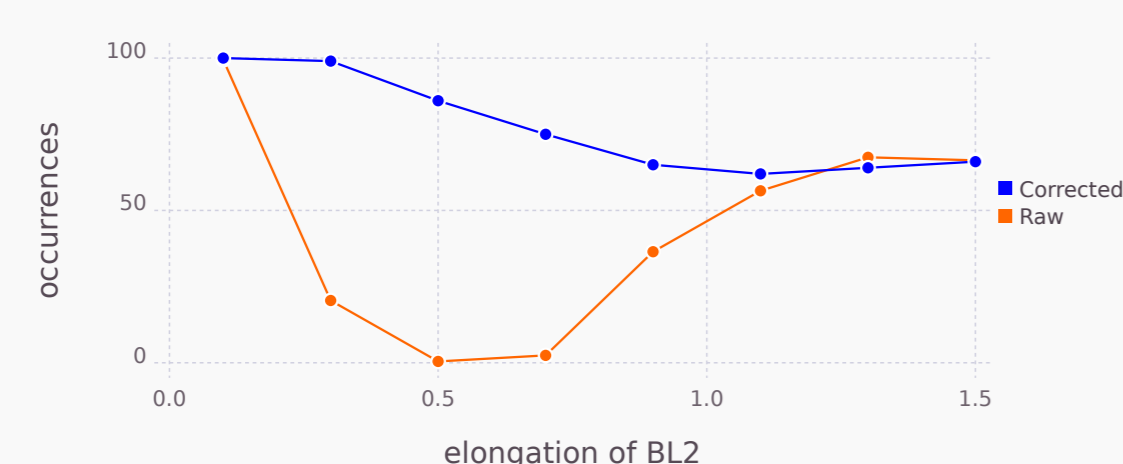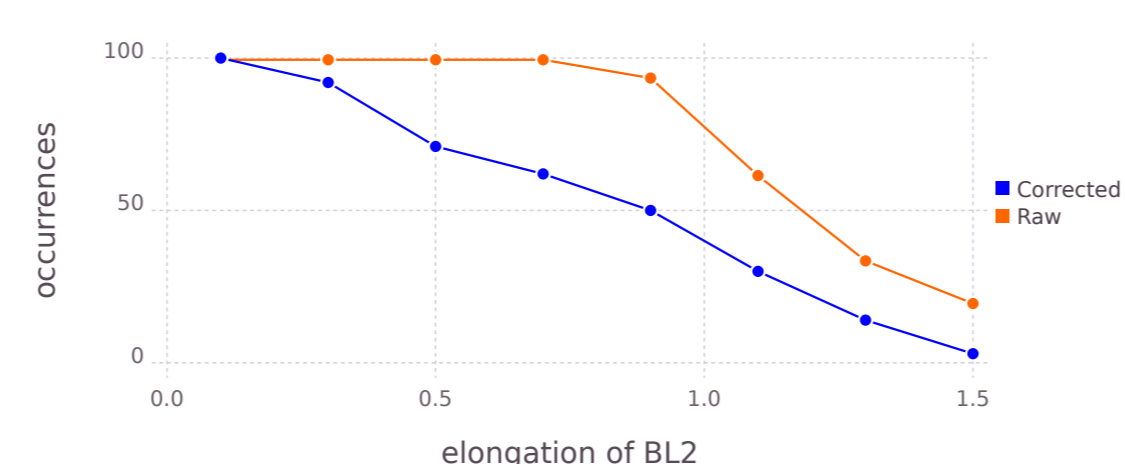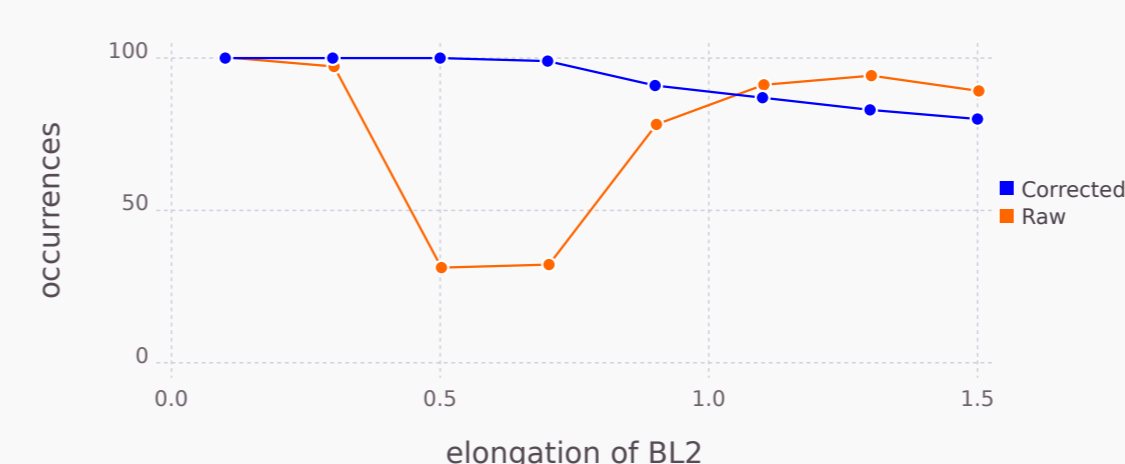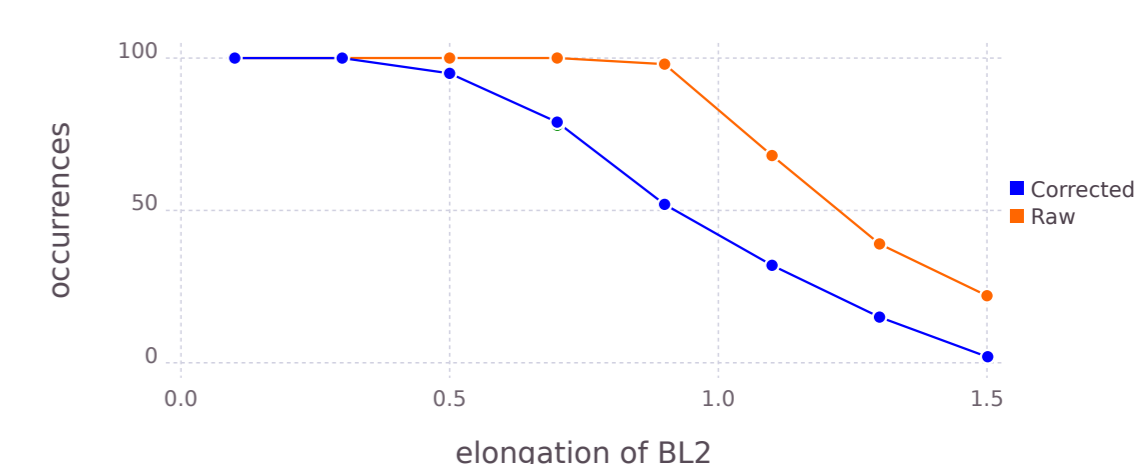

Supplement: S2 Fig — Complete PhyQuart results of 4-taxon simulations with and without using correction factor ω based on stepwise BL2 elongations of two adjacent or non-adjacent terminal branches given 250 kbp long nucleotide alignment data. The pdf document can be opened with pdf readers like AdobeAcrobatReader, Xpdf, or DocumentViewer. (PDF) [file pone.0183393.s002.pdf]

Simulated: GTR Sequence Length: 250 000 bp

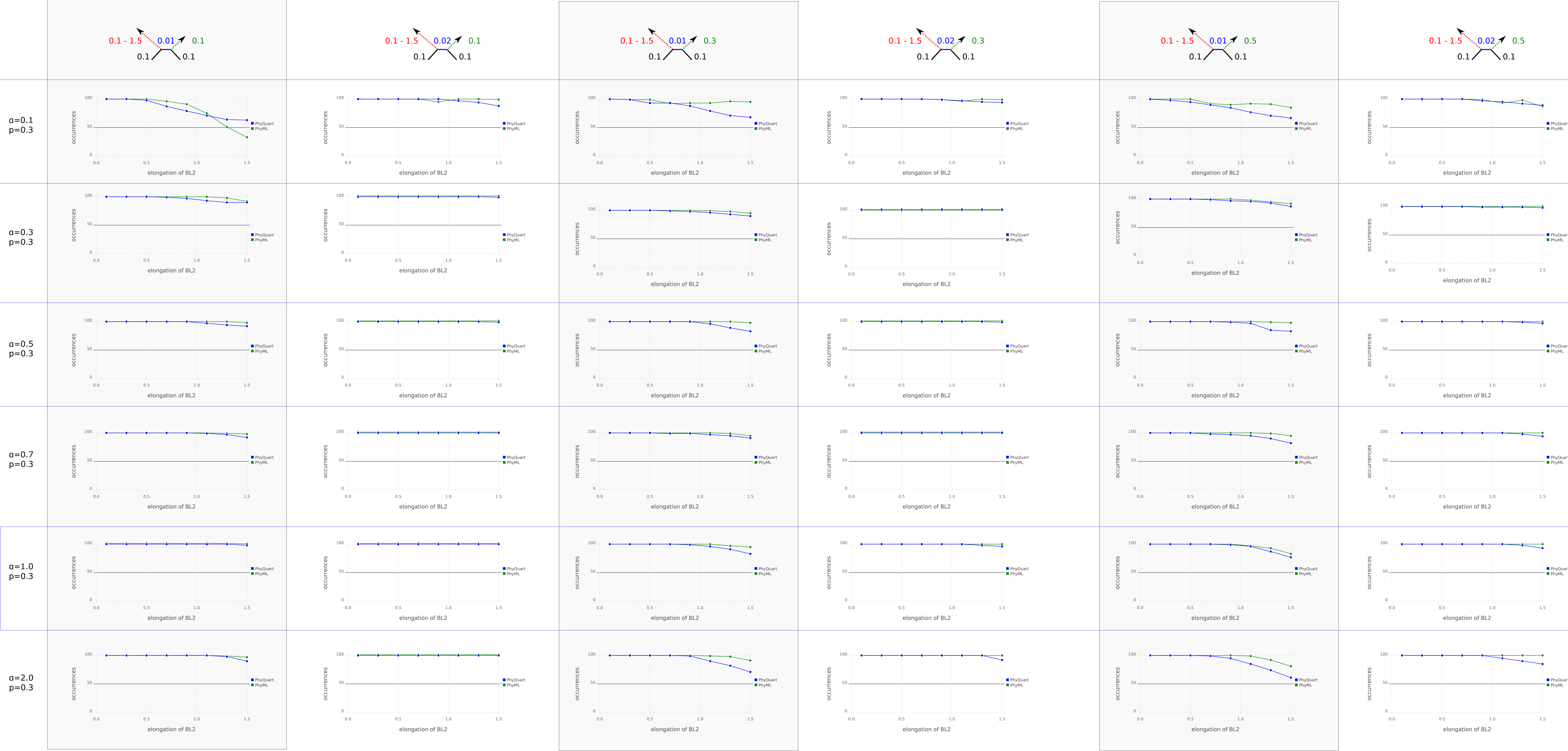

Supplement: S4 Fig — Complete results of 4-taxon simulations based on stepwise BL2 elongations of one terminal branch given 250 kbp long nucleotide alignment data. The pdf document can be opened with pdf readers like AdobeAcrobatReader, Xpdf, or DocumentViewer. (PDF) [file pone.0183393.s004.pdf]

Simulated: GTR Sequence Length: 250 000 bp

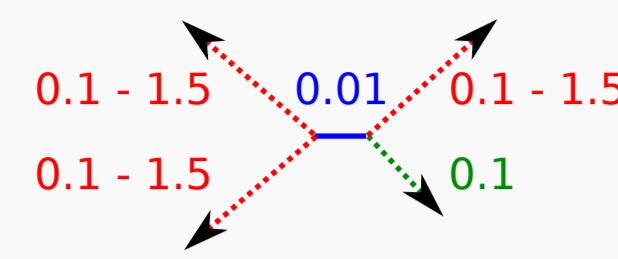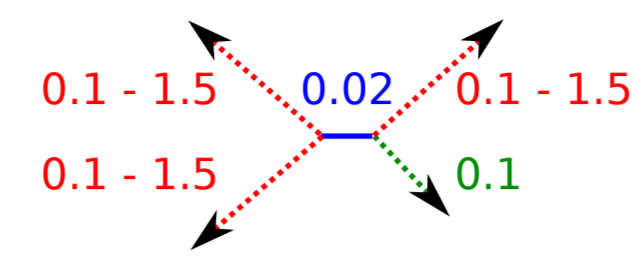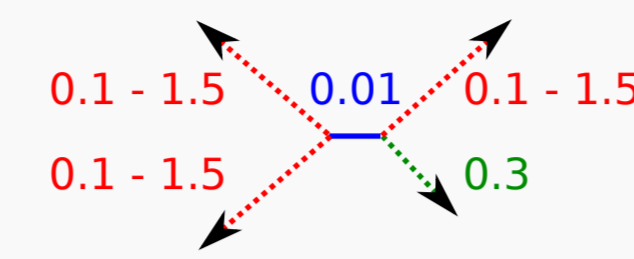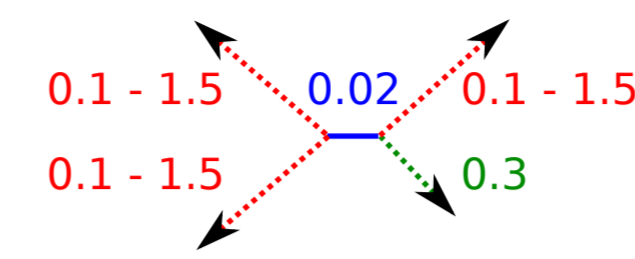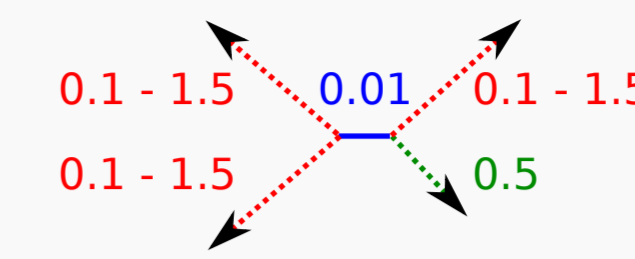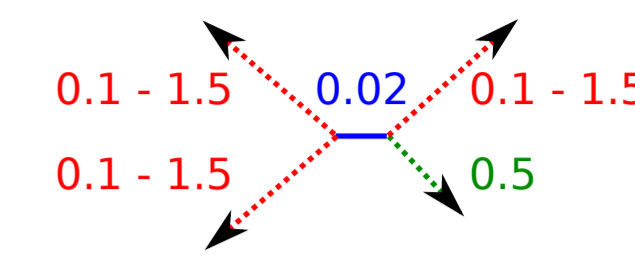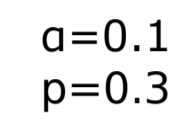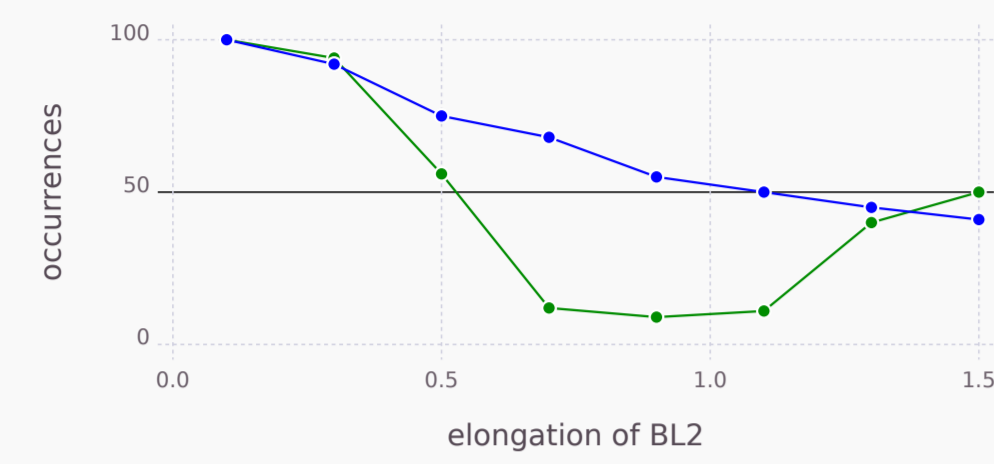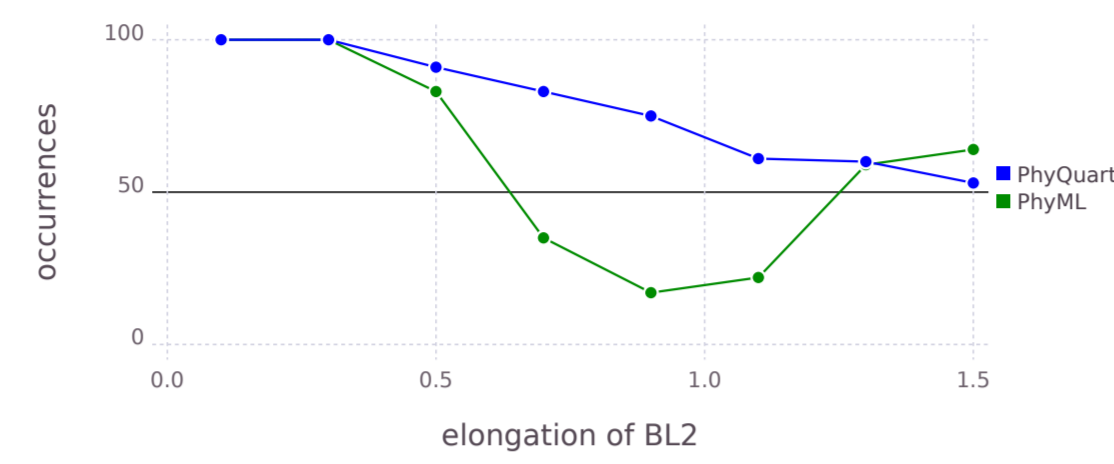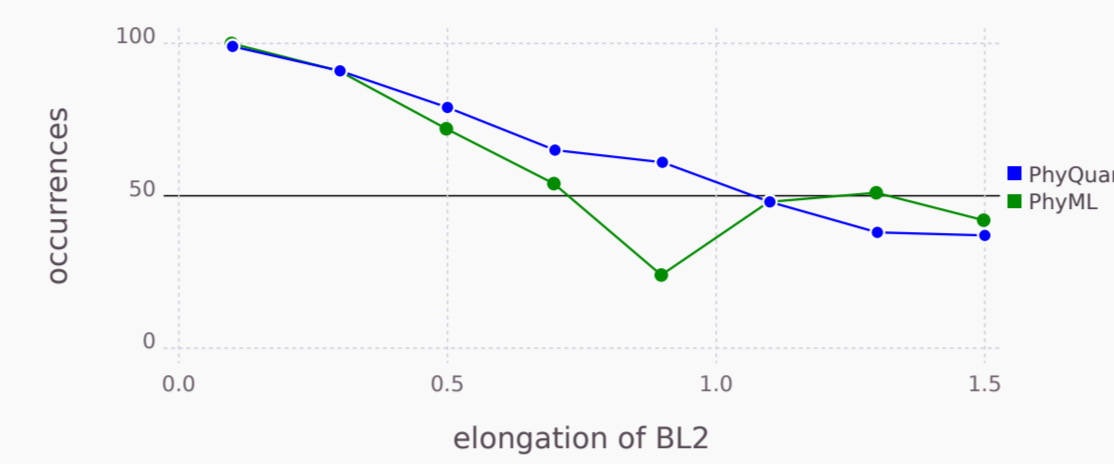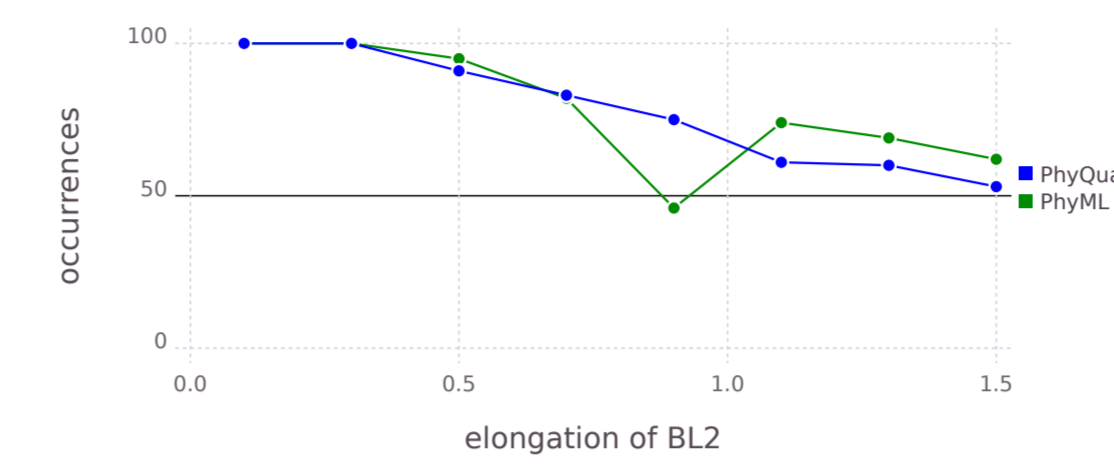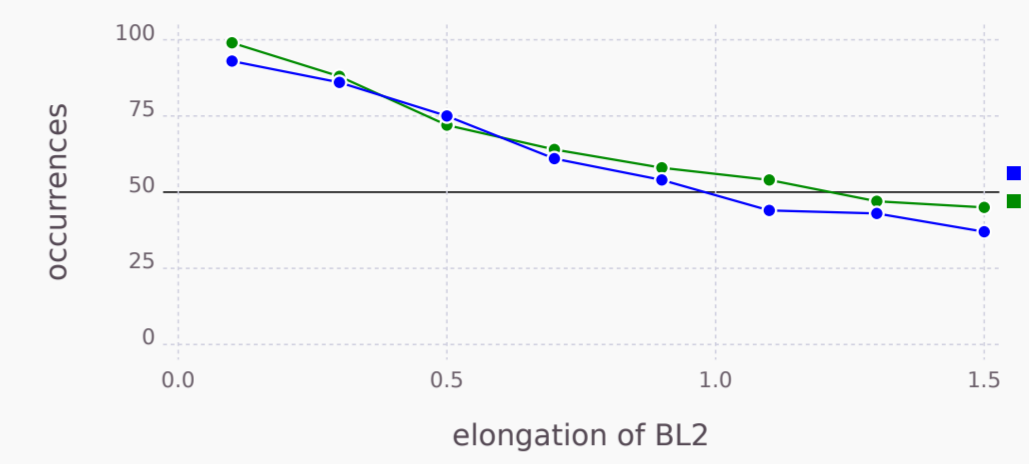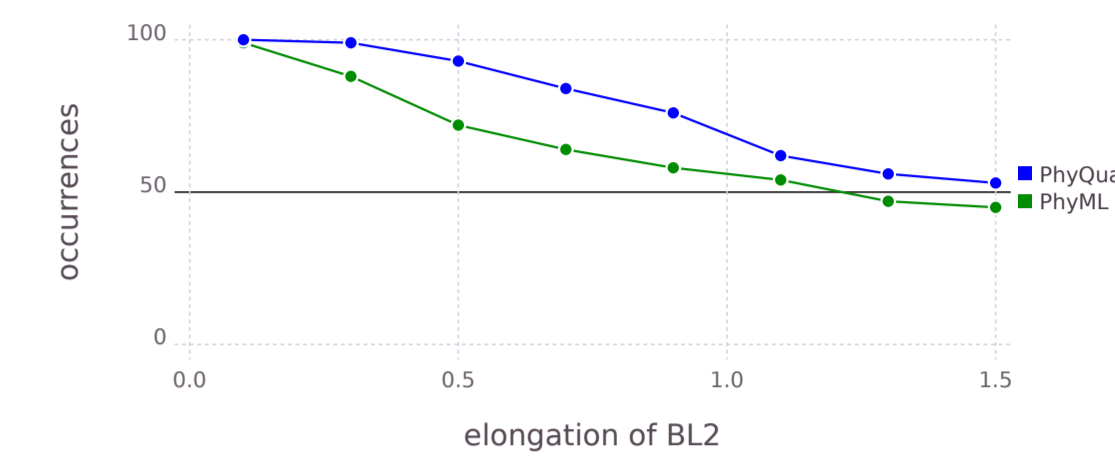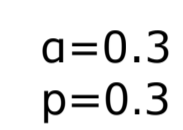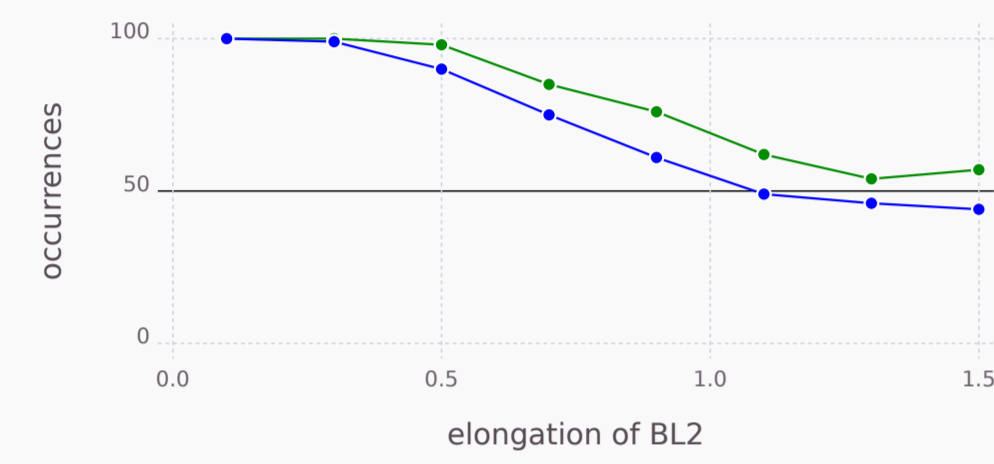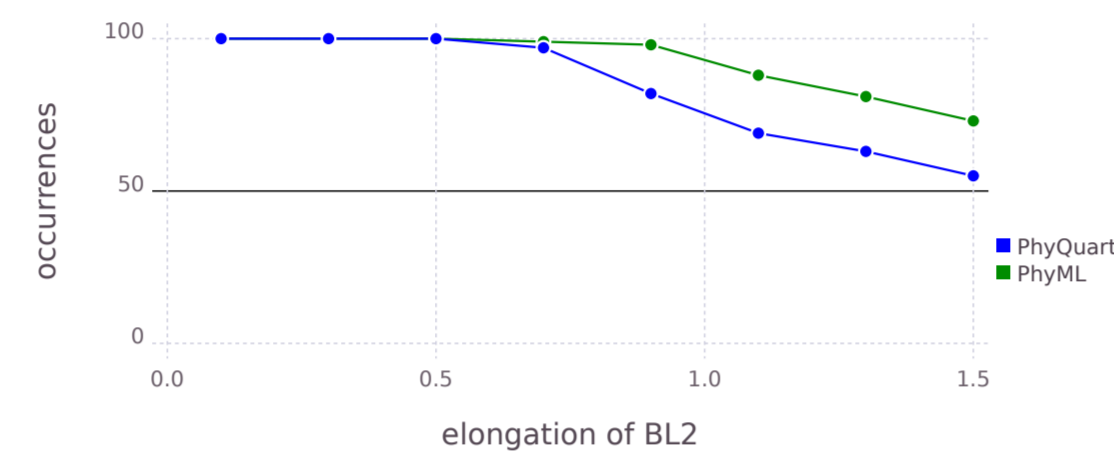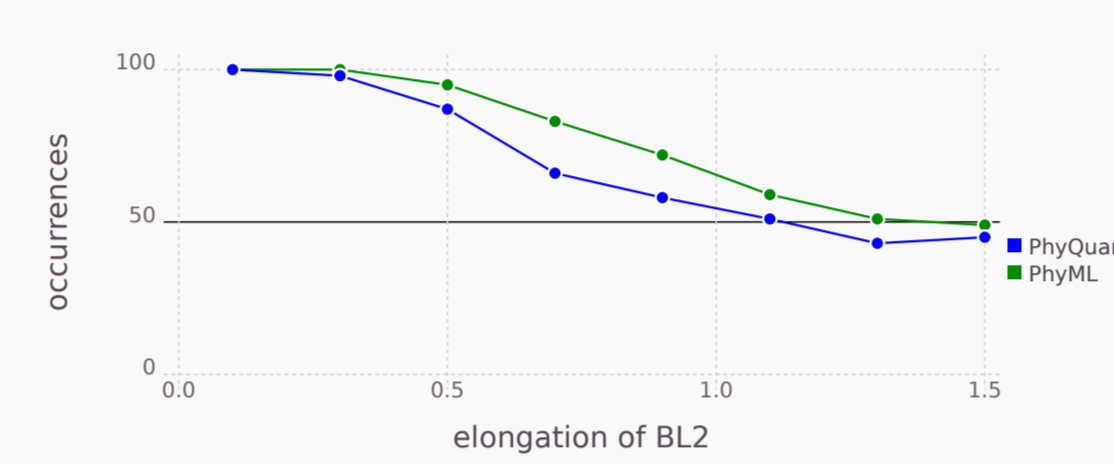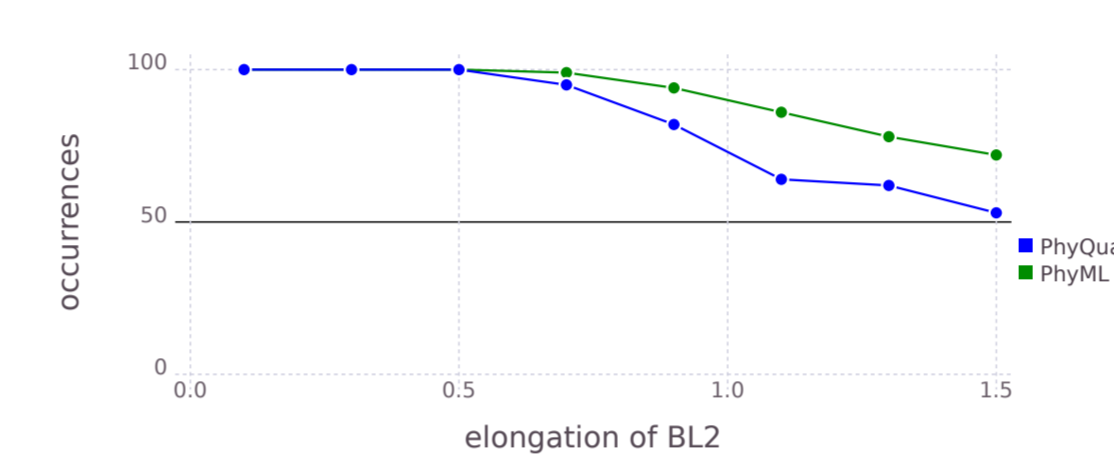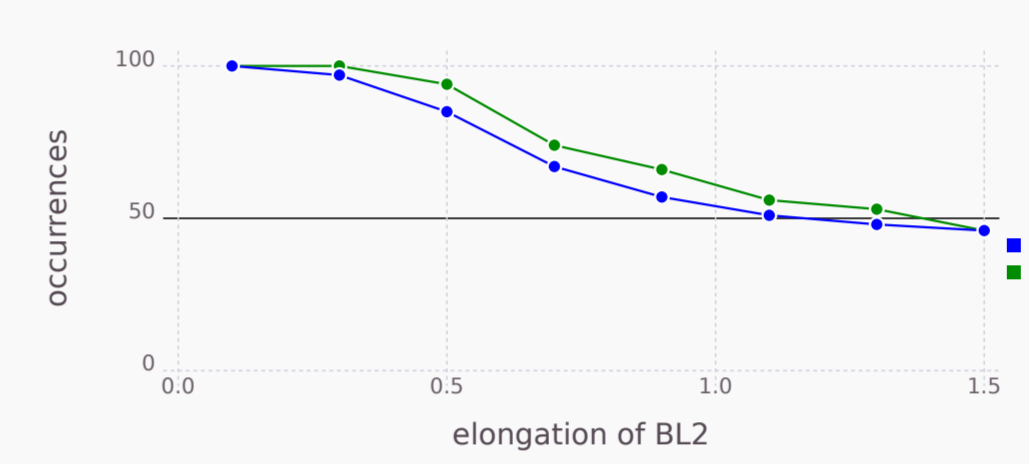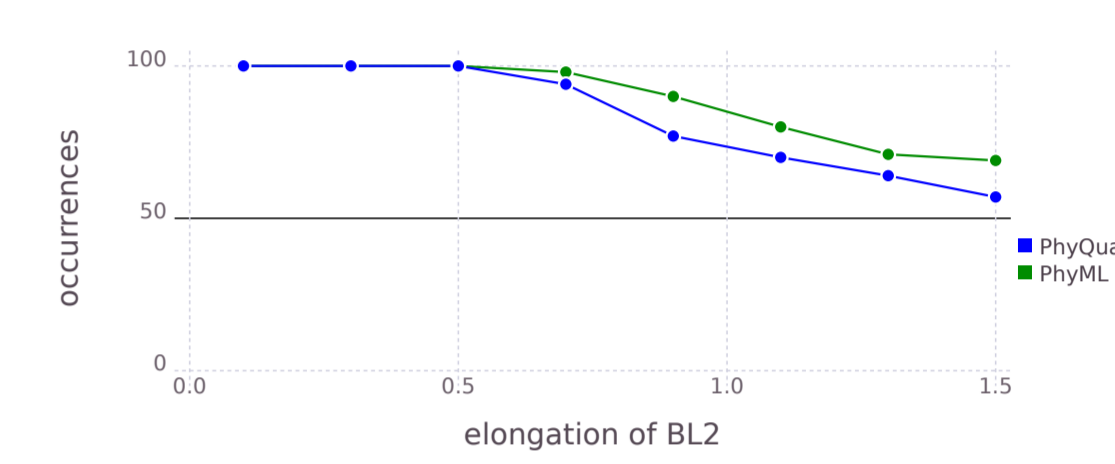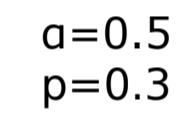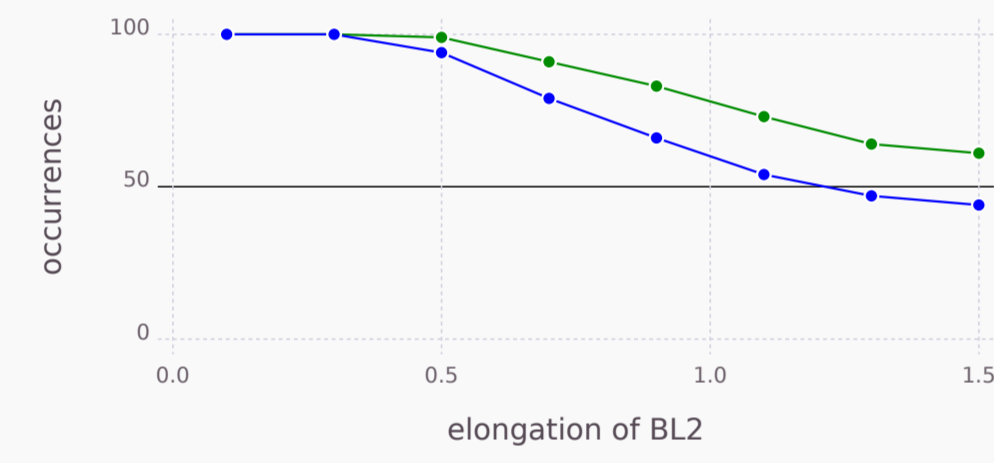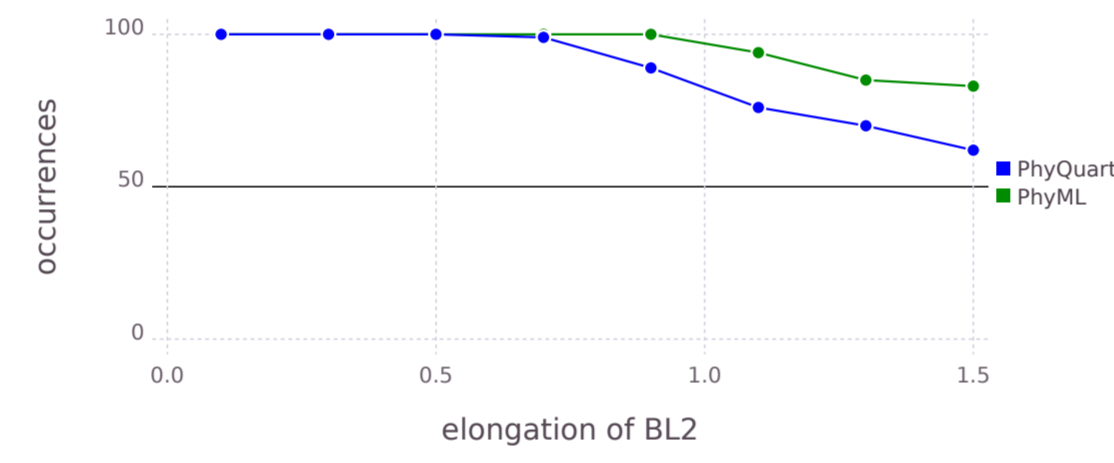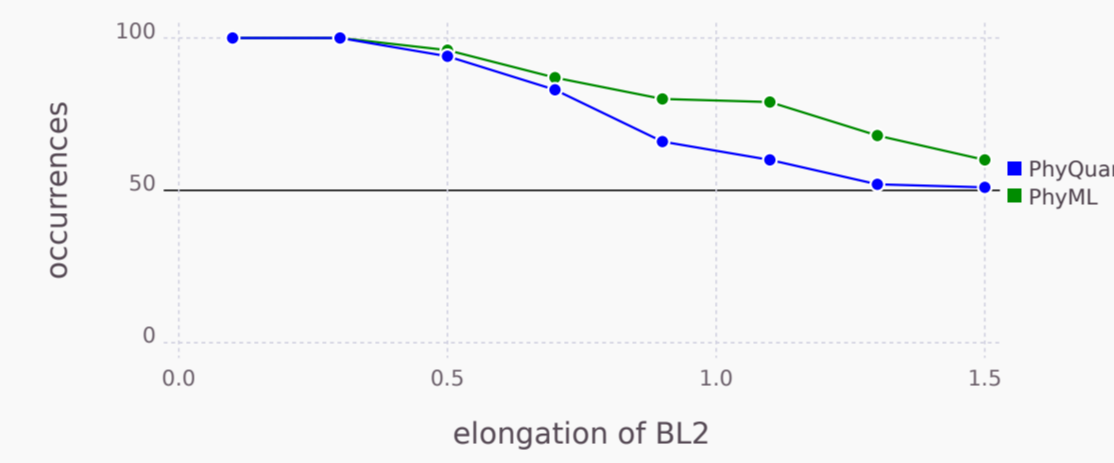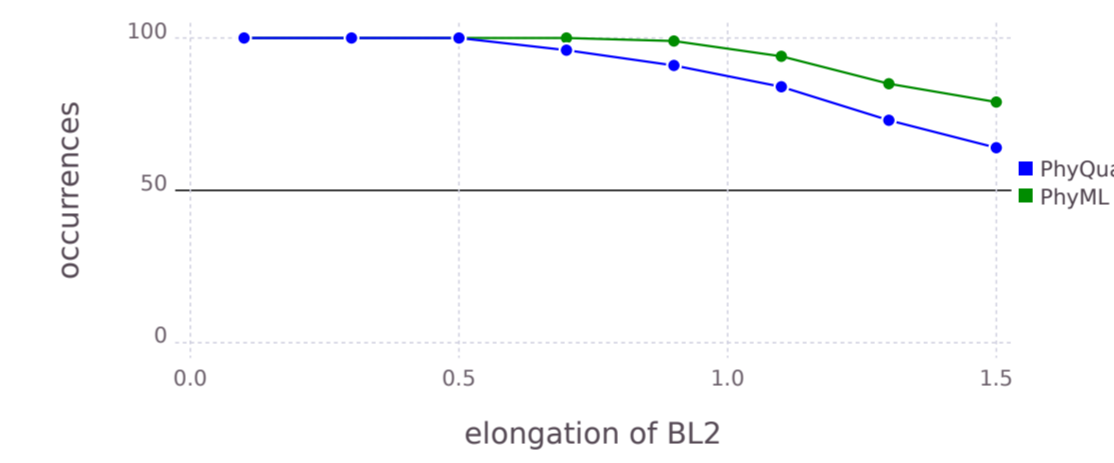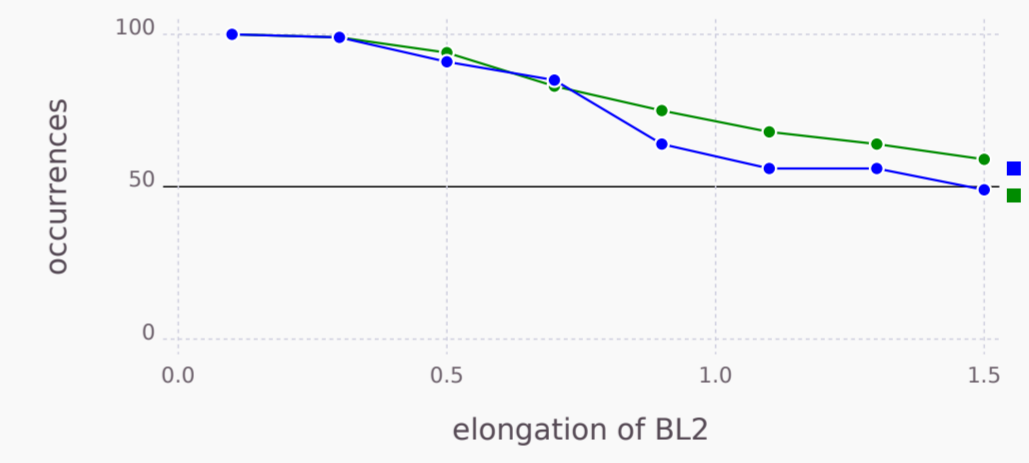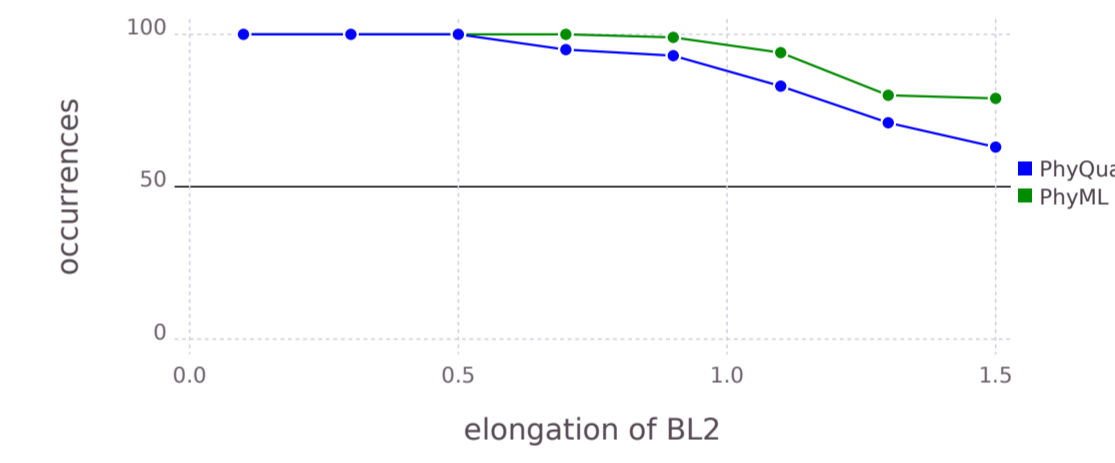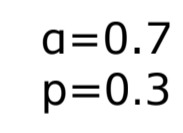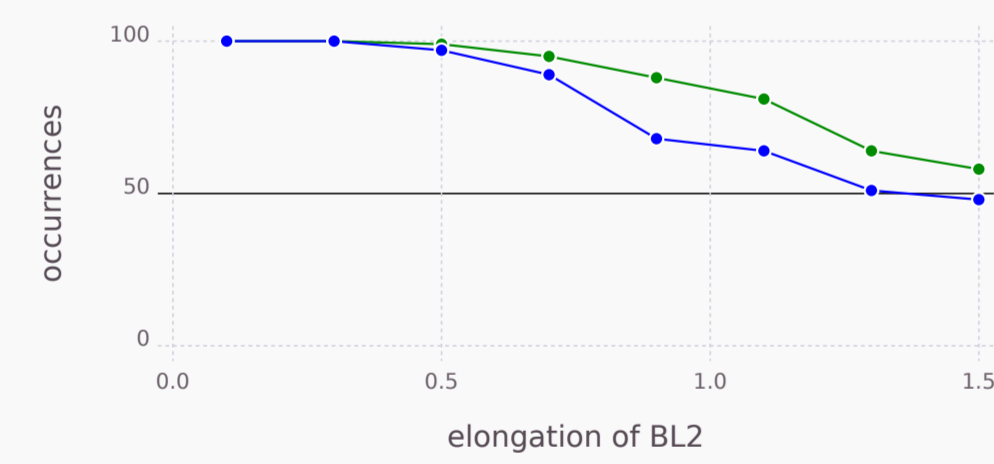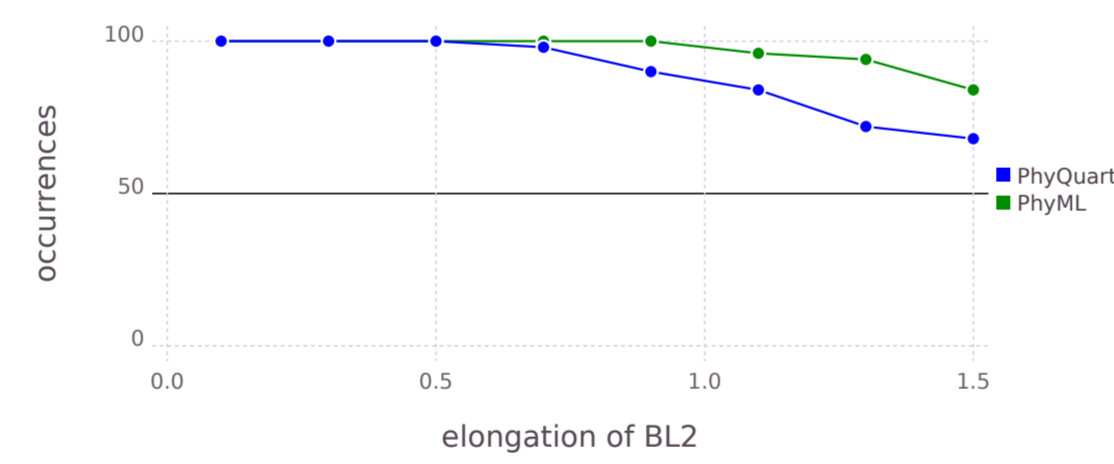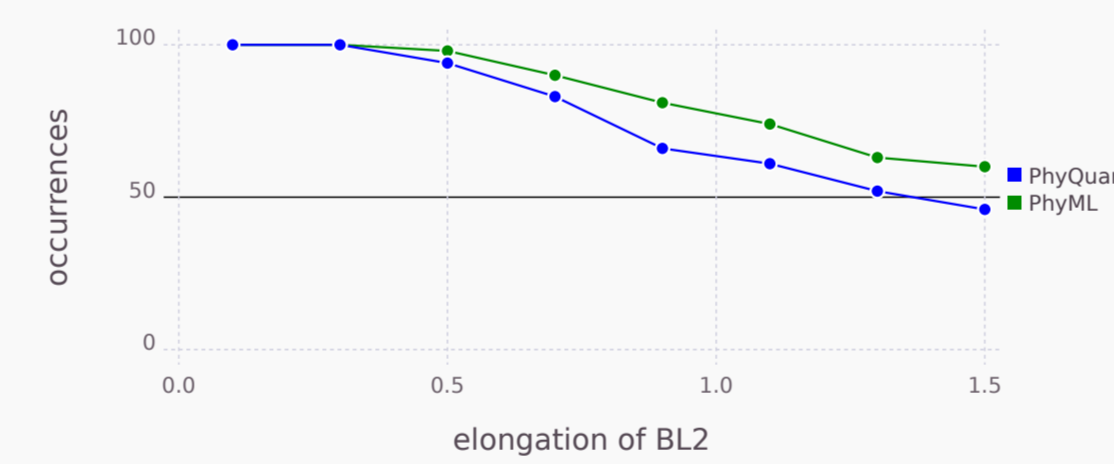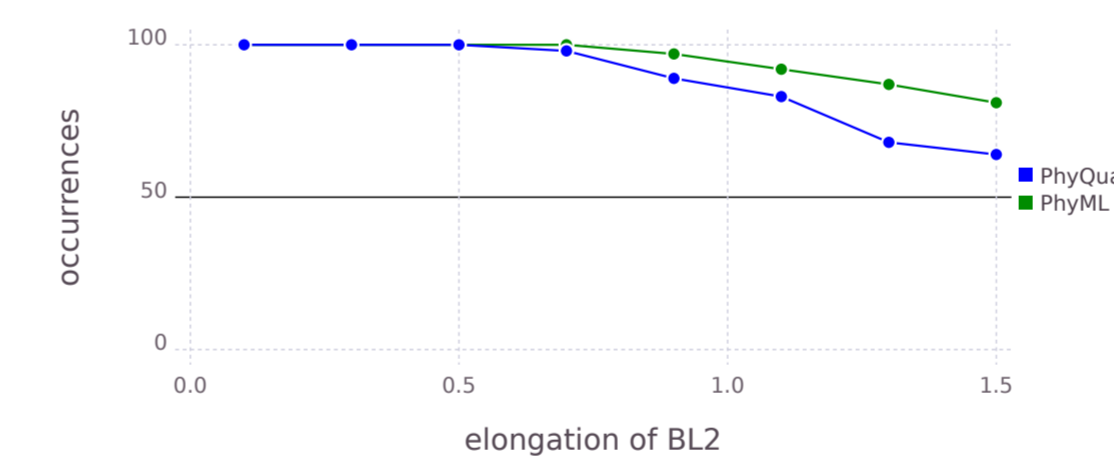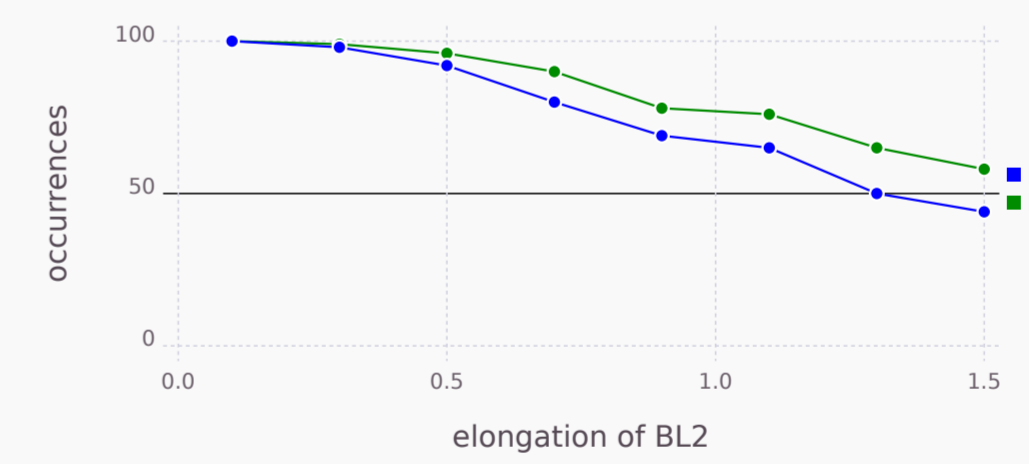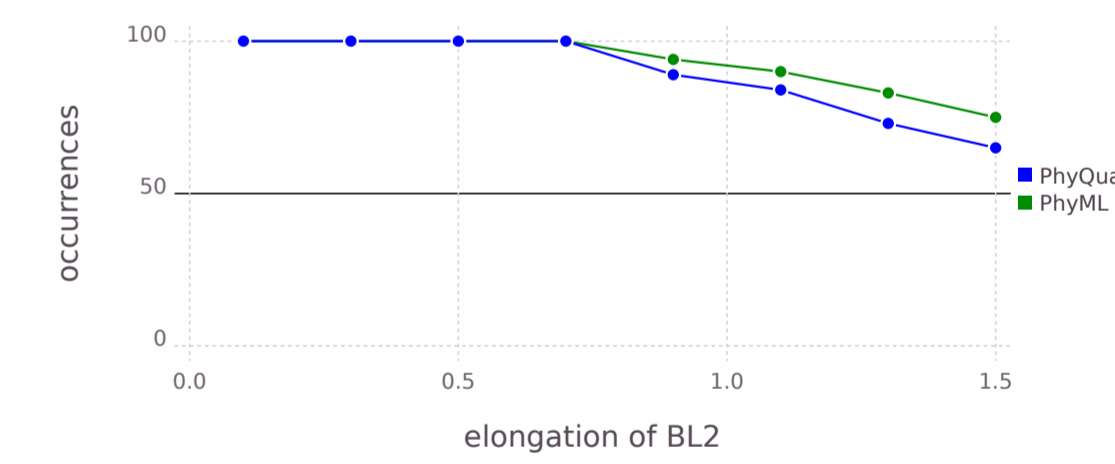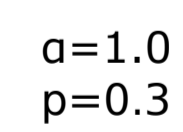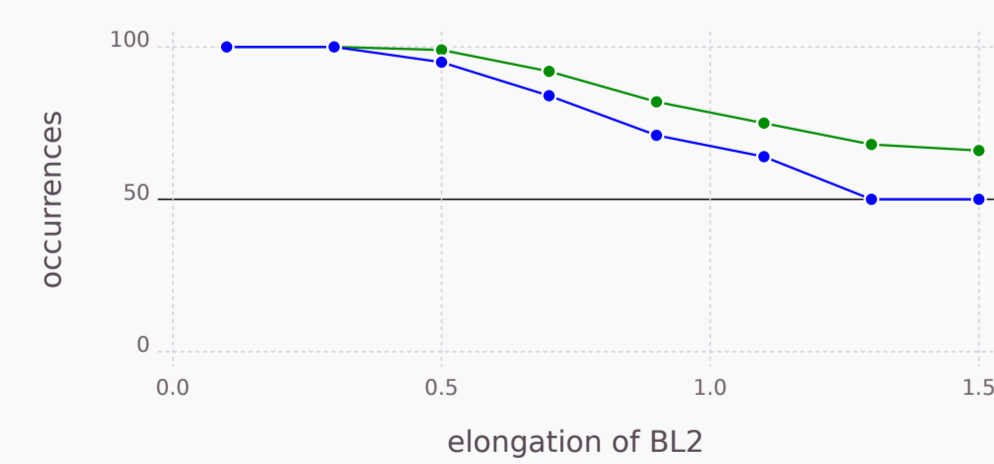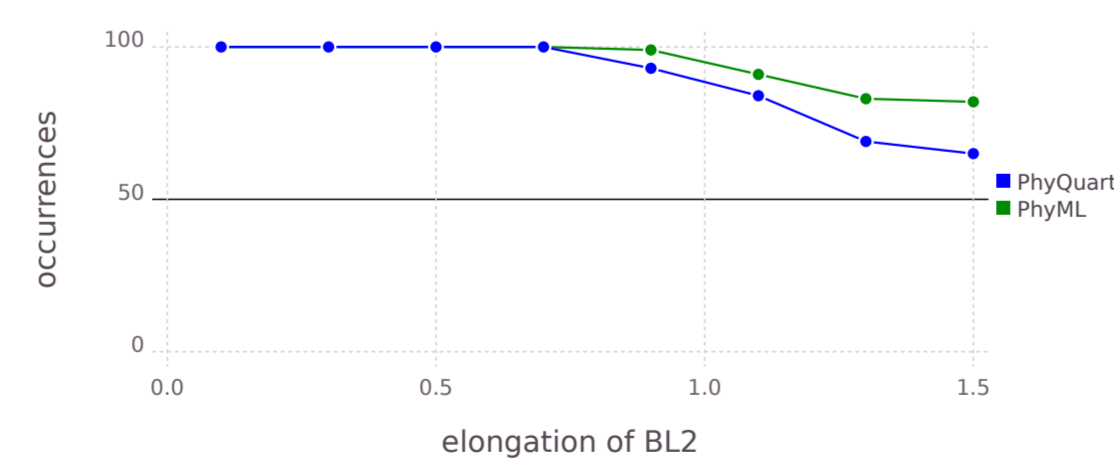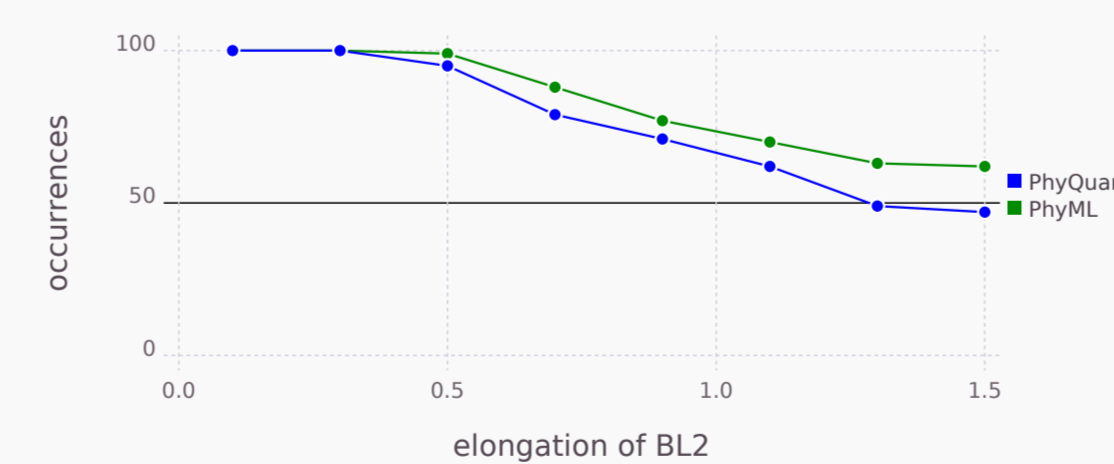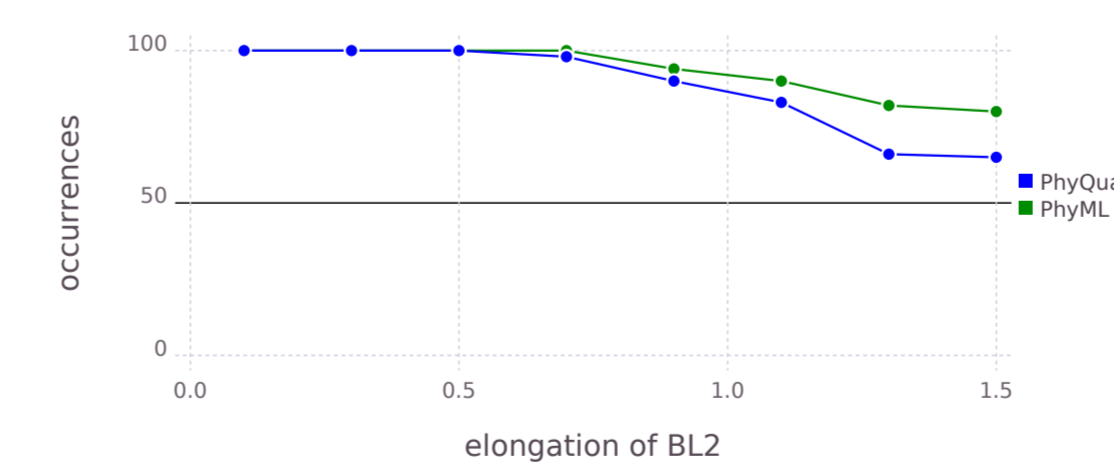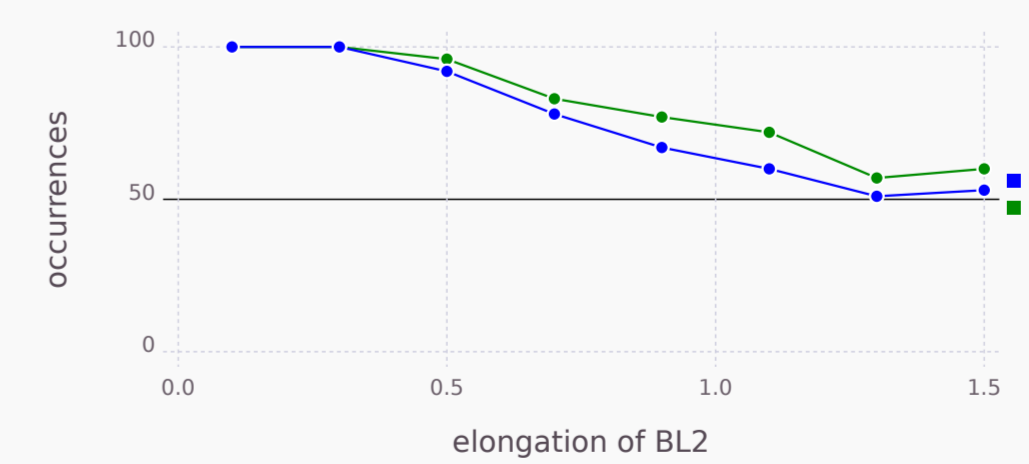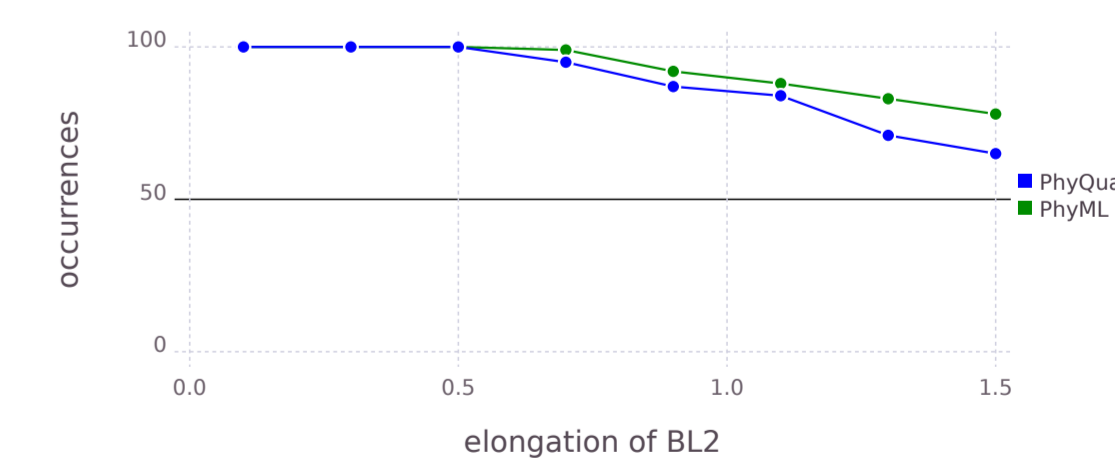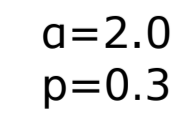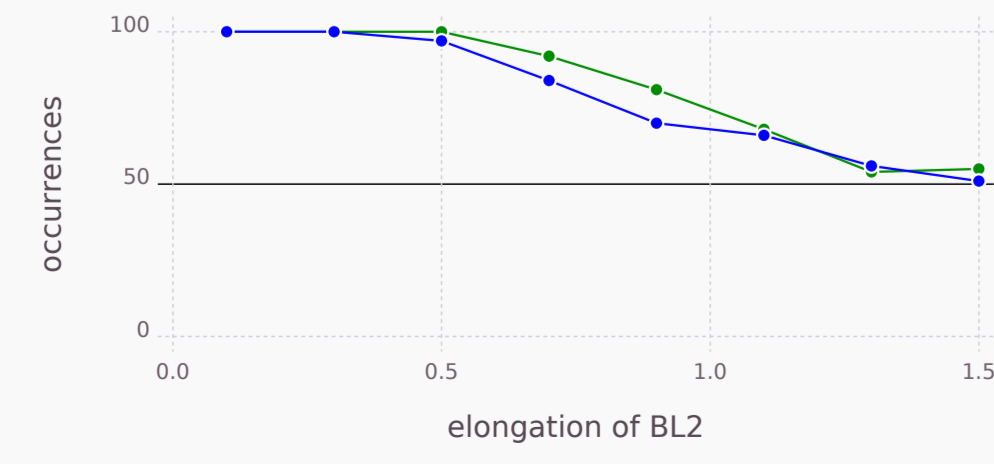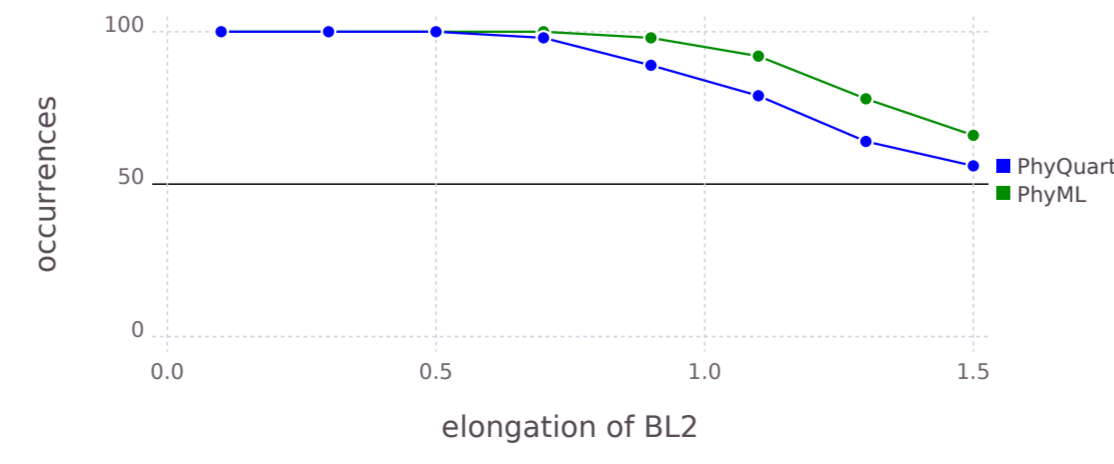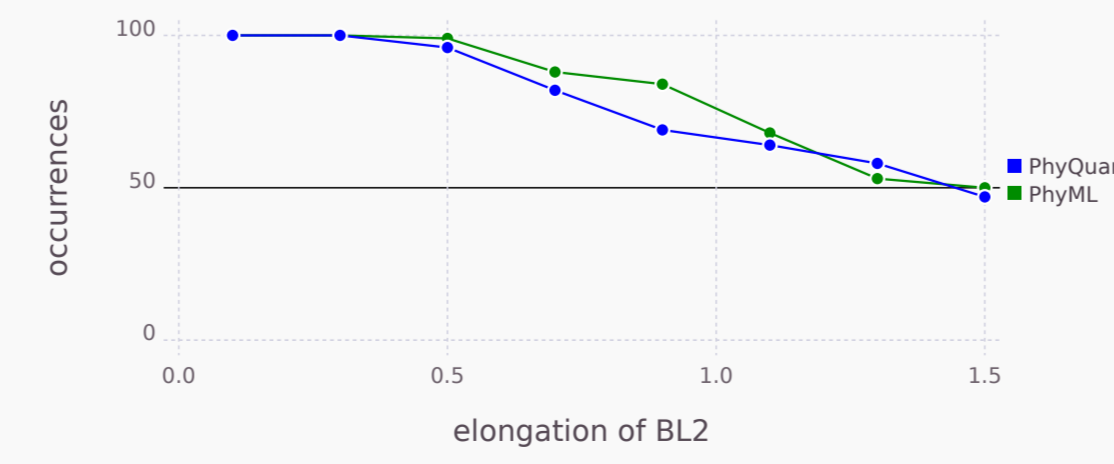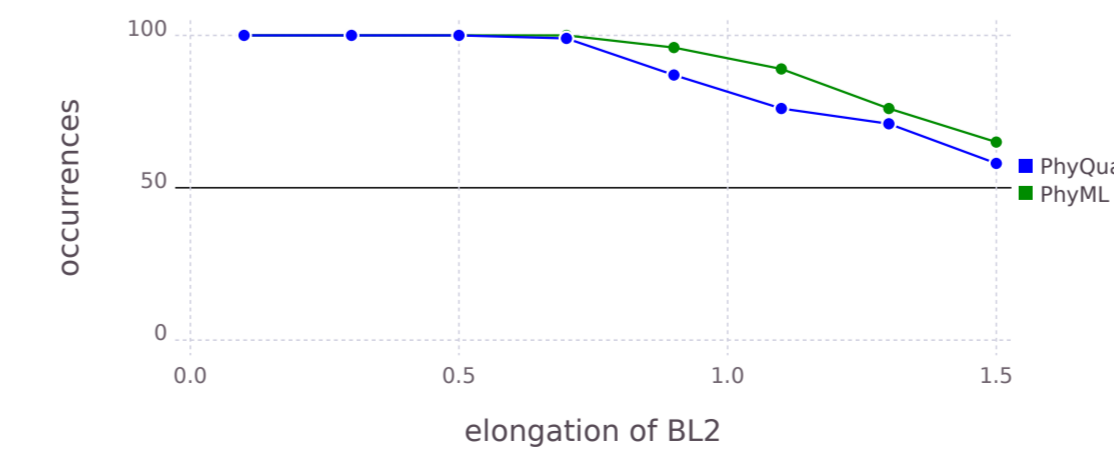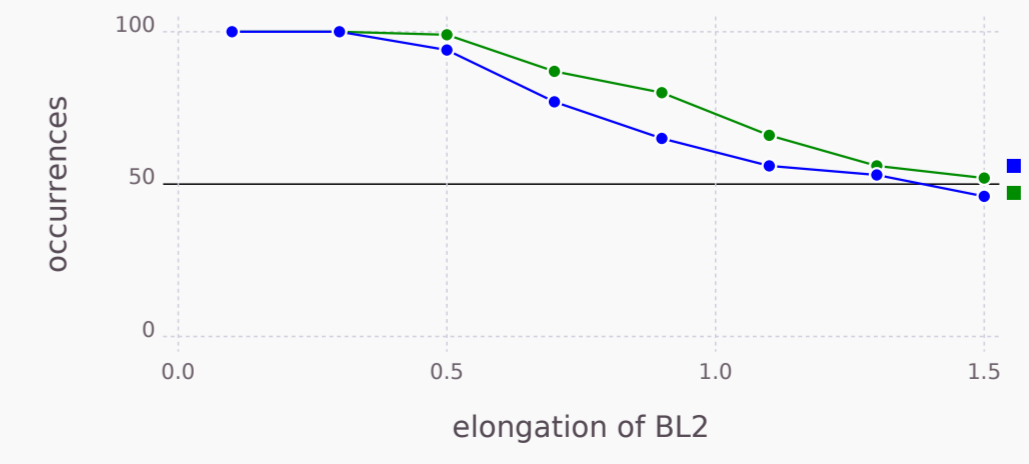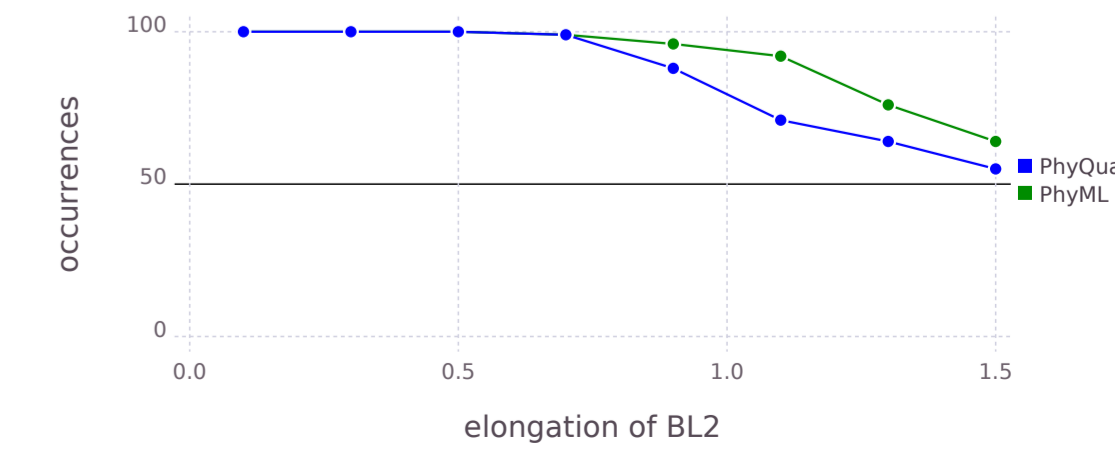

Supplement: S5 Fig — Complete results of 4-taxon simulations based on stepwise BL2 elongations of three terminal branches given 250 kbp long nucleotide alignment data. The pdf document can be opened with pdf readers like AdobeAcrobatReader, Xpdf, or DocumentViewer. (PDF) [file pone.0183393.s005.pdf]
