# Supplementary material for: Can quartet analyses combining maximum likelihood estimation and Hennigian logic overcome long branch attraction in phylogenomic sequence data?
Source: PLoS One. 2017 Aug 25;12(8):e0183393. doi: 10.1371/journal.pone.0183393 (PMC5571918; doi:10.1371/journal.pone.0183393)
Supplement: S3 Fig — Complete results of 4-taxon simulations based on stepwise BL2 elongations of two adjacent or non-adjacent terminal branches given nucleotide alignment data < 250 kbp. The pdf document can be opened with pdf readers like AdobeAcrobatReader, Xpdf, or DocumentViewer. (PDF) [file pone.0183393.s003.pdf]

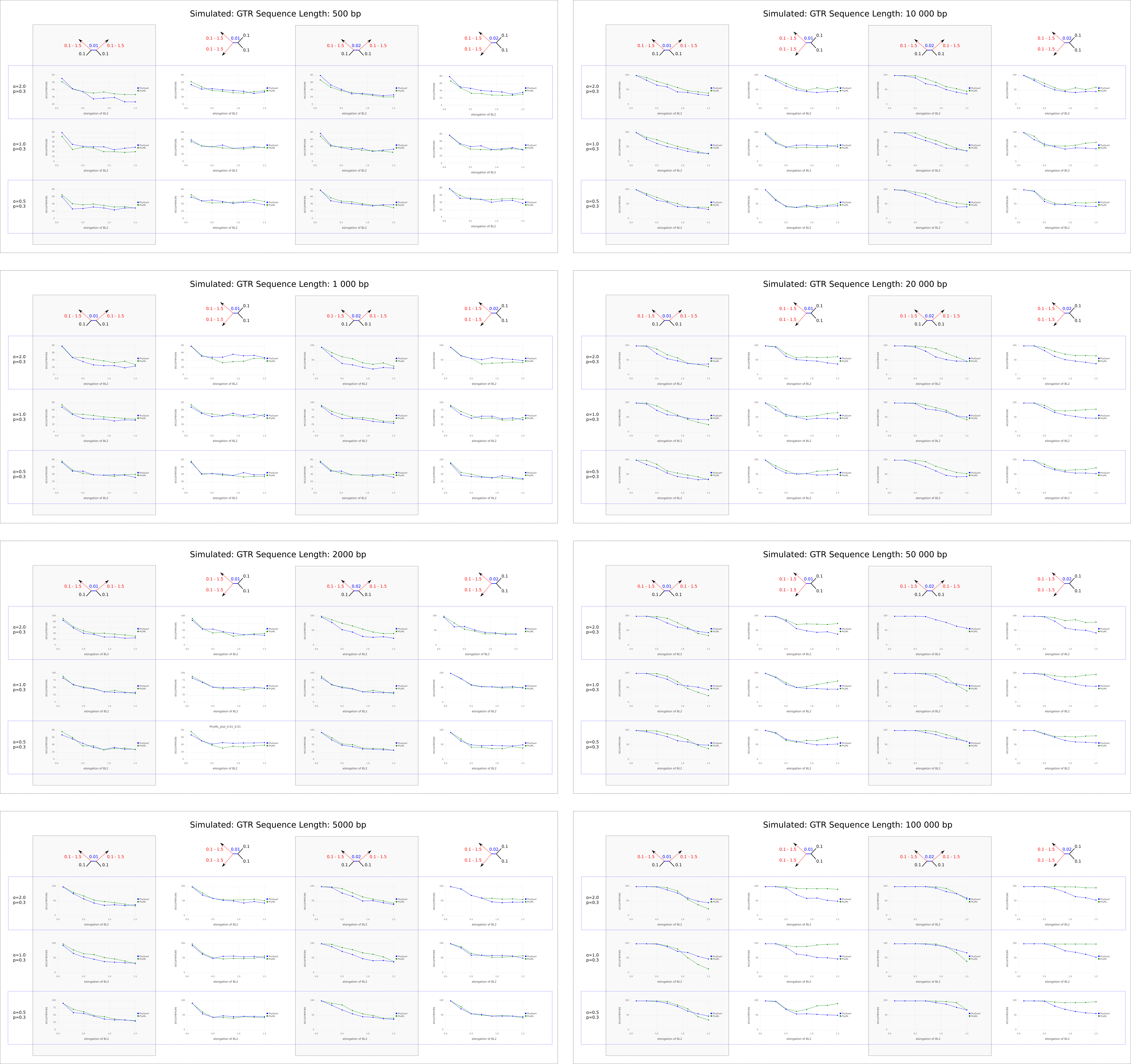

Simulated: GTR Sequence Length: 500 bp
Simulated: GTR Sequence Length: 10 000 bp
Simulated: GTR Sequence Length: 1 000 bp
Simulated: GTR Sequence Length: 20 000 bp
Simulated: GTR Sequence Length: 2000 bp
Simulated: GTR Sequence Length: 50 000 bp
Simulated: GTR Sequence Length: 5000 bp
Simulated: GTR Sequence Length: 100 000 bp
0.1 - 1.5
0.01
0.02
0.1
α=2.0
p=0.3
α=1.0
p=0.3
α=0.5
p=0.3
occurrences
elongation of BL2
PhyQuart
PhyML
PhyML_plot_0.01_0.01
